# Widespread 3′UTR capped RNAs derive from G-rich regions in proximity to AGO2 binding sites

**DOI:** 10.1186/s12915-024-02032-7

**Published:** 2024-11-07

**Authors:** Nejc Haberman, Holly Digby, Rupert Faraway, Rebecca Cheung, Anob M. Chakrabarti, Andrew M. Jobbins, Callum Parr, Kayoko Yasuzawa, Takeya Kasukawa, Chi Wai Yip, Masaki Kato, Hazuki Takahashi, Piero Carninci, Santiago Vernia, Jernej Ule, Christopher R. Sibley, Aida Martinez-Sanchez, Boris Lenhard

**Affiliations:** 1 MRC Laboratory of Medical Sciences, London, W12 0NN UK; 2 Institute of Clinical Sciences, Faculty of Medicine, Imperial College London, London, W12 0NN UK; 3 Division of Neuroscience, Department of Brain Sciences, Imperial College London, London, W12 0NN UK; 4 UK Dementia Research Institute at King’s College London, London, SE5 9RX UK; 5 Institute of Quantitative Biology, Biochemistry and Biotechnology, School of Biological Sciences, University of Edinburgh, Edinburgh, UK; 6 Section of Cell Biology and Functional Genomics, Department of Metabolism, Digestion and Reproduction, Imperial College London, London, W12 0NN UK; 7 RIKEN Center for Integrative Medical Sciences, Yokohama, Kanagawa 230-0045 Japan; 8 Human Technopole, Milan, 20157 Italy; 9 UCL Respiratory, Division of Medicine, University College London, London, WC1E 6JF UK; 10 The Francis Crick Institute, London, NW1 1AT UK; 11 Institute of Clinical Sciences, Faculty of Medicine, London, W12 0NN UK; 12 Institute of Biomedicine of Valencia (CSIC), Valencia, 46012 Spain

**Keywords:** 3′UTR, CAGE, Capping, AGO2, UPF1, 3′UTR-derived RNA, G-rich, Subcellular localisation

## Abstract

The 3′ untranslated region (3′UTR) plays a crucial role in determining mRNA stability, localisation, translation and degradation. Cap analysis of gene expression (CAGE), a method for the detection of capped 5′ ends of mRNAs, additionally reveals a large number of apparently 5′ capped RNAs derived from locations within the body of the transcript, including 3′UTRs. Here, we provide direct evidence that these 3′UTR-derived RNAs are indeed capped and widespread in mammalian cells. By using a combination of AGO2 enhanced individual nucleotide resolution UV crosslinking and immunoprecipitation (eiCLIP) and CAGE following siRNA treatment, we find that these 3′UTR-derived RNAs likely originate from AGO2-binding sites, and most often occur at locations with G-rich motifs bound by the RNA-binding protein UPF1. High-resolution imaging and long-read sequencing analysis validate several 3′UTR-derived RNAs, showcase their variable abundance and show that they may not co-localise with the parental mRNAs. Taken together, we provide new insights into the origin and prevalence of 3′UTR-derived RNAs, show the utility of CAGE-seq for their genome-wide detection and provide a rich dataset for exploring new biology of a poorly understood new class of RNAs.

**Graphical Abstract:**

Schematic representation of the proposed model where 3′UTR-derived RNAs originate from G-rich regions enriched in AGO2 and UPF1 binding sites. 
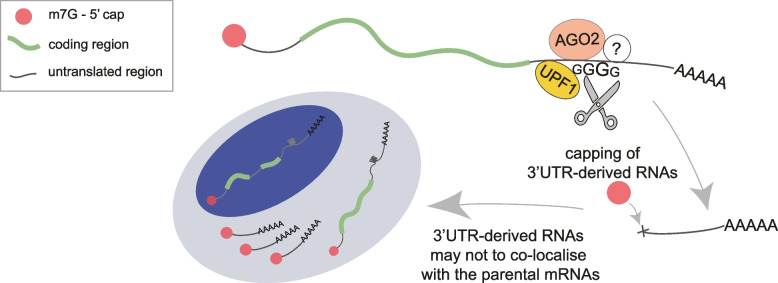

**Supplementary Information:**

The online version contains supplementary material available at 10.1186/s12915-024-02032-7.

## Background

In all eukaryotes, mRNA molecules contain an evolutionarily conserved m7G cap (N7-methylated guanosine), which is incorporated at the 5′ end of nascent transcripts. Co-transcriptional capping is the first modification made to nascent RNA in the nucleus, which protects it from exonuclease cleavage whilst promoting cap-related biological functions such as pre-mRNA splicing, polyadenylation and nuclear export [1]. In addition to the co-transcriptional capping, there is also evidence for a post-transcriptional capping mechanism, in which an m7G cap is added to newly exposed 5′ ends of RNA fragments created upon endonucleolytic cleavage or decapping [2–5]. However, little is known about the extent and biological role of this post-transcriptional capping and of its relation to other post-transcriptional RNA processing mechanisms.

Cap analysis of gene expression and deep-sequencing (CAGE-seq) was originally designed to precisely determine transcription start site (TSS) positions by capturing and sequencing 5′ ends of capped mRNA transcripts, and it can also be used to measure gene expression [6]. However, several studies have detected the unexpected, reproducible and so-far unexplained presence of CAGE signals (~ 10–15% of total reads) and/or an enrichment of RNA-seq reads mapping to 3′UTRs, far away from the usual TSS [7–15]. Previous studies have shown an absence of active promoter marks (i.e. no enrichment histone modifications or RNA polymerase II (RNAPII) occupancy) around these 3′UTR signals [7, 9, 16], arguing against the possibility that they are unannotated transcription start sites. Moreover, the expression of some of these capped 3′ UTRs is tissue-specific and regulated in mouse embryonic development, whilst their subcellular localisation can be separated from the associated protein-coding transcript, suggesting that their generation is a regulated process [16]. In addition, specific isolated 3′UTRs have been implicated in a growing number of physiological processes, such as cell signalling or oxidative stress [7, 17, 18]. Moreover, several capped 3′UTRs have been reported to play important roles in regulating protein expression in *trans*, similar to long non-coding RNAs [7, 9, 10]. Truncated mRNAs and RNA decay intermediates can be subject to post-transcriptional, cytosolic capping [2, 3, 15] and it has been suggested that a similar mechanisms may underlie the generation of some 3′UTR CAGE signals, referred to as 3′UTR-associated RNAs (uaRNAs) [16]. To avoid potential misunderstandings, we refer to these as 3′UTR-derived RNAs, as these newly generated RNAs are not known to be physically associated with 3′ UTRs.

Here, we thoroughly examine the presence of these 3′UTR-derived RNAs across the transcriptome and the molecular basis of their generation and characteristics. We perform a genome-wide identification of 3′ UTR-derived RNAs based on their capped 5′ ends and proceed to investigate the mechanisms involved in their formation. To this end, we combine CAGE, RNA-seq and cross-linking immunoprecipitation (CLIP)-based techniques from ENCODE and FANTOM consortia. We show that 3′UTR-derived RNAs present biochemical properties similar to their 5′ capped counterparts and that they can be as abundant as the protein-coding version of the host transcript, or even more so. We reveal that the 5′ ends of 3′UTR-derived RNAs are enriched in G-rich motifs and tend to form strong secondary structures, whilst the immediately upstream region of these 5′ ends is bound by UPF1 and/or AGO2. Moreover, some of these abundant 3′ UTR-derived RNAs exhibit a markedly different subcellular localisation profile than their protein-coding counterparts. Finally, we show, for the first time, that capped RNAs can also emerge following mRNA cleavage by small interfering RNAs (siRNAs).

## Results

### CAGE-seq identifies non-promoter associated capped 3′UTR-derived RNAs

**Fig. 1:**
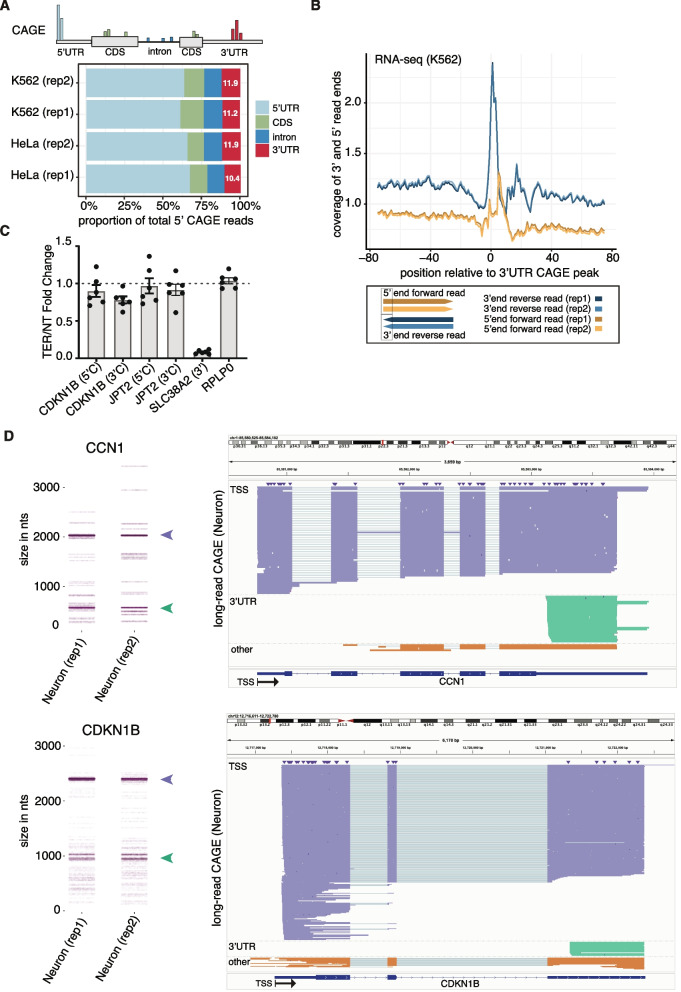
CAGE-seq identifies non-promoter associated capped 3′UTR-derived RNAs. **A** Top: Schematic representation of CAGE signals’ position across different transcript regions. Bottom: Bars indicate the proportion of total 5′ CAGE read positions per transcript region identified in CAGE-seq libraries of K562 and HeLa samples with two biological replicates each (rep1/2), provided by ENCODE (Additional file 2: Table S1—ENCSR000CJN and ENCSR000CJJ). **B** Top: Plot of the normalised coverage of the 5′ ends of forward paired-end reads (yellow lines) and 3′ ends of reverse paired-end reads (blue lines) of RNA-seq relative to 3′UTR CAGE peaks in K562 cells (Additional file 2: Table S1—ENCSR545DKY). Bottom: Schematic representation of paired-end read positioning. Forward and reversed paired-end reads are presented in yellow and blue, respectively, with the intensity of the colour indicating each of two biological replicates. The black box represents the ends of reads that are plotted in the top graph. **C** RT-qPCR data of gene expression ratios using primers amplifying regions immediately upstream (5′C) and downstream (3′C) of the 3′UTR CAGE peak, except for SLC38A2 whose 3′ cleavage site results in uncapped downstream fragment. Data is presented as a fold change of samples (six replicates) treated with TerminatorTM 5′-Phosphate-Dependent Exonuclease (TEX), which degrades 5′ monophosphate RNAs, versus non-treated (NT). Each dot represents the value of an independent biological replicate. **D** Long-read CAGE data (Additional file 2: Table S1—E-MTAB-14500) showing 3′ UTR-derived RNAs from CCN1 (above) and CDKN1B (below). Nanoblot plots (left) showing the range of read lengths at these genomic loci from two biological replicates in cortical neurons differentiated from induced pluripotent stem cells (rep1 and rep2), with long-read CAGE reads originating near the TSS (purple arrowhead) and those originating near the HeLa and K562 3′UTR CAGE peaks (green arrowhead) indicated. Genome browser visualisation (right) of the reads grouped and coloured in the same manner: 1_TSS (purple) originating near the TSS; 2_UTR (green) originating near the HeLa and K562 3′ UTR CAGE peaks and 3_OTHER (orange) originated at other sites

We and others [7–12] have previously reported the presence of CAGE-seq signals outside of annotated promoter regions in thousands of protein-coding genes. However, their origin or biological relevance has not been thoroughly interrogated. Here, we first confirmed the prevalence of these signals in human cell lines using CAGE data provided by the ENCODE consortium (Additional file 2: Table S1—ENCSR000CJN and ENCSR000CJJ). As expected, we detected a similar proportion of CAGE signals per genomic region in two different human cell lines (HeLa and K562) and showed that the CAGE signal is highly reproducible across replicates. This included the library size, number of uniquely mapped CAGE reads and distribution of the 5′ CAGE reads mapping to different genomic regions (Fig. 1A, Additional file 1: Fig. S1A, B, C, D). A similar genomic distribution has also been detected by other groups before, using the same CAGE-seq protocol [19].

The relative intensities of CAGE signal detected at different genomic regions depend on the priming method for reverse transcription (oligo-dT, random hexamers or mixtures thereof in different ratios) [12]. Oligo-dT priming quantitatively favours shorter transcripts, whilst the reverse is true for random priming. We subsequently verified CAGE signals (Additional file 2: Table S1—CAGE-seq (Oligo-dT ratios)) within 3′UTRs are most detected when a combination of Oligo-dT and random primers is used, with the optimal inclusion ratio of 1 to 4 ratio of Oligo-dT to random primers [19, 20] (Additional file 1: Fig. S1E). Notably, the same ratio was used in ENCODE CAGE samples analysed in this study.

The CAGE signal is the strongest at 5′UTRs of known protein-coding genes [19] (Fig. 1A, ~ 65% of total reads). Whilst low-level non-promoter CAGE signal (sometimes referred to as ‘exon painting’ ([4, 21]) can be detected along the entire length of transcripts, the signal at 3′UTRs is consistently present and occurs in localised clusters, similar to CAGE signals at promoters (see Additional file 1: Fig. S1L for examples). We focused on the 3′UTR region, which contains a substantial proportion (~ 11%) of the total CAGE reads (Fig. 1A), the implications of which are unknown. To identify robust CAGE signals with sufficient sensitivity, we used a 20-nt window requiring at least two 5′ reads overlapping from two different replicates for each cell line separately. This revealed 32,065 3′UTR CAGE clusters across all samples (Additional file 3: Table S2). As expected, correlation between technical replicates was high (Pearson correlation (PC) > 0.9) for all CAGE signals, independently of genomic location (Additional file 1: Fig. S1G–J). Moreover, expression of the 3′UTR CAGE clusters was highly reproducible between HeLa and K562 samples (PC ~ 0.98, Additional file 1: Fig. S1F), suggesting biological relevance. Correlation across cell types was much higher for 3′UTR clusters than that for the 5′ UTR CAGE (PC ~ 0.79, Additional file 1: Fig. S1G) and CDS CAGE (PC of 0.61, Additional file 1: Fig. S1H) signals and comparable to intronic CAGE signal (PC ~ 0.93, Additional file 1: Fig. S1I). This may suggest that 3′UTR CAGE signals originate from more stable and/or less tissue specific subset of transcripts. Together these analyses show that the transcripts whose 5′ end map to 3′UTR ends of protein-coding genes are highly reproducible across cell types and that CAGE is a robust method for their quantitative detection.

### 3′UTR-derived RNAs are confirmed by RNA-seq, qPCR and long-read CAGE

Next, we aimed to confirm the existence of these 3′UTR capped RNAs using independent methods. First, we asked if these fragments could be identified in transcriptomic (RNA-seq) data. For this we compared the CAGE signal with the RNA-seq signal of two different cell lines (Additional file 2: Table S1—ENCSR545DKY and GSE99169 [22]). To categorise CAGE peaks, we first used the paraclu [23] peak caller to identify clusters of 5′ ends of capped RNAs, and within each cluster we selected the highest signal as the dominant CAGE peak position. For comparison, we processed paired-end RNA-seq data from the same K562 and HeLa cell lines, then plotted read-starts and read-ends relative to the dominant 3′UTR CAGE peak per transcript (Fig. 1B—in blue and Additional file 1: Fig. S1J). Both RNA-seq samples showed highly reproducible enrichments of read ends coinciding with dominant 3′UTR CAGE peaks. This reveals that the 3′UTR CAGE peaks are confirmed by the read-ends from reverse-stranded RNA-seq data, which suggests that the signal could be originating from post-transcriptional cleavage sites. Notably, there is also a small enrichment of RNA-seq read-starts downstream from the 3′UTR CAGE peaks, which could represent the same RNA fragments detectable by the CAGE samples (Fig. 1B in yellow). More importantly, these findings demonstrate that 3′UTR capped fragments identified by CAGE can also be detected by other, methodologically independent, high-throughput sequencing methods such as RNA-seq.

We next aimed to confirm the presence of transcripts initiating at the 3′UTR CAGE peaks by an alternative experimental approach, not dependent on RNA library creation or high-throughput sequencing. We focussed on two genes, *CDKN1B*/p27kip1 (p27) and *JPT2*, which contain a dominant CAGE peak located within the 3′UTR region, demonstrated with highly reproducible read coverage for CAGE and RNA-seq in both K562 and HeLa cells (see Additional file 1: Fig. S1M for example). Separate sets of primers were designed to quantitatively PCR-amplify ~ 150-bp sequences within 300 nucleotides upstream and downstream of the 3′UTR CAGE peaks in *CDKN1B* and *JPT2* (see ‘Methods’, Additional file 1: Fig. S1K). In agreement with CAGE and RNA-seq data (Additional file 1: Fig. S1M), RT-qPCR detected higher levels of these transcripts with the downstream primers (Additional file 1: Fig. S1K) than with upstream primers. A similar enrichment in RT-qPCR signal (Additional file 1: Fig. S1K) was observed with downstream primers in comparison to primers designed to amplify a ~ 150-bp region spanning the CAGE peak in CDKN1B, suggesting an accumulation of 3′UTR fragments in comparison to full-length mRNAs.

Treatment of the samples with TerminatorTM 5′-Phosphate-Dependent Exonuclease (TEX), a 5′ → 3′ exonuclease that digests RNA with a 5′ monophosphate, but not RNA with 5′-triphosphate, 5′-cap or 5′-hydroxyl group had no or little effect on the amount of *JPT2* and *CDKN1B* transcripts detected with primers amplifying either side of the 3′UTR CAGE peak within these cells. This was in sharp contrast with the known uncapped 3′ fragment of *SLC38A2* mRNA, previously described by Malka et al. [8], which was, as expected, sharply reduced upon TEX treatment (Fig. 1C). These results lend further support that all the quantified transcripts, including the 3′UTR fragments, are capped.

We further confirmed that 3′UTR-derived RNAs could be detected by long-read nanopore-sequencing CAGE (Fig. 1D). We were provided with data in cortical neuron samples by the FANTOM6 consortium for 10 genes (Additional file 2: Table S1—E-MTAB-14500) that contain HeLa and K562 3′UTR CAGE peaks (Fig. 1D, Additional file 1: Fig. S1N). In all of the 10 examples, the full length read sequencing CAGE identified reads spanning from the start of our identified CAGE 3′UTR peaks till the end of the annotated transcripts, whereas for most of these genes, reads spanning between the 5′CAGE and the 3′CAGE signal were absent (Additional file 1: Fig. S1N). These observations suggest that the capped 3′UTR-derived RNAs originate from the full length mRNA whilst fragments upstream of the 3′CAGE may not be stable. Notable exceptions are DDX17 and GHITM but it is unclear whether these 3′CAGE upstream sequences result from alternative polyadenylation or are products from the cytosolic cleavage of the full-length.

### Capped 3′UTR-derived RNAs are evolutionarily conserved and generated post-transcriptionally

We next wanted to investigate whether the 3′UTR CAGE signals originate from post-transcriptionally capped RNA fragments. First, we explored whether there is evidence of nuclear cap-binding complex (CBC) binding to the capped 5′ ends of 3′UTR fragments, as this protein is known to bind to 5′ ends of nascent protein-coding mRNA transcripts in the nucleus. Individual-nucleotide resolution UV crosslinking and immunoprecipitation (iCLIP) is a method that identifies protein-RNA crosslinking interactions with nucleotide resolution in a transcriptome-wide manner. We examined CBC-iCLIP data from HeLa cells (Additional file 2: Table S1—GSE94427 [24]), where the authors targeted nuclear cap-binding subunit CBP20 protein [24]. CBP20 is a nuclear component of cap-binding complex (CBC), which binds co-transcriptionally to the 5′ cap of pre-mRNAs and interacts directly with the m7-G cap [25, 26]. The CBP20 RNA binding data was analysed using a standard iCLIP processing pipeline, where the nucleotide preceding the cDNA-start position after PCR duplicate removal is reported as the crosslinking position (see ‘Methods’). The CBP20 crosslinking positions were then screened across all dominant 5′UTR and 3′UTR CAGE peaks per transcript. As expected, CBP20 crosslinks were enriched around the dominant 5′UTR CAGE peaks where the TSS of full-length transcripts is positioned. However, the enrichment was very weak at the non-promoter 3′UTR CAGE peaks (Additional file 1: Fig. S2A). This strongly indicates that the 3′UTR capped fragments identified by CAGE are not part of nuclear CBC, further suggesting that they are likely a product of an independent post-transcriptional processing pathway.

To further explore whether the 3′UTR capped fragments were generated co-transcriptionally, we investigated the location of cap signals in nascent RNAs identified in global nuclear run-on sequencing experiments of 5′ capped RNAs (GRO-cap) (Additional file 2: Table S1—ENCSR363AKK [27]). As anticipated, strong GRO-cap signals overlapped with CAGE peaks in 5′UTRs and, to a lesser extent, with introns and upstream CDSs but were notably absent around 3′UTR CAGE peaks (Additional file 1: Fig. S2B). This also indicates that capping of 3′UTR fragments occurs post-transcriptionally.

Additionally, we analysed capCLIP data from HeLa cells (Additional file 2: Table S1—GSE138473 [28]). capCLIP is a version of CLIP that targets the translation elongation factor eIF4E, a cytoplasmic protein which binds the 7-methyl-GTP moiety of the 5′-cap structure of RNAs to facilitate the efficient translation of almost all mRNAs [28, 29]. The capCLIP data was analysed following the same methodology as CBP20-iCLIP. The enrichment of capCLIP signal at the non-promoter 3′UTR CAGE peaks was much stronger than in the CBC-iCLIP (Additional file 1: Fig. S2A, C), which demonstrates that the cap of the 3′UTR-derived RNAs is strongly bound by cytoplasmic eIF4E, but not the nuclear cap binding protein CBP20, suggesting that these RNAs are predominantly cytoplasmic. Furthermore, we investigated ribosome footprinting data [30] to interrogate whether the 3′UTR-derived RNAs, which are bound by eIF4E, are translated. However, we did not find evidence of ribosomal binding to these RNAs that suggested active translation (data not shown).

Next, we investigated the evolutionary conservation of 3′UTR-derived RNAs. Utilising UCSC conservation tracks, we computed conservation scores around 3′UTR CAGE peaks. To exclude the influence of coding regions and transcript termination sites, we specifically selected 21,831 3′UTR CAGE peaks positioned at least 150 bps away from the 3′UTR bordering region (≥ 150 bps downstream from CDS and ≥ 150 bps upstream from transcript termination). Remarkably, our findings reveal that the exact 3′UTR CAGE peaks exhibit lower conservation compared to the surrounding regions. However, the region immediately downstream of the 3′UTR CAGE peaks, corresponding to the ‘body’ of 3′UTR-derived RNAs, shows a notable enrichment in conservation scores, suggesting a potential functional contribution (Additional file 1: Fig. S2D).

Altogether, these analyses confirm the presence of abundant, evolutionarily conserved, capped 3′UTR-derived non-coding RNAs that may originate from cytosolic cleavage of full-length mRNAs.

### 5′ ends of 3′UTR-derived RNAs are enriched for G-rich motifs and strong secondary structures

Next, we wanted to understand the sequence features that distinguish the CAGE peaks corresponding to co-transcriptional capping of TSS from those originating from post-transcriptional capping of 3′UTR-derived RNAs. We first explored the possibility that 3′UTR fragments might be a by-product of nuclear polyadenylation and associated endonucleolytic cleavage. If this were the case, the identified 3′UTR CAGE peaks should be preceded by enrichment of the canonical polyA signal (A[A/U]UAAA hexamers), which recruit the nuclear polyadenylation machinery. However, we only found such enrichment at the annotated 3′UTR ends and not upstream of the 3′UTR CAGE peaks (Additional file 1: Fig. S2E). We observed a notable enrichment downstream of the 3′UTR CAGE peaks (Additional file 1: Fig. S2E—red line), which most likely corresponds to the canonical polyA site as some of the 3′UTR-derived RNAs are relatively short and their 5′ ends are close to the annotated 3′UTR ends.

**Fig. 2:**
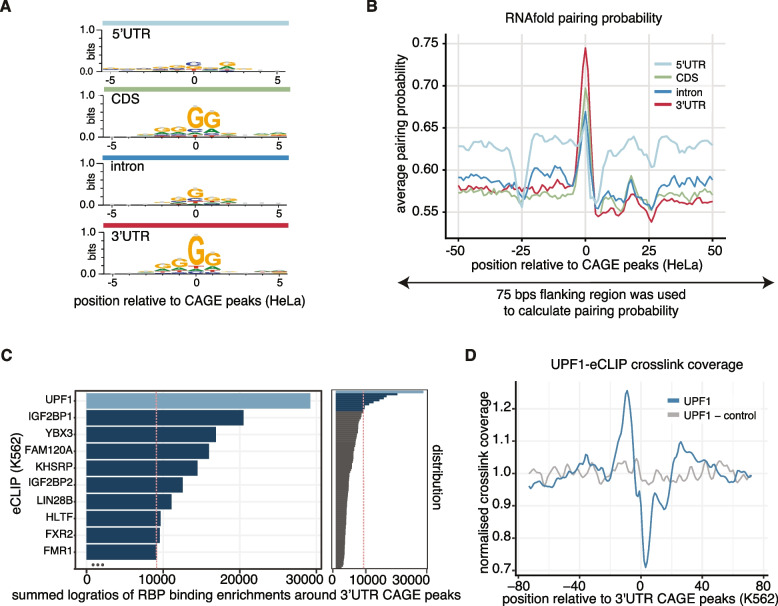
5′ ends of 3′UTR-derived RNAs are enriched in G-rich motifs and strong secondary structures and flanked by UPF1 binding sites. **A** Sequence logos around HeLa cells’ CAGE peaks across different transcript regions. **B** The 75-nt region centred on HeLa cells’ CAGE peaks at different transcript regions was used to calculate pairing probability with the RNAfold program, and the average pairing probability of each nucleotide is shown for the 50-nt region around CAGE peaks. **C** Enrichment of eCLIP cross-linking clusters surrounding 3′UTR CAGE peaks from 80 different RBP samples (right-hand side panel) in K562 cells from the ENCODE database (Additional file 2: Table S1—all eCLIP samples) using sum of log2 ratios of crosslink enrichments. The red line represents the threshold of top 10 RBP targets which are presented in detail in the left-hand side panel. **D** RNA-map [32] showing normalised density of UPF1 crosslink sites (Additional file 2: Table S1—ENCSR456ASB) relative to 3′UTR CAGE peaks (blue, UPF1) and random positions of the same 3′UTRs as control (grey, UPF1-control) in K562 cells

Next, we explored whether there were additional distinctive sequence characteristics between the two types of CAGE peaks. Consistent with previous studies [9, 13], we detected a strong G-enrichment overlapping the 5′ end of the CAGE reads present in non-promoter regions (Fig. 2A, Additional file 1: Fig. S2F), distinct from the YR dinucleotide characteristic of signals at 5′ ends of genes. More surprisingly, CAGE peaks within the 3′UTR region showed a strong increase in internal pairing probability (see ‘Methods’: ‘Secondary structure’) in comparison to CAGE peaks in other regions (Fig. 2B, Additional file 1: Fig. S2G), suggesting that structural preference may be important for the generation of 3′UTR-derived RNAs. Notably, the surrounding (within 100 bps) region of CAGE peaks in 5′UTRs is more structured (light blue line in Fig. 2B, Additional file 1: Fig. S2G), representing the higher GC content that is present around all 5′UTRs in vertebrates [31], with a distinctive drop at − 25 bps coinciding with the canonical TATA box position.

Motifs with G-rich repeats in the transcriptome can form non-canonical four-stranded structures (G4s) implicated in transcriptional regulation, mRNA processing, the regulation of translation and RNA translocation [33]. Similar to web-logo motif analyses of CAGE peaks from different mRNA regions (Fig. 2A, Additional file 1: Fig. S2F), the nucleotide enrichment plot of GGG sequences showed the highest enrichment overlapping 3′UTR CAGE peaks (Fig. 2C, Additional file 1: Fig. S2F). This raises the possibility that the sequence around the 3′UTR CAGE peaks may have an increased propensity to form RNA-G4 structures via the canonical G4 motif (G_3_-N_1-7_-G_3_-N_1-7_-G_3_-N_1-7_-G_3_) [34]. To further explore the RNA G-quadruplexes formation profile, we integrated RNA-G-quadruplex sequencing (rG4-seq) data from HeLa cells (Additional file 2: Table S1—GSE77282 [35]) and ran G4-Hunter predictions [36] around CAGE peaks. Both the rG4-seq data (HeLa) and G4-Hunter predictions (K562) showed the highest G4s enrichment around CAGE peaks in the 3′UTR region (Additional file 1: Fig. S2H–L) with the highest percentage of rG4-seq hits within 3′UTRs (Additional file 1: Fig. S2M). Nevertheless, it is worth noting that the number of 3′CAGE sites overlapping with rG4-seq sites was relatively small (~ 3800 out of ~ 133,900). In sharp contrast, 8 of the 10 gene examples with 3′UTR CAGE peaks and validated with long-read CAGE explored here (selected due to highest 3′UTR CAGE signal) contained rG4-seq clusters coinciding with 3′UTR CAGE peaks (Additional file 1: Fig. S1N). It is important to note that the determination of whether these sites are genuinely in the G4-folded state remains uncertain, as the rG4-seq method employs G4 stabilisers to artificially enhance G4 structures.

### 3′UTR CAGE sites are flanked by enriched UPF1 binding

The evidence outlined so far is consistent with our hypothesis that capped 3′UTR-derived RNAs are formed post-transcriptionally. Next, we aimed to determine whether specific RNA-binding proteins (RBPs) were involved in the mechanism of their generation. To that end, we analysed publicly available enhanced CLIP (eCLIP) data for 80 different RBPs in the K562 cell line (Additional file 2: Table S1—all eCLIP samples), produced by the ENCODE consortium [37]. For each RBP, we calculated normalised cross-linking enrichment compared to other RBPs around maximum CAGE peaks per annotated gene region (5′UTR, CDS, intron, 3′UTR). This identified a specific set of RBPs around CAGE peaks, with UPF1 (Up-frameshift protein 1) as the top candidate in 3′UTRs (Fig. 2C), DDX3X (DEAD-Box Helicase 3 X-Linked) in 5′UTRs (Additional file 1: Fig. S2N), KHSRP (KH-type splicing regulatory protein) in introns (Additional file 1: Fig. S2O), and less protein specific enrichments in CDS with YBX3 (Y-Box-Binding Protein 3) as the top candidate (Additional file 1: Fig. S2O, P). UPF1 is involved in a variety of RNA degradation pathways [38], including nonsense-mediated decay (NMD) [39] and the normal mRNA decay where stalled UPF1 at CUG and GC-rich motifs activates decay [40]. KHSRP plays a well-characterised role in pre-mRNA splicing, but has also been involved in several other aspects of RNA biology, such as mRNA decay and editing and maturation of miRNA precursors [41]. On the other hand, the YBX3 has been implicated in regulation of mRNA translation as well as stability, likely in a transcript-dependent manner [42]. As a positive control for our enrichment score approach, we noted DDX3X enrichment around 5′UTR CAGE peaks. This is consistent with known roles for DDX3X in transcription and pre-mRNA splicing through interactions with transcription factors and spliceosomal B complexes [43].

Interestingly, the crosslinking of UPF1 is enriched within 20 nts upstream of the 3′UTR CAGE peaks, followed by a steep depletion within ~ 10 bps downstream (Fig. 2D). Additionally, a substantial correlation (*R* = 0.654) was observed between the 3′UTR CAGE signal and UPF1 binding, but not associated with gene expression or 3′UTR length (Additional file 1: Fig. S2Q). More specifically, the degree of UPF1 binding coincides with the intensity of the 3′UTR CAGE peaks and proximity to the peaks (Additional file 1: Fig. S2R, S). However, transfecting K562 cells with UPF1-targeting small interfering RNAs (siRNAs) for 48 h did not lead to changes in the enrichment of RT-qPCR signal obtained with primers targeting downstream of the 3′UTR CAGE peak in *CDKN1B* or *JPT2* when compared to upstream-targeting primers (Additional file 1: Fig. S2T). Thus, it remains unclear if the precise binding position of UPF1 relative to the re-capping position may be important for the generation of the 3′UTR capped fragments, or if accumulation of UPF1 is an indirect result of the presence of other factors that contribute to the cleavage.

### mRNA cleavage by small interfering RNAs generates newly capped RNA fragments

mRNAs can be cleaved post-transcriptionally through RNA interference (RNAi). Indeed, a common way to artificially accomplish gene silencing is to utilise siRNAs to induce endonucleolytic degradation of the target transcripts [44, 45]. siRNAs are usually 21–23 nt long and their sequence is antisense to their mRNA target sequence. Silencing by siRNAs is induced through the endonuclease activity of Argonaute 2 (AGO2), a subunit of the RNA-induced gene-silencing complex (RISC) in the cytoplasm [46].

**Fig. 3:**
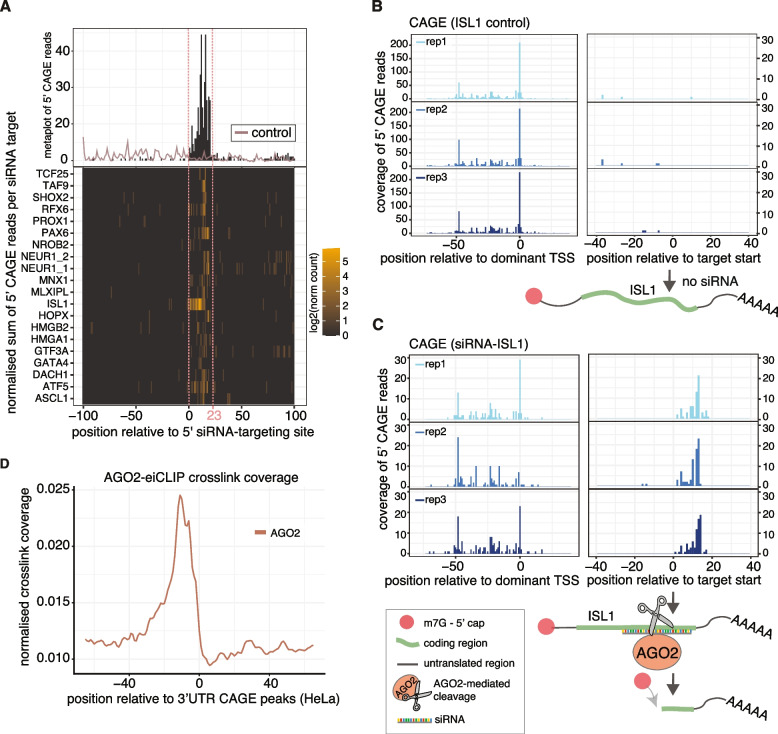
Capping at small interfering RNAs (siRNAs) target sites. **A** Enrichment of 5′ CAGE reads relative to 5′ sites of small interfering RNAs in TC-YIK cells (Additional file 2: Table S1—siRNA-KD CAGE [47]) transfected with siRNAs targeting 20 different mRNAs (with IDs indicated on the left-hand side of the bottom graph) and merged control samples (control). The heatmap represents log2 of read counts, normalised by the mean of all counts within 200 nts of the targeting site. **B**, **C** Enrichment of 5′ CAGE reads relative to the correspondent dominant transcription start sites (TSS) (left-hand side panels) and to 5′ end of the *ISL1* mRNA sequence targeted by the siRNA (right-hand side panels) in samples treated with an siRNA targeting *ISL1* (**C**, siRNA-ISL1) or with non-targeting siRNA controls (**B**, control siRNAs). Three biological replicates are shown per treatment. Visual representations of the capped, full-length *ISL1* mRNA in the absence of *ISL1*-targeting siRNAs (**B**, control siRNAs) versus both the capped, full-length and the capped, cleaved fragment in the presence of *ISL1*-targeting siRNAs (**C**, siRNA-ISL1), are shown below the correspondent panels. **D** RNA-map [32] showing normalised density of eiCLIP-AGO2 crosslink sites relative to 3′UTR CAGE peaks in HeLa cells (Additional file 2: Table S1—E-MTAB-12945)

We hypothesised that siRNA silencing through AGO2 cleavage could lead to cytoplasmic capping of the cleaved RNA fragments instead of degradation. To test this hypothesis, we first investigated if CAGE-seq could detect cleaved RNA fragments guided by siRNA. We analysed CAGE data from siRNA-treated samples from the FANTOM5 dataset (Additional file 2: Table S1—siRNA-KD CAGE [47]), which included samples from the TC-YIK human cell line transfected with siRNAs targeting mRNAs of 28 different transcription factors (20 siRNAs designed by ThermoFisher and 8 by the study authors) and 5 non-targeting control samples, in triplicates. We detected CAGE signal at the exact position targeted by the siRNA in at least two replicates in 20 out of the 28 samples (Fig. 3A). The strongest enrichment in CAGE signal relative to the siRNA target site was detected in the Islet-1 knockdown (*ISL1*-KD) samples, with no signal detected in control samples (Fig. 3B, C, Additional file 1: Fig. S3A). More interestingly, the dominant CAGE 5′ end signal was present in the middle of the siRNA target sequence (Fig. 3E, Additional file 1: Fig S3A), where the AGO2 cleavage is known to take place [48, 49]. As expected, the TSS CAGE signal in the 5′UTR of the corresponding protein-coding gene dropped by ~ 75% compared to the control samples in all 3 replicates (Fig. 3B, C), confirming that the silencing of the *ISL1* transcript was efficient. Together these results indicate that siRNA-mediated recruitment of AGO2 can lead to the generation of post-transcriptionally capped RNA fragments following mRNA cleavage.

### 3′UTR CAGE peaks coincide with AGO2 and UPF1 binding sites alongside G-rich motifs

Since the endonuclease activity of AGO2 facilitates mRNA cleavage guided by siRNAs, we investigated whether AGO2 binding also occurred at the endogenous 3′UTR CAGE peaks. There was no publicly available AGO2 binding data for either HeLa or K562 cells so we produced ‘enhanced individual nucleotide resolution’-CLIP (eiCLIP) [50] data for AGO2 (AGO2-eiCLIP) in HeLa cells (Additional file 2: Table S1—E-MTAB-12945). Our analysis revealed that 32.8% of the crosslinking positions mapped to the 3′UTR region (Additional file 1: Fig. S3B), with a higher binding enrichment in known microRNA (miRNA)-regulated transcripts and with a clear miRNA-seed matching-sequence enrichment downstream of the crosslinking site (Additional file 1: Fig. S3C, D). Similarly to UPF1, AGO2 crosslinks were enriched immediately upstream from the 3′UTR CAGE peaks but, unlike UPF1, they were not depleted in the downstream region (Fig. 3D, Additional file 1: Fig. S3E, Fig. 2D).

In animals, endogenous RNAi is mainly mediated by microRNAs (miRNAs). MiRNAs are ~ 21–23 nucleotide (nt) long RNAs that, in contrast to siRNAs, recruit the miRNA induced silencing complex (miRISC) containing AGO1–4 to mRNAs with partial sequence complementarity. As a result, miRNA action induces translational repression and/or exonucleolytic cleavage of the target mRNAs [44, 45]. Thus, miRNA-mediated degradation of target mRNAs in animals usually involves deadenylation, decapping and degradation by the major cytoplasmic 5′-to-3′ exonucleases, rather than direct endonucleolytic cleavage by AGO2 [5, 51]. Nevertheless, it has been demonstrated that extensive miRNA-mRNA pairing can also trigger AGO2 catalytic activity [52–54]. We thus hypothesised that AGO2 miRNA-guided cleavage of mRNA targets might lead to the generation of recapping fragments in a similar manner to that observed for siRNAs. To test this, we first identified genomic sequences with extensive complementarity (fewer than 2 mismatches) to human miRNAs. We identified 29 such targets that mapped within 3′UTRs but there was no CAGE signal present around any of them (data not shown). In line with this, AGO2 crosslinking enrichment around 3′UTR CAGE signals was considerably weaker for AGO2 binding sites overlapping with predicted miRNA binding sites (see ‘Methods’, Additional file 1: Fig. S3F). Intriguingly, CRISPR/Cas9-mediated elimination of AGO2 in K562 cells did not change the enrichment in RT-qPCR signal detected for *JPT2* and *CDKN1B* with primers downstream the 3′CAGE versus upstream primers (Additional file 1: Fig S3G–I). All together, our observations suggest that AGO2 binds immediately upstream of the site of cleavage that generates 3′UTR-derived RNAs independently of miRNA directed recruitment and that endogenous 3′UTR-derived RNAs are not produced as a result of AGO2 cleavage activity.

Accordingly, we instead explored the binding specificity of AGO2-eiCLIP data and performed a motif analysis using HOMER motif finder. When analysing the 15-bp flanking region around AGO2-crosslinking peaks (see ‘Methods’), one of the most prominent motifs was highly enriched in Gs (Additional file 1: Fig. S3J—2nd and 3rd). Notably, this also agrees with one of the first AGO2-CLIP studies performed on mouse embryonic stem cells, where the authors showed that, without the miRNA present, AGO2 binds preferentially to G-rich motifs [55].

As we had previously demonstrated that G-rich motifs, which have the capability to form RNA-G-quadruplexes, are enriched around 3′UTR-derived RNAs, we next investigated whether AGO2 and UPF1 could be attracted to these specific G-rich motif structures independently of their location to CAGE peaks. We first aligned AGO2-eiCLIP and UPF1-eCLIP cross-linking positions relative to the 3′ end of rG4-seq sites in different regions of primary transcripts. Both AGO2 and UPF1 crosslink-binding sites are much more highly enriched at rG4-seq sites in the 3′UTRs relative to 5′UTRs, introns and coding sequence although we noted that the binding of UPF1 occurred at the 3′end of the G4-seq sites and AGO2 bound immediately upstream (Additional file 1: Fig. S3K–N). To further explore the relationship between AGO2 and UPF1 binding concerning 3′UTR CAGE peaks and their association with G4-seq signals, we categorised the 3′UTR CAGE peaks into four classes, depending on the presence or absence of these elements. We observed that the majority of 3′UTR CAGE peaks contained both AGO2 and UPF1 binding but not G4-seq signal although, on the other hand, the majority of 3′UTR CAGE sites overlapping with G4-seq also contained AGO2/UPF1 sites (Additional file 1: Fig. S3O). Then, we further explored the binding position of UPF1 and AGO2 relative to the 3′UTR CAGE peaks, in the presence or absence of the G4-seq site. Interestingly, both proteins exhibited a distinct shift in position influenced by the G4 motif enrichment; whilst AGO2 showed a pronounced shift to the upstream region of the 3′UTR CAGE peak in the presence of G4-seq sites, UPF1 displayed a downstream shift (Additional file 1: Fig. S3P–Q). An important next direction for future studies will be to experimentally investigate the mechanistic implications of the overlap between sites with the ability to form RNA-G4 structures and AGO2 and UPF1 binding for the generation of the capped 3′UTR-derived RNAs.

### Capped 3′UTR fragments of CDKN1B and JPT2 transcripts do not co-localise with the parental mRNAs

Finally, we examined the potential implications of 3′UTR-derived RNAs. Specifically, we sought to understand how 3′UTR-derived RNAs might localise either together or independently from the parental mRNAs. To test this, we designed smFISH (single molecule fluorescence in situ hybridisation) probes to simultaneously image the RNA upstream and downstream of the proposed post-transcriptional cleavage and capping site in CDKN1B and JPT2 using hybridisation chain reaction RNA-fluorescence in situ hybridisation (HCR-FISH 3.0) [56]. To account for technical biases in detection, we also designed probes against the coding sequence (hereafter upstream) and 3′UTR (hereafter downstream) of a control mRNA, PGAM1, which does not contain CAGE peaks in the 3′UTR and contained a similar 3′UTR length to our targets.

**Fig. 4:**
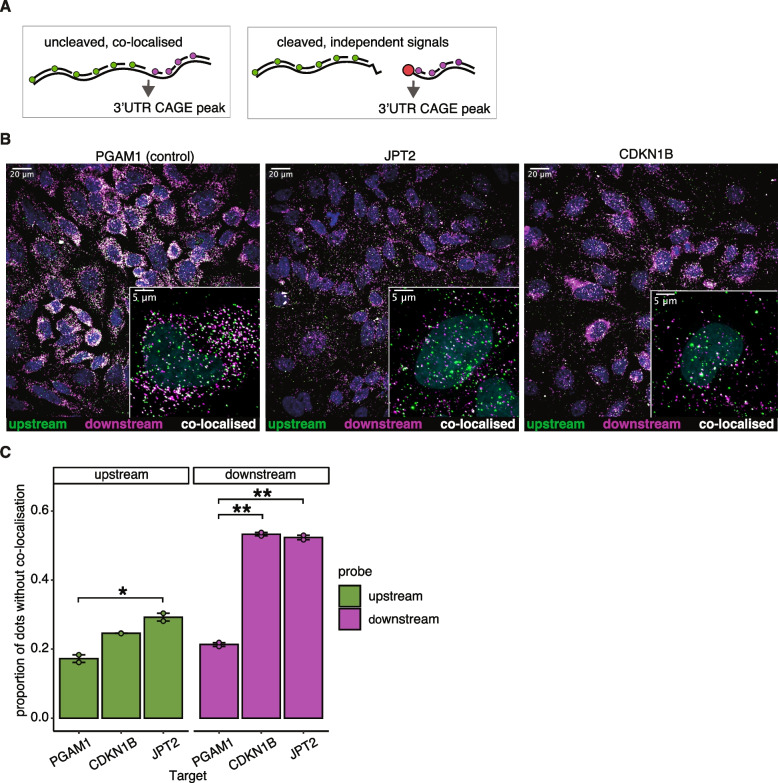
Capped 3′UTR fragments of *CDKN1B* and *JPT2* transcripts do not co-localise with the parental mRNAs. **A** Schematic representation of probe design for HCR-FISH microscopy to separate regions upstream (green) and downstream (purple) of the 3′UTR CAGE sites as cleaved, independent signals and uncleaved, co-localised signals. **B** Representative examples of HCR-FISH images for *PGAM1* (control), *JPT2* and *CDKN1B*. Independent signal from upstream probes is shown in green and signal from downstream probes is shown in purple, with colocalising signals appearing in white. **C** Proportion of independent signals for each upstream or downstream probe. Independent signals are those without a detected colocalising signal from the opposing probset. Error bars represent standard error. Significance was determined using pairwise Welch *t*-tests. *p (adjusted) < 0.05, **p < 0.005

We performed HCR-FISH in HeLa cells to determine whether putative 3′UTR-derived RNAs can be found independently of the RNA upstream of the cleavage site (Fig. 4A, B). In the control transcript, PGAM1, we observed that 17.3% of upstream signals did not have a colocalising downstream signal and 21.3% of downstream signals did not have a colocalising upstream signal (Fig. 4C, Additional file 1: Fig. S4A). However, the mRNAs that contain a 3′UTR CAGE signature were significantly more likely to show independent signals from the RNA downstream of the proposed cleavage site (CDKN1B: 53.3%, *p* adj. < 0.05; JPT2: 52.3%, *p* adj. < 0.05; Fig. 4C). In the case of JPT2, we also observed significantly more independent signals from the upstream probes (29.3%, *p* adj. < 0.05; Fig. 4C). These observations are consistent with the existence of cleaved 3′UTR fragments in the cell, and they reveal that these products may localise differently from their host transcripts.

## Discussion

Previous studies had identified 3′UTR-derived RNAs via enrichment of RNA-seq reads-starts or CAGE signals mapping at 3′UTRs [7, 16, 18]. Nevertheless, most 3′UTR-derived RNAs may have remained undetected until now due to technical limitations inherent to these approaches, including reliance on fragmented-based sequencing methods and potentially biassed library preparations. Here, we provide multiple lines of evidence that complement CAGE and RNA-seq data, including RNA structural features, RBP interactions around 3′UTR CAGE signals and long-read nanopore CAGE to validate the widespread presence of capped 3′UTR-derived RNAs in human cells. We show that capped 3′UTR-derived RNAs are generated post-transcriptionally at positions characterised by the presence of G-rich motifs and specific RBP binding sites. We also demonstrate that, consistent with a functional role, capped 3′UTR-derived RNA sequences are evolutionary conserved and can localise to different subcellular regions than the parental mRNAs.

### The role of AGO2, UPF1 and G-rich motifs in the generation of 3′UTR-derived capped RNAs

One of the key findings of our work is that siRNA action can result in the generation of capped RNAs downstream of the cleavage site (Fig. 3A, C3, Additional file 1: Fig. S3A). However, with the available data we could not quantify the efficiency of such capping or identify all the factors that might be involved in the process. It is well established that AGO2 cleaves the double stranded RNA formed by the reverse complementary binding of siRNAs to their target mRNAs in the cytosol, pointing to a model in which AGO2 cleavage products can be recapped. The strong enrichment of AGO2 to a region with a modest increase in sequence conservation (Additional file 1: Fig. S2D) immediately upstream of the endogenous 3′UTR CAGE peaks (Fig. 3D) suggested that AGO2 also plays a role in the generation of endogenous capped 3′UTR-derived RNAs.

Endogenous RNA interference in mammalian cells is mainly mediated by miRNAs. MiRNAs drive AGO2 to their targets through partial complementarity only and thus, in contrast to siRNAs, do not trigger AGO2 catalytic activity [57]. On the contrary, miRNA action in mammals relies mostly on translational repression and/or exonucleolytic degradation of their targets through the recruitment of other protein partners [57]. This raises two interesting possibilities: (1) that the mechanism by which AGO2 is involved in the generation of endogenous 3′UTR-derived capped RNAs is different to its role in the generation of capped fragments following siRNA action. In this scenario, AGO2 role will likely be independent of its catalytic activity and possibly require the recruitment of other nucleases, or (2) that AGO2 endonucleolytic activity is also important for the generation of endogenous 3′UTR-derived capped RNAs and therefore likely independent of its role in miRNA-mediated silencing of gene expression. The latter model is supported by the finding of a stronger enrichment in AGO2 binding in 3′UTR CAGE peaks in the absence of miRNA binding sites (Additional file 1: Fig. S3F). We also failed to observe CAGE peaks in the vicinity of 29 mRNA targets whose genomics sequences contained miRNA target sites of extensive (< 2 mismatches) complementarity to known miRNAs. Interestingly, we found that AGO2 binds to potential RNA-G4s in 3′UTR CAGE sites (Additional file 1: Fig. S3H–I). Early AGO2-CLIP experiments had already identified an enrichment of a G-rich motif in sequences cross-linked to AGO2 likely in a miRNA-independent manner [55]. AGO2 binding sites neighbouring G-rich sequences may be less likely to be guided by miRNAs, as RNA-G4s can prevent miRNA binding from their target sites [58]. Thus, our analysis suggests that the role of AGO2 in the generation of capped 3′UTR-derived RNAs is independent of its role in miRNA-mediated gene silencing. This is in agreement with previous findings by Andreassi et al. [59]. Moreover, elimination of AGO2 did not affect the enrichment of *JPT2* and *CDKN1B* RT-qPCR signals corresponding to 3′UTR-derived RNAs (S3i) further arguing against a direct role of AGO2 catalytic activity in the generation of these species. In the future, long-read CAGE analysis in models of AGO2 loss of function, possibly in conjunction with the elimination of other AGO proteins (1, 3–4) present in the cells, may help clarify the specific role of AGO2 in the global generation of capped 3′UTR-derived RNAs.

Our work identified UPF1 as the RBP with the strongest binding enrichment around 3′UTR CAGE peaks and uncovered an overlapping of AGO2, UPF1 and G-rich sequences in 3′UTR CAGE sites. Interestingly, a significant overlap between UPF1 and AGO2 binding sites as well as preferential UPF1 binding to structured G-rich regions had been previously reported [60]. Whilst it is well-established that UPF1 plays an essential role in mRNA degradation [38] and binds to GC-rich motifs in 3′UTRs [40], the main trigger of UPF1-mediated mRNA decay remains unknown. It has been suggested that G-enrichment in 3′UTRs plays a vital role in triggering UPF1-mediated mRNA decay [40]. It was therefore tempting to hypothesise a causal role for UPF1 in the generation of 3′UTR-derived RNAs in connection with its role in mRNA decay [18]. Nevertheless, arguing against this possibility, siRNA-mediated downregulation of UPF1 did not change the relative amount of *CDKN1B* and *JPT2* mRNA detected with primers targeting downstream versus upstream of the 3′CAGE peaks (Additional file 1: Fig. S2T). Additional experimental data will be needed to reach a more definitive conclusion, but it is also likely that the presence of RNA-G4 sequences (or strong secondary structures) around 3′UTR CAGE causes the stalling of UPF1 as the helicase translocates in a 5′-3′ direction [61].

Our analysis also shows that the G-rich sequences around 3′UTR CAGE peaks have a strong pairing probability and thus have the potential to form G4 structures (Fig. 2B, Additional file 1: Fig. S2G–L). Nevertheless, although RNA-G4s are known to form stable structures in vitro, recent studies have suggested that they may be less stable in vivo due to active unwinding by RNA helicases [62, 63]. However, Kharel et al. [64] demonstrated that 3′UTR-G4s are dynamically regulated under cellular stress conditions and may play a role in mRNA stability for several transcripts, including the 3′UTR-G4 in the amyloid precursor protein (*APP*) mRNA. The G4 motif in the 3′UTR of *APP* mRNA was found to suppress overproduction of APP protein, but the underlying mechanism remained unclear [65]. Analysis of rG4-seq and CAGE data from HeLa cells showed that the 3′ end of this G4 motif in *APP* 3′UTR precisely coincided with a 3′UTR CAGE signal (Additional file 1: Figs. S1N—APP, S2L). Moreover, long-read sequencing CAGE from cortical neuron samples confirmed that abundant 3′UTR-derived capped RNAs are present in these samples that span from the identified 3′UTR CAGE peak to the end of the annotated *APP* gene (Additional file 1: Fig. S1N—APP). This raises the possibility that the RNA-G4 regulates APP protein through an unknown mechanism that involves the generation of 3′UTR-derived capped RNAs.

Future studies will focus on investigating whether these G-rich sequences form stable G4 structures in other genes and whether they directly contribute to the formation of 3′UTR-derived RNAs. Another important open question that warrants further investigation is whether these G-rich motifs are essential for the recruitment of AGO2 and/or other proteins responsible for the generation of endogenous 3′UTR-derived RNAs.

### Independent localisation of capped 3′UTR fragments from the parental mRNAs

CAGE-seq, RNA-seq, RT-qPCR and long-read CAGE experiments suggested that 3′UTR-derived RNAs in *CDKN1B* and *JPT2* are capped and highly expressed in cells. In line with these experiments, HCR-FISH showed that RNAs derived from *CDKN1B* and *JPT2* 3′UTRs can be detected at separate cytosolic locations than their parental mRNAs. In agreement with the CAGE data, higher ratio of downstream vs. upstream probes is present in *CDKN1B* (Fig. 4A–C, Additional file 1: Fig. S1N—CDKN1B).

Interestingly, some cells exhibited a pronounced perinuclear accumulation of 3′UTR-probes in *CDKN1B* (Fig. 4A), whereas for the majority of the cells, the signal was dispersed throughout the cytosol. An intriguing possibility is a potential cell cycle-dependence, as observed in other aspects of *CDKN1B* gene expression regulation, such as mRNA translation [66, 67]. It has been previously shown that defective *CDKN1* splicing can be rectified to restore CDKN1B/p27^kip^ protein production and induce cell cycle arrest [68, 69]. Likewise, the capped 3′UTR fragments of *CDKN1B* mRNA could regulate p27^kip^ protein in a cell cycle-specific manner, albeit this possibility remains to be investigated. Of note, a separate study proposed a cell-cycle function for 3′UTR fragments of *NURR1* (nuclear receptor related 1 protein) mRNA which were highly expressed in proliferating neuronal cells [17]. Exploring the dynamic nature of these capped 3′UTR fragments and their potential influence on cellular functions in a manner dependent on the cell cycle remains an important area for further investigation.

The different localisation of the 3′UTR-derived fragments to their full-length counterparts raises more questions on the fate and function of these RNAs. It is worth mentioning that analysis of ribosome footprinting data [30] (not shown) failed to reveal any substantial ribosomal binding to capped 3′UTR-derived RNAs. This argues against their translation and supports a role similar to other cytosolic lncRNAs [70], as previously observed by others for specific 3′UTR-derived RNAs [17, 18, 71]. Nevertheless, the binding of the translation initiation factor eIF4E to these RNAs seems paradoxical. Moreover, these findings are in sharp contrast with those by Sudmant et al. who found evidence for ribosomal binding and for the existence of peptides encoded by comparable isolated 3′UTR fragments in human brain samples [17, 18, 71]. It is important to acknowledge that the coverage of ribo-footprinting in 3′UTRs is limited and it therefore cannot fully rule out that some of the capped, eIF4E-bound, 3′UTR-derived RNAs identified in our study are translated. This will require further investigation, possibly in a gene- and tissue-dependent manner.

### Methodological implications

Different abundance and localisation of 3′UTR-derived RNAs relative to their parental transcripts and the 5′ cleavage fragment containing protein coding sequence suggests that 3′UTR fragmented-based sequencing methods might be measuring the wrong RNA species in a significant proportion of cases. Even in the case of RNA-seq, quantitating the signal across the entire length of the uncleaved mRNA might measure a combination of protein-coding and non-coding RNA species. To increase the accuracy of quantitation of protein coding transcript levels, as well as those of 3′UTR-derived RNAs themselves, it may be necessary to develop new computational quantitation methods informed by the results of this paper, which will try to estimate the levels of protein-coding and 3′UTR fragments separately. Moreover, many new drugs which are based on siRNA targeting are already in use or under active clinical trials for treating a variety of conditions including neurological diseases [72]. Side cleavage products of the targeted mRNAs from these therapeutic drugs could be subjected to a cytoplasmic capping mechanism and result in unwanted toxic side effects.

### Limitations

At the moment, we do not have the ability to identify the full-length size of 3′UTR-derived RNAs in a high-throughput manner since the main technique that we used (CAGE-seq) is based on 5′ end sequencing. Also, CAGE-seq method has a limitation on fragment size similar to other HT-sequencing methods with a minimum fragment size of 200 bps. This can be overcome with the long-read CAGE (Additional file 1: Fig. S1N), but the whole data is not yet publicly available. Using only experimental datasets has limitations in coverage, organisms, cell lines and can increase the number of false positives as a result of background noise. It is for example important to note that we have limited our study to RBPs with eCLIP available, which may have prevented the identification of other important proteins involved in the cleavage and/or re-capping of 3′UTR mRNA fragments.

This study is limited to human samples and, even though the sequence conservation suggests that capped 3′UTR-derived fragments may be conserved, this will need further experimental validation. New computational methods will need to be developed for these studies which are currently limited by the availability of such large datasets.

## Conclusions

3′UTR-derived RNAs are emerging as novel regulatory molecules, with potential implications in broad cellular processes such as cell cycle or neuronal homeostasis [17, 18, 71]. However, the molecular mechanisms involved in the generation of these RNA species had been largely unknown. Our study sheds new light into these mechanisms by revealing that capped 3′UTR-derived RNAs originate from sequences rich in G motifs that contain both UPF1 and AGO2 binding sites. These findings suggest a significant role for these elements in the regulatory mechanism. Overall, our findings provide the framework for further investigations where their functions will surely emerge.

## Methods

### HCR-FISH microscopy

HeLa cells (obtained from Cell Services at the Francis Crick Institute) were grown in DMEM supplemented with 10% FBS and plated into 8-well chambered coverslips (Ibidi). Cells were fixed for 10 min at room temperature using 4% paraformaldehyde/0.4% glyoxyl diluted in PBS before permeabilisation overnight at − 20 °C in 70% EtOH. In situ HCR v3.0 with split-initiator probes was performed as described previously [56], except amplification which used 30 nM of each fluorescently labelled hairpin; cells were then stained with1 µg/mL DAPI in 2XSSC before mounting with Fluoromount-G (Thermo Fisher). Cells were imaged on a spinning disk confocal microscope (Nikon CSU-W1 Spinning Disk) using 60 × oil-immersion objective. Six non-overlapping field z-stacks of 17 slices with 0.39 µm z-steps were taken per well. Eight HCR probe pairs per target were designed using the HCR 3.0 Probe Maker [73]. Probes were designed for CDS and 3′UTR to be amplified by the B1 HCR-amplifier with Alexa594 or the B4 HCR-amplifier with Alexa647 (Molecular Technologies), respectively.

### HCR-FISH analysis

We z-projected the images and segmented the nuclei and cytoplasms with Cellpose (v2.0.5, [74]) using the DAPI signal and thresholded AlexaFluor594 signal. We then detected smFISH signal positions using the Fiji plugin RS-FISH (v2.3.0, [75]). We excluded signals that fell outside of a cell mask. For each detected signal, the minimum distance from the centre of the signal to the centre of the nearest signal in the other channel was measured. Co-localisation was defined as a minimum distance of 3 or fewer pixels (with a pixel size of 108 nm) between the centres of the detected signals. Some weak false positive signals are created as a consequence of signal detection, and these do not tend to co-localise between channels. Therefore, we only considered the top half of signals by brightness for a given channel when calculating the proportion of signals that co-localise with a signal in the other channel. The proportion of independent signals (those that do not have a co-localising signal in the other channel) was calculated for both replicates with all 6 fields of view merged for each replicate, and pairwise Welch *t*-tests were calculated in R using the compare_means function from the ggpubr package (https://github.com/kassambara/ggpubr/) with Benjamini–Hochberg correction.

### Cell culture and transfection

K562 and HeLa cells were maintained in RPMI 1640 or DMEM medium, respectively, supplemented with 10% foetal bovine serum (FBS), 1X GlutaMAX (Invitrogen) and 100 U/mL penicillin/streptomycin at 37 °C in 5% CO2 in a humidified incubator.

K562 cells were transfected with 20 µM siRNA targeting *UPF1* or a scramble control (*On target plus*l^R^, Horizon Discovery) using a Neon NxT electroporation system (ThermoFisher Scientific), with Buffer E, 1450 V for 10 mS, as per manufacturer’s instructions. Experiments were performed 48 h after transfection.

### CRISPR-Cas9–mediated deletion of AGO2 in K562 cells

10E5 K562 cells were transiently transfected with 500 ng pSpCas9(BB)-2A-GFP (PX458) (Addgene #48,138) expressing 2 gRNAs targeting exon 2 (AGAGAGAACACCCATTAACG and TGGATGGGGGGCGGCGGCGC) using a Neon NxT electroporation system (ThermoFisher Scientific), as above. Forty-eight hours after transfection, single GFP + and GFP − cells were FACS-sorted in 384-well plates and amplified. Following confirmation of DNA editing by PCR and Sanger sequence in ~ 12 and 4 colonies from those sorted from GFP + and GFP − cells, respectively, 6 and 1, respectively, were submitted to western blot (antibody REF. SAB4200085, Sigma) to confirm AGO2 protein elimination. Three of them were further assessed at the mRNA level as described below.

### Reverse transcription (RT) and quantitative PCR (qPCR)

K562 cells were lysed in Trizol^R^ (Thermo Fisher Scientific) and total RNA extracted as per manufacturer’s instructions. RNA was treated with RQ1 DNase (Promega) in the presence of RNasin ribonuclease inhibitors (Promega). When indicated, treatment of DNAse-treated RNA with TerminatorTM 5′-Phosphate-Dependent Exonuclease (Cambridge Biosciences) was performed in the presence of RNasin ribonuclease inhibitors (Promega) for 60 min at 30 °C as per manufacturer’s instructions. Five hundred nanograms of RNA was reversed transcribed with SuperScript III (Thermo Fisher Scientific) reverse transcriptase, following manufacturer’s instructions in the presence of 1:4 oligod(T):random primers (Thermo Fisher Scientific). Real-time PCR was performed using Fast SYBR™ Green Master Mix (Thermo Fisher Scientific) and specific primers designed within the proximal regions upstream or downstream of the 3′ CAGE signal identified for *CDKN1B* and *JPT2*. The sequences of the primers are as follows:

**Table:** 

Sequence	Target	Location relative to 3′CAGE	Used in figure
TGGAATGGACATCCTGTATAAGCA	CDKN1B (forward)	Upstream (5′C-F)	1, Additional file 1: S1H
AAGCAAATAAGGAAAAACCTAATTGC	CDKN1B (reverse)	Upstream (5′C-R)	1, Additional file 1: S1H
AATCACAAAAATTTGAACACTGG	CDKN1B (forward)	Downstream (3′C-F)	1, Additional file 1: S1H
AAGCCACATGCAGCTATCTAAC	CDKN1B (reverse)	Downstream (3′C-R)	1, Additional file 1: S1H
GCCAGACCAGAAACTCAAGAGA	JPT2 (forward)	Upstream (5′C-F)	1, Additional file 1: S1H
GCCAGGAGACGCTGAGCA	JPT2 (reverse)	Upstream (5′C-R)	1, Additional file 1: S1H
GCCCGCCAGCTGATTG	JPT2 (forward)	Downstream (3′C-F)	1, Additional file 1: S1H
GCGGTTCTGCTAAGAGGTCAA	JPT2 (reverse)	Downstream (5′C-R)	1, Additional file 1: S1H
CAATTAGGTTTTTCCTTATTTGCTTCA	CDKN1B (forward)	Upstream (OC-F)^a^	Additional file 1: S1H
AAATCAAAGCAAGCTCTTCATACCC	CDKN1B (reverse)	Downstream (OC-R)^a^	Additional file 1: S1H
GCTGAGGAACTGACGTGGAG	CDKN1B (forward)	Downstream (3′C-P2-F)	Additional file 1: S1H
ACCCTTCCCCAAAATTGC	CDKN1B (reverse)	Downstream (3′C-P2-R)	Additional file 1: S1H

SLC38A2 3′ primers were obtained from [8]

^a^When used in PCR together, these primers amplify a region spanning the 3′CAGE site, thus are referred to as ‘OC’: Overlapping CAGE

For assessment of UPF1 and AGO2 expression, RT was performed with the High-capacity cDNA Reverse Transcription Kit (ThermoFisher Scientific) following manufacturer’s instructions and qPCR as above. Primer sequences available upon request.

### AGO2-eiCLIP

AGO2-eiCLIP was performed as previously described [50, 76]. In brief, this involved following a previously described non-isotopic iCLIP workflow [77] which had additional modifications to enhance speed and efficiency. This included ligation of a new Cy5.5 labelled adapter (/5Phos/A[NNNNNN]NNNAGATCGGAAGAGCACACG/3Cy55Sp/, where [NNNNNN] indicates a barcode that allows multiplexing of the samples following adaptor ligation) to bound RNA with high concentration T4 RNA ligase (New England Biolabs), use of RecJf exonuclease (New England Biolabs) to remove un-ligated adapter prior to SDS-PAGE analysis, reverse transcription with a biotinylated primer (/5BiotinTEG/CGTGTGCTCTTCCGA), exonuclease III (New England Biolabs) mediated removal of unextended RT-primer, cDNA capture with MyOne C1 streptavidin beads (ThermoFisher Scientific), 3′ adapter (/5Phos/ANNNNNNNAGATCGGAAGAGCGTCGTG/3ddC/) ligation instead of intramolecular ligation, and cDNA elution with nuclease and cation free water at high temperature. For a pellet of cells obtained from a 80% confluent 150-mm dish, we used 100 µl Dynabeads Protein G (Thermo Fisher Scientific) conjugated to 1.5 µg anti-AGO2 antibody (MAB253, Sigma-Aldrich/ Merck). Samples of two biological replicates were sequenced with paired-end reads using NextSeq500.

### Mapping and processing of AGO2-eiCLIP

Pre-processing, mapping to hg38 gene annotation and removal of PCR duplicates of AGO2-eiCLIP data (Additional file 2: Table S1—E-MTAB-12945) and peak calling was performed by using flow.bio (https://app.flow.bio/) database and analysis platform with default settings. Processed data was downloaded from the Flow in BEDgraph format where each count represents crosslinking position and was used for further analysis.

### miRNA analyses

For the genomic separations of crosslink positions, we used GENCODE (v27 primary assembly) annotation and for the separation of transcripts with high and low miRNA targeting in HeLa cells we used [78] annotation. miRNA seed sequences were downloaded from ‘TargetScan’ (www.targetscan.org) database. Only miRNAs expressed in HeLa were selected from miRNA expression profile study [79] with the threshold of more than 10 reads in at least 2 replicates. The miRNA seed sequence heatmap was plotted by counting the expressed seed sequence motifs relative to the AGO2-eiCLIP dominant crosslink sites using the ‘ggplot2’ Bioconductor R package.

### CAGE data pre-processing

Paired-end sequenced CAGE data was downloaded from K562 (ENCSR000CJN) and HeLa (ENCSR000CJJ) cells was downloaded from ENCODE consortium with two biological replicates per sample. FASTQ files were mapped to the hg38 (GENCODE GRCh38.p10) gene annotation using STAR alignment tools (version 2.5.3a) by disabling 5′ read trimming function with the following command:

- * STAR –runMode alignReads –runThreadN $thread –genomeDir $genome_dir –readFilesIn ${path}${data1} ${path}${data2} –outSAMunmapped Within –outFilterMultimapNmax 1 –outFilterMultimapScoreRange 1 –outFileNamePrefix $path$data-STAR-Extend5pOfRead1/ –outSAMtype BAM SortedByCoordinate –outFilterType BySJout –outReadsUnmapped Fastx –outFilterScoreMin 10 –outSAMattrRGline ID:foo –alignEndsType Extend5pOfRead1 –clip5pNbases 9*

### CAGE quality control

For the quality control, we used Bioconductor CAGEr package (v2.0.2) by importing BAM files of mapped reads into R. The pre-processing was done by standardised pipeline provided by ENCODE, where trimming and adapter removal from raw reads was done by cutadapt (v4.2) tool followed by bowtie2 (v2.5.0) alignment tools. This type of mapping was needed to avoid junction reads, which are known to cause issues in certain R packages. Quality controls were then plotted with the following CAGEr functions plotCorrelation2 and plotReverseCumulatives.

### CAGE data processing

The BAM files of mapped reads were converted into BED format using the bamtobed function from bedtools package (version v2.30.0). Each 5′ read position was then used for further analyses. The CAGE peaks were processed by using the *Paraclu* clustering tool (https://gitlab.com/mcfrith/paraclu). Default settings of minimum 5 reads filter for merged replicates were used followed by paraclu.cut.sh which removes:

1. Remove single-position clusters.
2. Remove clusters longer than 200 (Length = column_4 − column_3).
3. Remove clusters with (maximum density/baseline density) < 2.
4. Remove any cluster that is contained in a larger cluster.
5. Single nucleotide clusters were added additionally.

For each cluster, the highest peak of 5′ CAGE reads was used as the max peak position.

### CAGE reproducibility of 3′UTR peaks

Mapped BAM samples from HeLa and K562 cell lines (Additional file 2: Table S1—ENCSR000CJN and ENCSR000CJJ) were converted to BED file format by using *bedtools* bamtobed conversion (v2.30.0) where 5′ read positions were used for further analyses. From each sample, both replicates of 5′ read positions were used to define clusters within the 20-bp window by using bedtools (command: bedtools merge -s -d 20). For each cluster, a maximum number of 5′ read-ends was defined as peak, with a threshold of minimum 2 reads per replicate. Read counts were then normalised by the library size factor function using Bioconductor DESeq2 R package. Correlation plots were then made with R (version 4.1.2) using Bioconductor ggplot2 package for scatter plots.

### eCLIP enrichment relative to 3′UTR CAGE peaks

ENCODE eCLIP data (Additional file 2: Table S1—all eCLIP samples) was processed by following a standardised guideline to study RBP-RNA interactions with CLIP Technologies [32]. We mapped paired-end eCLIP samples to the human hg38 genome using annotation version GRCh38.p7 using the STAR (version 2.5.3a) alignment tool. For adapter removal, the cutadapt tool (version 3.5) was used following the ENCODE guideline with two rounds of adapter removal in case there were double ligated adapters present. After mapping, we removed PCR duplicates using the python script ‘barcode_collapse_pe.py’ provided by ENCODE. For the data format conversions between SAM, BAM and BED file types, we used samtools (version 1.13) and bedtools (version v2.30.0).

For the eiCLIP-AGO2 samples, we used a similar pipeline without double ligation removal and additional custom script to swap random barcodes from the first 7 bps of the read sequence line to the header of the FASTQ read sequence. Uniquely mapped reads with the same genomic positions and non-unique barcode were treated as PCR duplicates by being discarded from the further analyses.

To identify RBP binding enrichments, we first analysed input controls by using eCLIP mock samples from all 80 RBPs from K562 experiments provided by ENCODE consortium. For each sample, we used False Discovery Rate peak finding algorithm from iCount (https://github.com/tomazc/iCount), by assessing the enrichment of crosslink sites at specific binding sites compared to shuffled data. The peak caller was set to 3 nt peak window size to define binding regions genome-wide. Next, we merged all binding regions into one track and longer regions from 50 nts were evenly split into smaller clusters. For each binding site, RBP ratio was calculated relative to the maximum RBP enrichment. These ratios were then used to calculate the RBP enrichment profile around the 3′UTR CAGE peaks.

### RNA-seq

Raw reads of two biological replicates of stranded paired-end RNA-seq samples were downloaded from K562 and HeLa cell lines (Additional file 2: Table S1—ENCFF044SJL, ENCFF728JKQ, GSE99169). FASTQ files were then aligned to the human genome by STAR (version 2.5.3a) alignment tool using GENCODE annotation version GRCh38.p7. Soft-clipping was disabled to contain full length reads by using the following parameters:

- *STAR --runMode alignReads --runThreadN $thread --genomeDir $genome_dir --readFilesIn ${FASTQ.read1} ${FASTQ.read2} --outSAMunmapped Within --outFilterMultimapNmax 1 --outFilterMultimapScoreRange 1 --outFileNamePrefix $path$data1-STAR/ --outSAMtype BAM SortedByCoordinate --outFilterType BySJout --outReadsUnmapped Fastx --outFilterScoreMin 10 --outSAMattrRGline ID:foo --alignEndsType EndToEnd*

Mapped paired-end reads were then converted from BAM to BED by using ‘bedtools bamtobed’ (version v2.30.0) function to extract both sides of each read. Read starts and read ends were then plotted as a metaplot relative to the 3′UTR CAGE peaks.

### CBP20-iCLIP

The CBP20-iCLIP data was downloaded from GEO (Additional file 2: Table S1—GSE94427) and analysed using standard iCLIP processing pipeline where each read was treated as truncated read to identify crosslinking positions of protein-RNA interactions [80]. For the adapter removal, we used the cutadapt tool (version 3.5) with removal of shorter reads than 18 bps.

- cutadapt --match-read-wildcards --times 1 -e 0.1 -O 1 --quality-cutoff 6 -m 18 -a AGATCGGAAG $data > ${data}.adapterTrim.fastq 2> $path$data.adapterTrim.metrics

Random barcode from each read was then removed into a read header by using a custom python script. For mapping the read to human hg38 (GENCODE GRCh38.p7 annotation) genome, we used STAR alignment tool (version 2.5.3a) with the following command:

- *STAR --runMode alignReads --runThreadN $thread --genomeDir $genome_dir --readFilesIn ${data}.adapterTrim.barcodes.fastq --outSAMunmapped Within --outFilterMultimapNmax 1 --outFilterMultimapScoreRange 1 --outFileNamePrefix $data-STAR/ --outSAMattributes All --outStd BAM_SortedByCoordinate --outFilterType BySJout --outReadsUnmapped Fastx --outFilterScoreMin 10 --outSAMattrRGline ID:foo --alignEndsType EndToEnd*

BAM file of mapped reads was then converted into BED file using bedtools (version v2.30.0) bamtobed function followed by removal of PCR duplicates by collapsing identical reads with the same random barcode. For each read, the read start position was used as the crosslinking position and was used for further analysis.

### rG4-seq

The processed RNA-G-quadruplex sequencing (rG4-seq) data from HeLa cells was downloaded from the genomics data repository (Additional file 2: Table S1—GSE77282). The rG4-seq hits were then lifted from the hg19 to hg38 genome using UCSC liftOver webtool. For Fig. 2E, we used middle positions of each rG4-seq target normalised by the number of CAGE (HeLa) peaks from each transcriptome region.

### RNA-maps of iCLIP, eCLIP, eiCLIP and RNA-seq reads start/ends

For the visualisation of all the CLIP based and RNA-seq methods, we used previously developed RNA-map approach [32, 80] with small addition for RNA-seq read end positions by summarising the read start positions relative to the CAGE peaks, TSSs and G-quadruplexes.

### Secondary structure

For each dominant CAGE peak, we extracted a flanking region of 75 bps of the genomic sequence as an input to the RNAfold vienna package (version 2.4.17) with default settings. Each double stranded position was then plotted as a sum of all pairings in the region.

### Predictions of G-quadruplexes

To predict G-quadruplexes in the K562 and HeLa cell line, we first selected CAGE peaks with a threshold of minimum 10 reads per peak in the region of 50 bps upstream and downstream from the peak. For the predictions, we used sequence based prediction tool G4Hunter (https://github.com/AnimaTardeb/G4Hunter) [36] with the following settings: G4Hunter.py -i INPUT.fasta_sequence -o G4Hunter -w 25 -s 1.2.

### Motif discovery

For AGO2 binding motif discovery, we used HOMER software for motif discovery and next-gen sequencing analysis (version 4.9), with default parameters for human genome hg38 and using a 15-bp window around crosslink positions of processed AGO2-eiCLIP-HeLa samples.

### Motif enrichment

For canonical polyA A[A/U]UAAA hexamers enrichment, we first selected 3′UTR ending positions from GENCODE (v27) annotation. For each 3′UTR ending position, we looked at the 100-nt flanking position and counted hexamer coverage per nucleotide. The same was done for 3′UTR CAGE (K562) peaks with removal of CAGE peaks that were in 200 nts into the 3′UTR region CAGE peaks were removed.

### siRNA-mediated knockdown CAGE samples

For the capping of siRNA-targeting sites analyses, we used 28 siRNA-mediated knockdown (KD) samples and 5 control samples with 3 replicates per sample from FANTOM5 [47]. For individual knockdowns, we plotted CAGE transcription start sites (CTSS) of control and knockdowns around the siRNA targeting regions. For the heatmap, we first selected 20 out of 28 samples that had at least two overlapping replicates in the corresponding siRNA targeting region and then merged the replicates. Each siRNA targeting position was manually identified by using BLAST. For the control, we merged together all 5 samples (with 3 replicates per sample) into a metaplot (Additional file 1: Fig. S4A) normalised by the number of samples.

### Conservation plots

Conservation score tracks were obtained from UCSC (pastCons30way annotated to hg38). Inner 3′UTR CAGE peaks were selected, defined as those located ≥ 150 bps downstream from the coding sequence (CDS) and ≥ 150 bps upstream from transcript termination. Randomised control positions within a 150-nt window around each inner 3′UTR CAGE peak were chosen from both K562 and HeLa CAGE peaks. For visualisation, we used *deeptools* (version 3.5.4) profile function.

### Upset plots

Upset plots were generated using the ‘UpSetR’ R package. The intersection of RBP binding sites, G4-seq sites and CAGE peaks was determined using the bedtools (version 2.31.0) intersect function.

### miRNA predicted sites

The predicted miRNA sites were downloaded from targetScanHuman (release 7.2, March 2018).

### GRO-cap seq

Processed GRO-cap seq data (Additional file 2: Table S1—ENCSR626ZNW) in bigWig format was downloaded from ENCODE consortium. For visualisation, deeptools (version 3.5.4) was utilised, using CAGE peaks as target sites within each transcript region.

### Long-read CAGE

Long-read CAGE (Additional file 2: Table S1—E-MTAB-14500) was based on the Cap-Trapper method with the full-length cDNA sequencing using ONT MinION sequencer. After RNA extraction, 10 µg total RNAs from human i^3^N-iPSC that harbours a doxycycline-inducible mouse Ngn2 transgene at an adeno-associated virus integration site 1 (AAVS1) safe-harbour locus of WTC11 iPSC line (https://www.ncbi.nlm.nih.gov/pmc/articles/PMC5639430/), differentiated neural stem cells and differentiated cortical neuron cells were polyadenylated with E-coli poly(A) Polymerase (PAP) (NEB M0276) at 37 °C for 15 min and purified with AMPure RNA Clean XP beads. The PAP treated RNA (5 µg) was reverse transcribed with oligodT_16VN_UMI25_primer (GAGATGTCTCGTGGGCTCGGNNNNNNNNNNNNNNNNNNNNNNNNNCTACGTTTTTTTTTTTTTTTTVN) and Prime Script II Reverse Transcriptase (Takara Bio) at 42 °C for 60 min. After purification with RNAClean XP beads, cap-trapping from the RNA/cDNA hybrid was performed as previously described [19]. RNA from the hybrid was depleted by RNase H (Takara Bio) digestion at 37 °C for 30 min and the product was purified with AMPureXP beads. Then, 5′ linker (constituted of N6 up GTGGTATCAACGCAGAGTACNNNNNN-Phos, GN5 up GTGGTATCAACGCAGAGTACGNNNNN-Phos, down Phos-GTACTCTGCGTTGATACCAC-Phos) was ligated to the cDNA with Mighty Mix (Takara Bio) with overnight incubation and the ligated cDNA was purified with AMPure XP beads. Shrimp Alkaline Phosphatase (Takara Bio) was used to remove phosphates from the ligated linker and the product was purified with AMPureXP beads. The 5′ linker ligated cDNA was then second strand synthesised with KAPA HiFi mix (Roche) and the 2nd synthesis primer_UMI15 at 95 °C for 5 min, 55 °C for 5 min and 72 °C for 30 min. Exonuclease I (Takara Bio) was added and incubated at 37 °C for 30 min to remove excessive primer. Then, the cDNA/DNA hybrid was purified with AMPureXP and amplified with PrimerSTAR GXL DNA polymerase (Takara Bio) using PCR primers (fwd_CTACACTCGTCGGCAGCGTC, rev _GAGATGTCTCGTGGGCTCGG) for 7 cycles. The library was then subjected to the SQK-LSK110 (Oxford Nanopore Technologies) protocol according to manufacturer’s instructions and sequenced with R9.4 flowcell (FLO-MIN106) in MinION sequencer. Basecalling was processed by Guppy v5.0.14 basecaller software provided by Oxford Nanopore Technologies in high-accuracy mode to generate FASTQ files from FAST5 files. To prepare clean reads from FASTQ files, adapter sequences (VNP_GAGATGTCTCGTGGGCTCGGNNNNNNNNNNNNNNNCTACG and SSP_ CTACACTCGTCGGCAGCGTCNNNNNNNNNNNNNNNNNNNNNNNNNGTGGTATCAACGCAGAGTAC) and poly-A tails were trimmed by primer-chop (https://gitlab.com/mcfrith/primer-chop) and then oriented to original RNA strand. The clean FASTQ reads were mapped on our target genes.

To visualise the long-read CAGE, we grouped and coloured reads according to the location of the read start: near the canonical TSS, in the 3′ UTR and/or near the 3′UTR CAGE signal, or other initiation site. These BAM files were visualised in IGV. As a complementary approach, for the CCN1 and CDKN1B, we used the Nanoblot package v1.1 with DESeq2 normalisation [81].

## Supplementary Information

Additional file 1: **Figures S1**, **S2**, **S3**, **S4**. Each supplementary figure corresponds to the main Figs. in the manuscript, in the same order. **Fig. S1** Related to Fig. 1. **A** Pearson’s correlation of raw CAGE read counts per TSS or consensus cluster across biological replicates and cell types. **B** Reverse cumulative distribution of CAGE reads after normalisation using CAGEr package [82]. **C** Total number of CAGE reads in each sample. **D** Density of total 5′ CAGE read positions normalised by the length of the correspondent transcript region identified in CAGE-seq libraries of K562 and HeLa samples with two biological replicates each, provided by ENCODE. **E** Percentage of CAGE tags per transcript region using random primers, Oligo(dT) primers, and combination of both primers (1:4 Oligo(dT):Random primers) in CAGE-seq libraries of THP-1 cells generated by RIKEN. **F-I** Pearson’s correlation between CAGE-seq replicates and different cell lines samples in 3′UTRs, 5′UTRs, CDS and introns. **J** Top: Plot of the normalised coverage of the 5′ ends of forward paired-end reads (yellow line) and 3′ ends of reverse paired-end reads (blue line) of RNA-seq relative to 3′UTR CAGE peaks in HeLa cells. Bottom: Schematic representation of paired-end read positioning. Forward and reversed paired-end reads are presented in yellow and blue, respectively. The black box represents the ends of reads that are plotted in the top graph. **K** RT-qPCR data of gene expression using primers designed to amplify sequences located downstream (3'C), upstream (5'C) and overlapping (AC) the 3′UTR CAGE sites of *CDKN1B* and *JPT2*. Data represents fold detection (six biological replicates) using downstream versus upstream/overlapping primers relative to the 3′UTR CAGE peaks. Primer target sequences relative to the 3′UTR CAGE peak are schematically represented on the top right-hand side and visualised for each gene using IGV genome browser on the bottom. Each dot represents the value of an independent biological replicate. **L** Top gene examples with strongest 3′UTR CAGE peaks present in K562 and HeLa cell lines using IGV-genome browser for visualisation. **M** Visualisation of RNA-seq reads relative to dominant 3′UTR CAGE peaks in *CDKN1B* and *JPT2* gene using IGV-genome browser. **N** Visualisation of 10 gene examples with 3′UTR CAGE peaks, rG4-seq clusters and long-read CAGE reads using IGV-genome browser. **Fig. S2** Related to Fig. 2. **A** RNA-map showing normalised density of CBP20-iCLIP crosslink sites relative to dominant 3′UTR and 5′UTR CAGE peaks. **B** Mean score of GRO-cap seq coverage per CAGE peak for 5’UTR, CDS, intron and 3’UTR regions. The heatmap represents GRO-cap seq scores plotted with deeptools. **C** RNA-map showing normalised density of cap-CLIP crosslink sites relative to dominant 3′UTR and 5′UTR CAGE peaks. **D** Mean coverage of conservation score from UCSC phastCons30way track relative to inner 3′UTR CAGE peaks and randomised control positions around the same region of 150-nt window for each peak. **E** Normalised motif enrichment of canonical PolyA motifs relative to 3′UTR ends and to the dominant 3′UTR CAGE peaks. **F** Sequence logos around K562 cells’ CAGE peaks across different transcript regions. **G** The 75-nt region centred on K562 cells’ CAGE peaks at different transcript regions was used to calculate pairing probability with the RNAfold program, and the average pairing probability of each nucleotide is shown for the 50-nt region around CAGE peaks. **H** GGG-motif enrichment relative to CAGE peaks. **I** GGG-motif enrichment relative to CAGE peaks. **J** Summarised score from G4-Hunter prediction tool [36] in the region of 50 nts upstream and downstream relative to CAGE peaks. **K** Summarised score from G4-Hunter prediction [36] tool in the region of 50 nts upstream and downstream relative to CAGE peaks. **L** Enrichment of RNA-G-quadruplex sequencing hits from HeLa cells relative to CAGE peaks. **M** Percentage of G4-seq sites per transcript region. **N** Enrichment of eCLIP cross-linking clusters surrounding 5′UTR CAGE peaks from 80 different RBP samples in K562 cells from ENCODE database using sum of log ratios. The red line represents the threshold of top 10 RBP targets. **O** Enrichment of eCLIP cross-linking clusters surrounding intronic CAGE peaks from 80 different RBP samples in K562 cells from ENCODE database using sum of log ratios. The red line represents the threshold of top 10 RBP targets. **P** Enrichment of eCLIP cross-linking clusters surrounding CDS CAGE peaks from 80 different RBP samples in K562 cells from ENCODE database using sum of log ratios. The red line represents the threshold of top 10 RBP targets. **Q** Pearson’s correlation between the 3′UTR CAGE tags and RNA-seq read coverage per gene (top-left). Pearson’s correlation between the 3′UTR crosslink coverage of UPF1-eCLIP and 3′UTR CAGE tags (top-right). Pearson’s correlation between the 3′UTR length and 3′UTR crosslink coverage of UPF1-eCLIP (bottom-left). Pearson’s correlation between the 3′UTR length and 3′UTR CAGE tags (bottom-right). **R** UPF1-eCLIP crosslink enrichment relative to the distance from the 3′UTR CAGE peaks. **S** Heatmap of UPF1-eCLIP crosslink site enrichment showing the top 500 3′UTR AGO2 targets in 100-nt flanking region relative to 3′UTR CAGE peaks. The heatmap represents log2 of crosslink counts, normalised by the mean of all counts within 200 nts of the targeting site. **T** K562 cells were transfected with siRNAs targeting UPF1 or non-targeting controls (C). Left-hand side panel: *UPF1* expression was measured with RT-qPCR. Data is normalised by the housekeeping gene RPLP0 and presented as fold change of the control. Right-hand side panel: RT-qPCR data of gene expression using primers designed to amplify sequences located downstream (3'C) and upstream (5'C) the 3′UTR CAGE sites of *CDKN1B* and *JPT2*. Data represents fold detection using downstream (3'C) versus upstream (5'C) primers. Each dot represents an independent biological replicate. Primer target sequences relative to the 3′UTR CAGE peaks are schematically represented on the right. **Fig. S3** Related to Fig. 3. **A** Visualisation of 5′ CAGE reads relative to dominant transcription start site (TSS) and relative to small interfering RNA (siRNA) of ISL1 target (in red) for CAGE-ISL1-KD and CAGE-control samples with 3 biological replicates using IGV-genome browser. **B** Percentage of AGO2-eiCLIP binding sites per transcript region. **C** Binding enrichment of AGO2-eiCLIP relative to miRNA-regulated transcripts and non-miRNA-regulated transcript in HeLa. **D** Heatmap of miRNA-seed sequence enrichment in 30-nt flanking region showing the top 500 AGO2 binding sites relative to AGO-eiCLIP crosslink sites. Metaplot visualises the miRNA-seed sequence composition relative to the 5′ of the AGO2 binding site. **E** Heatmap of AGO2-eiCLIP crosslink site enrichment showing the top 500 3′UTR AGO2 targets in 100-nt flanking region relative to 3′UTR CAGE peaks. The heatmap represents log2 of crosslink counts, normalised by the mean of all counts within 200 nts of the targeting site. **F** RNA-map showing normalised density of AGO2 crosslink sites from AGO2 binding sites that contain (mir+) or are absent (mir-) from predicted miRNA binding sites relative to 3′UTR CAGE peaks. **G** AGO2 mRNA expression was measured with RT-qPCR in 3 clonal subpopulations generated by single cell sorting of K562 cells transfected with a plasmid expressing Cas9 and two gRNAs targeting AGO2 and preselected by genomic DNA sequencing. Both KO1 and KO2 contained edited AGO2 sequences and control wild-type sequences. Data is normalised by the housekeeping gene RPLP0 and presented as a fold change of the control. Each dot represents an independent biological replicate. **H** AGO2 protein was detected by western blot in 6 clonal subpopulations generated as in Fig. S3G. Clon 2 and 6 were selected for further experiments and re-named as KO1 and KO2. This experiment was performed once. **I** RT-qPCR data of gene expression using primers designed to amplify sequences located downstream (3'C) and upstream (5'C) the 3′UTR CAGE sites of CDKN1B and JPT2. Data represents fold detection using downstream (3'C) versus upstream (5'C) primers. Each dot represents an independent biological replicate. **J** Sequence logos and statistics of top 12 significantly enriched motifs of AGO2-eiCLIP binding sites using Homer for de novo motif discovery. **K** Enrichment of AGO2-eiCLIP cross-linking sites relative to the 3′end of the rG4-seq site. **L** Heatmap for AGO2-eiCLIP crosslink site enrichment to show the top 500 3′UTR AGO2 targets in 100-nt flanking region relative to 3′end of rG4-seq site. The heatmap represents log2 of crosslink counts, normalised by the mean of all counts within 200 nts of the targeting site. **M** Enrichment of UPF1-eCLIP cross-linking sites relative to the 3′end of the rG4-seq site. **N** Heatmap for UPF1-eCLIP crosslink site enrichment to show the top 500 3′UTR UPF1 targets in 100-nt flanking region relative to 3′end of rG4-seq site. The heatmap represents log2 of crosslink counts, normalised by the mean of all counts within 200 nts of the targeting site. **O** Upset plot of intersection of AGO2-eiCLIP and UPF1-eCLIP binding sites, G4-seq sites relative to 3′UTR CAGE peaks. **P** RNA-map showing normalised density of AGO2 crosslink sites relative to 3′UTR CAGE peaks intersecting G4-seq site (G4+) or not (G4-) from Fig. S3L. **Q** RNA-map showing normalised density of UPF1 crosslink sites relative to 3′UTR CAGE peaks intersecting G4-seq site (G4+) or not (G4-) from Fig. S3L. **Fig. S4**: Related to Fig. 4. **A** Density plots showing the shortest distance per detected signal in pixels to a signal of the opposite colour. The dashed line shows the cutoff used to distinguish colocalising and non-colocalising signals.

Additional file 2: **Table S1:** Reference list of all datasets used in this study in excel spreadsheet format. 

Additional file 3: **Table S2:** Genomic locations of 32,065 unique 3′UTR CAGE clusters across all CAGE samples from HeLa and K562 cell lines. Each sample contains normalised 5′ read positions for each cluster in text format.

## Data Availability

All data generated or analysed during this study are included in this published article, its supplementary information files and publicly available repositories. Raw and processed sequencing files of eiCLIP-AGO2 experiment and long-read CAGE have been deposited in the ArrayExpress archive accessible at E-MTAB-12944/E-MTAB-12945 and E-MTAB-14500. All the pipelines and scripts used in this study are deposited and available on Zenodo (https://zenodo.org/doi/10.5281/zenodo.13828322; DOI: 10.5281/zenodo.13828323). All other datasets used in this study are provided in Additional file 2: Table S1.
