# Correction: Widespread 3′UTR capped RNAs derive from G-rich regions in proximity to AGO2 binding sites

**DOI:** 10.1186/s12915-026-02580-0

**Published:** 2026-03-30

**Authors:** Nejc Haberman, Holly Digby, Rupert Faraway, Rebecca Cheung, Anob M. Chakrabarti, Andrew M. Jobbins, Callum Parr, Kayoko Yasuzawa, Takeya Kasukawa, Chi Wai Yip, Masaki Kato, Hazuki Takahashi, Piero Carninci, Santiago Vernia, Jernej Ule, Christopher R. Sibley, Aida Martinez-Sanchez, Boris Lenhard

**Affiliations:** 1 MRC Laboratory of Medical Sciences, London, W12 0NN UK; 2 Institute of Clinical Sciences, Faculty of Medicine, Imperial College London, London, W12 0NN UK; 3 Division of Neuroscience, Department of Brain Sciences, Imperial College London, London, W12 0NN UK; 4 UK Dementia Research Institute at King’s College London, London, SE5 9RX UK; 5 The Francis Crick Institute, London, NW1 1AT UK; 6 Section of Cell Biology and Functional Genomics, Department of Metabolism, Digestion and Reproduction, Imperial College London, London, W12 0NN UK; 7 UCL Respiratory, Division of Medicine, University College London, London, WC1E 6JF UK; 8 RIKEN Center for Integrative Medical Sciences, Yokohama, Kanagawa 230-0045 Japan; 9 Human Technopole, Milan, 20157 Italy; 10 Institute of Clinical Sciences, Faculty of Medicine, London, W12 0NN UK; 11 Institute of Biomedicine of Valencia (CSIC), Valencia, 46012 Spain; 12 Institute of Quantitative Biology, Biochemistry and Biotechnology, School of Biological Sciences, University of Edinburgh, Edinburgh, UK

**Correction: BMC Biol 22, 254 (2024)**

**https://doi.org/10.1186/s12915-024-02032-7**

The authors wish to note that the Competing Interests statement in the original article [1], should instead read as follows:

“C.R.S. is inventor on IP filings covering the eiCLIP method. The other authors declare no competing interests.”
