# Supplementary material for: Widespread 3′UTR capped RNAs derive from G-rich regions in proximity to AGO2 binding sites
Source: BMC Biol. 2024 Nov 7;22:254. doi: 10.1186/s12915-024-02032-7 (PMC11546257; doi:10.1186/s12915-024-02032-7)
Supplement: Supplementary file 1 — Additional file 1: Figures S1, S2, S3, S4. Each supplementary figure corresponds to the main Figs. in the manuscript, in the same order. Fig. S1 Related to Fig. 1. A Pearson’s correlation of raw CAGE read counts per TSS or consensus cluster across biological replicates and cell types. B Reverse cumulative distribution of CAGE reads after normalisation using CAGEr package [82]. C Total number of CAGE reads in each sample. D Density of total 5′ CAGE read positions normalised by the length of the correspondent transcript region identified in CAGE-seq libraries of K562 and HeLa samples with two biological replicates each, provided by ENCODE. E Percentage of CAGE tags per transcript region using random primers, Oligo(dT) primers, and combination of both primers (1:4 Oligo(dT):Random primers) in CAGE-seq libraries of THP-1 cells generated by RIKEN. F-I Pearson’s correlation between CAGE-seq replicates and different cell lines samples in 3′UTRs, 5′UTRs, CDS and introns. J Top: Plot of the normalised coverage of the 5′ ends of forward paired-end reads (yellow line) and 3′ ends of reverse paired-end reads (blue line) of RNA-seq relative to 3′UTR CAGE peaks in HeLa cells. Bottom: Schematic representation of paired-end read positioning. Forward and reversed paired-end reads are presented in yellow and blue, respectively. The black box represents the ends of reads that are plotted in the top graph. K RT-qPCR data of gene expression using primers designed to amplify sequences located downstream (3'C), upstream (5'C) and overlapping (AC) the 3′UTR CAGE sites of CDKN1B and JPT2. Data represents fold detection (six biological replicates) using downstream versus upstream/overlapping primers relative to the 3′UTR CAGE peaks. Primer target sequences relative to the 3′UTR CAGE peak are schematically represented on the top right-hand side and visualised for each gene using IGV genome browser on the bottom. Each dot represents the value of an independent biological replicate. L Top gene [file 12915_2024_2032_MOESM1_ESM.zip › Additional_File_1_Figure_S1.pdf]

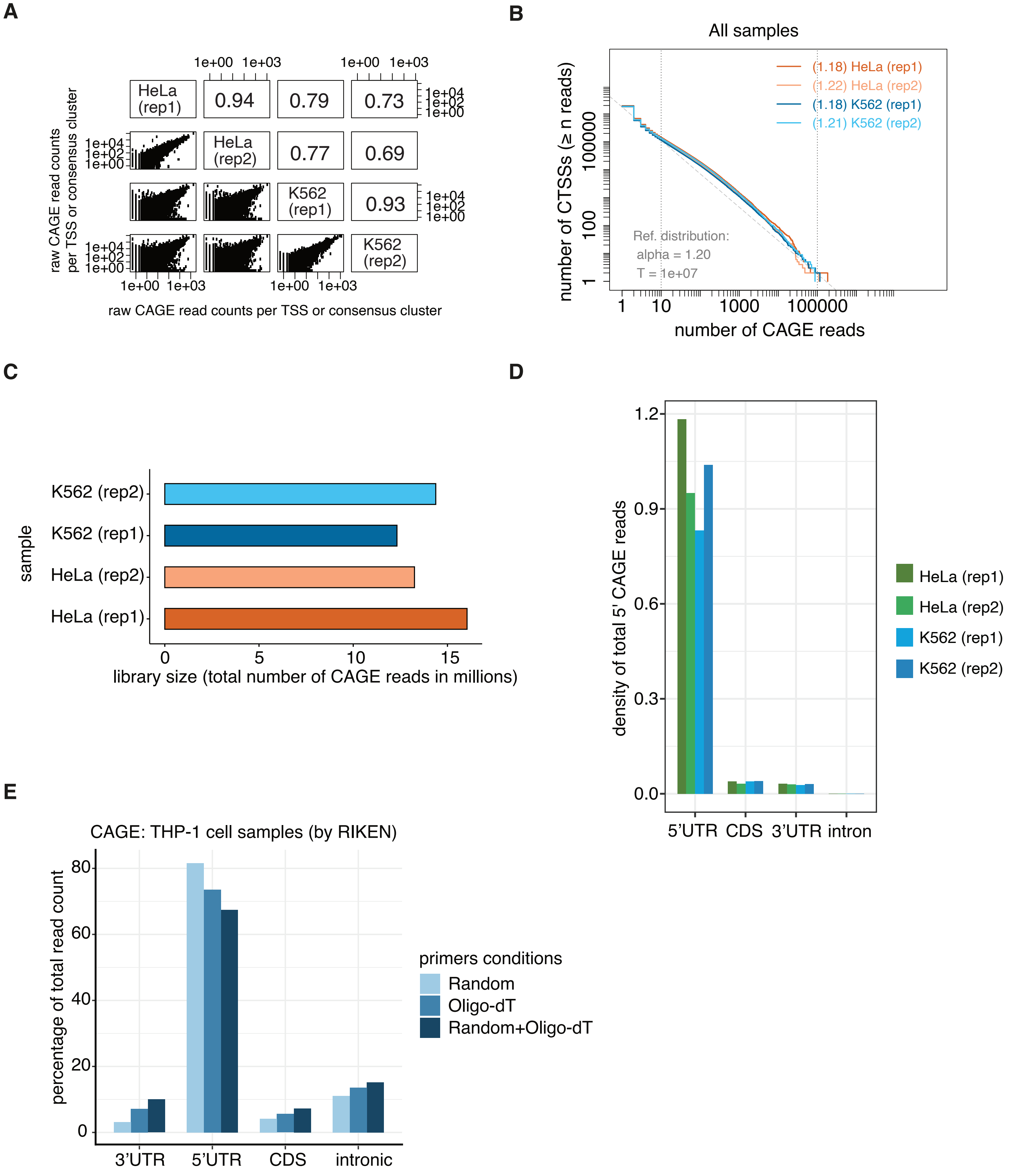
A
HeLa (rep1)
HeLa (rep2)
K562 (rep1)
K562 (rep2)
0.94
0.79
0.73
0.77
0.69
0.93
1e+00
1e+02
1e+03
1e+04
raw CAGE read counts per TSS or consensus cluster
B
All samples
(1.18) HeLa (rep1)
(1.22) HeLa (rep2)
(1.18) K562 (rep1)
(1.21) K562 (rep2)
Ref. distribution:
alpha = 1.20
T = 1e+07
number of CTSSs (≥ n reads)
number of CAGE reads
1
10
100
1000
100000
C
sample
K562 (rep2)
K562 (rep1)
HeLa (rep2)
HeLa (rep1)
0
5
10
15
library size (total number of CAGE reads in millions)
D
density of total 5' CAGE reads
0.0
0.3
0.6
0.9
1.2
5'UTR
CDS
3'UTR
intron
HeLa (rep1)
HeLa (rep2)
K562 (rep1)
K562 (rep2)
E
CAGE: THP-1 cell samples (by RIKEN)
percentage of total read count
0
20
40
60
80
3'UTR
5'UTR
CDS
intronic
primers conditions
Random
Oligo-dT
Random+Oligo-dT

F

3'UTR

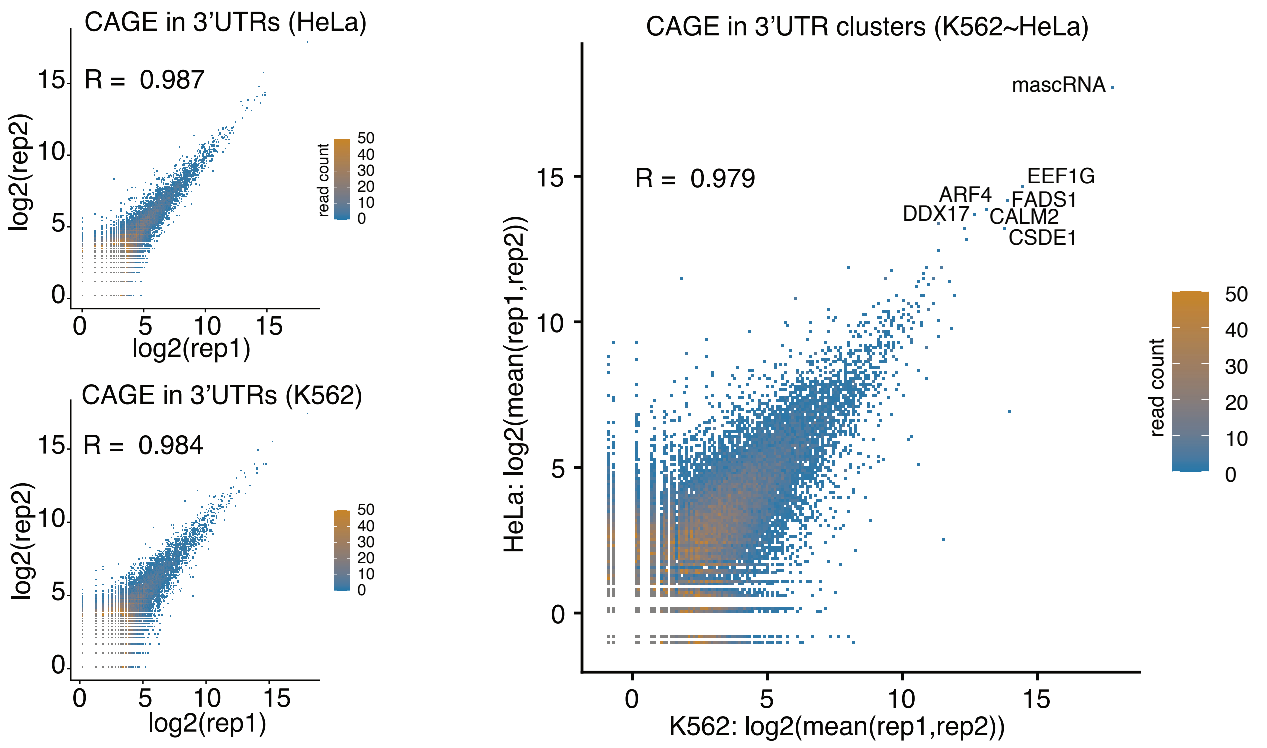
CAGE in 3'UTRs (HeLa)
R = 0.987
log2(rep2)
log2(rep1)
read count
CAGE in 3'UTRs (K562)
R = 0.984
log2(rep2)
log2(rep1)
read count
CAGE in 3'UTR clusters (K562~HeLa)
R = 0.979
mascRNA
EEF1G
ARF4
FADS1
DDX17
CALM2
CSDE1
HeLa: log2(mean(rep1,rep2))
K562: log2(mean(rep1,rep2))
read count

G

5'UTR

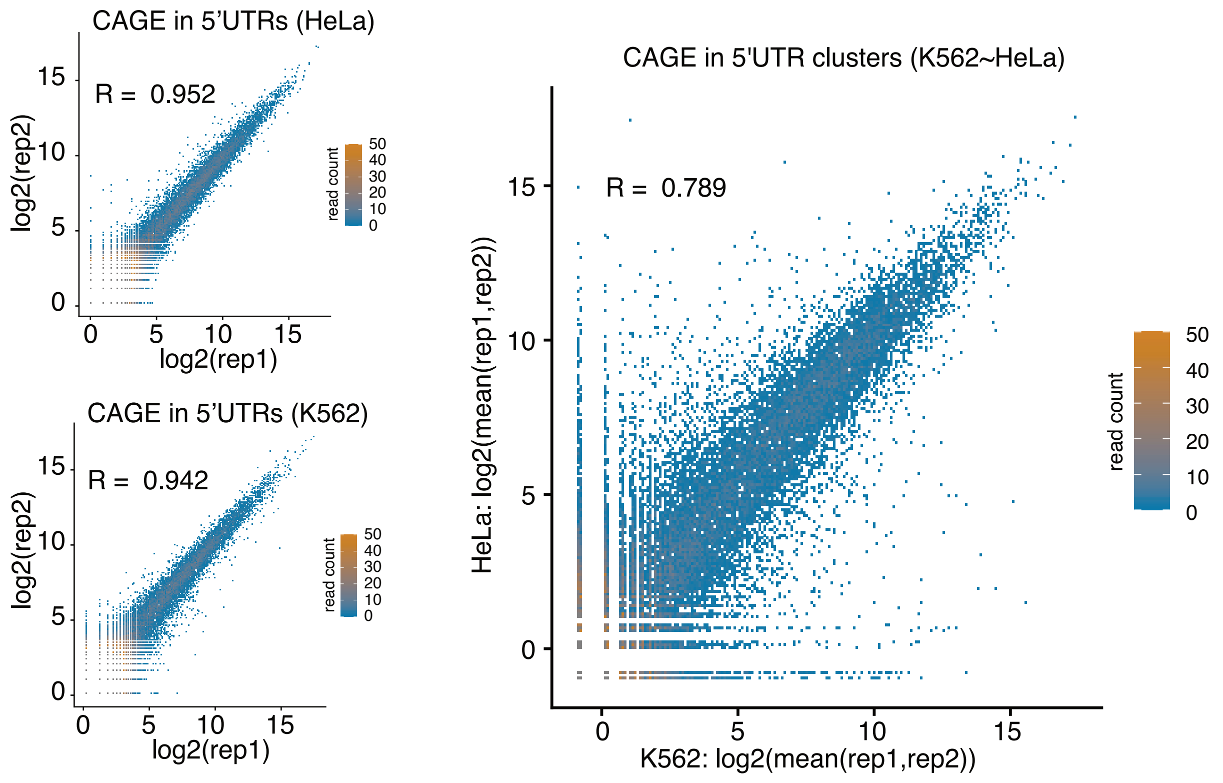
CAGE in 5'UTRs (HeLa)
R = 0.952
log2(rep2)
log2(rep1)
read count
CAGE in 5'UTRs (K562)
R = 0.942
log2(rep2)
log2(rep1)
read count
CAGE in 5'UTR clusters (K562~HeLa)
R = 0.789
HeLa: log2(mean(rep1,rep2))
K562: log2(mean(rep1,rep2))
read count

H

CDS

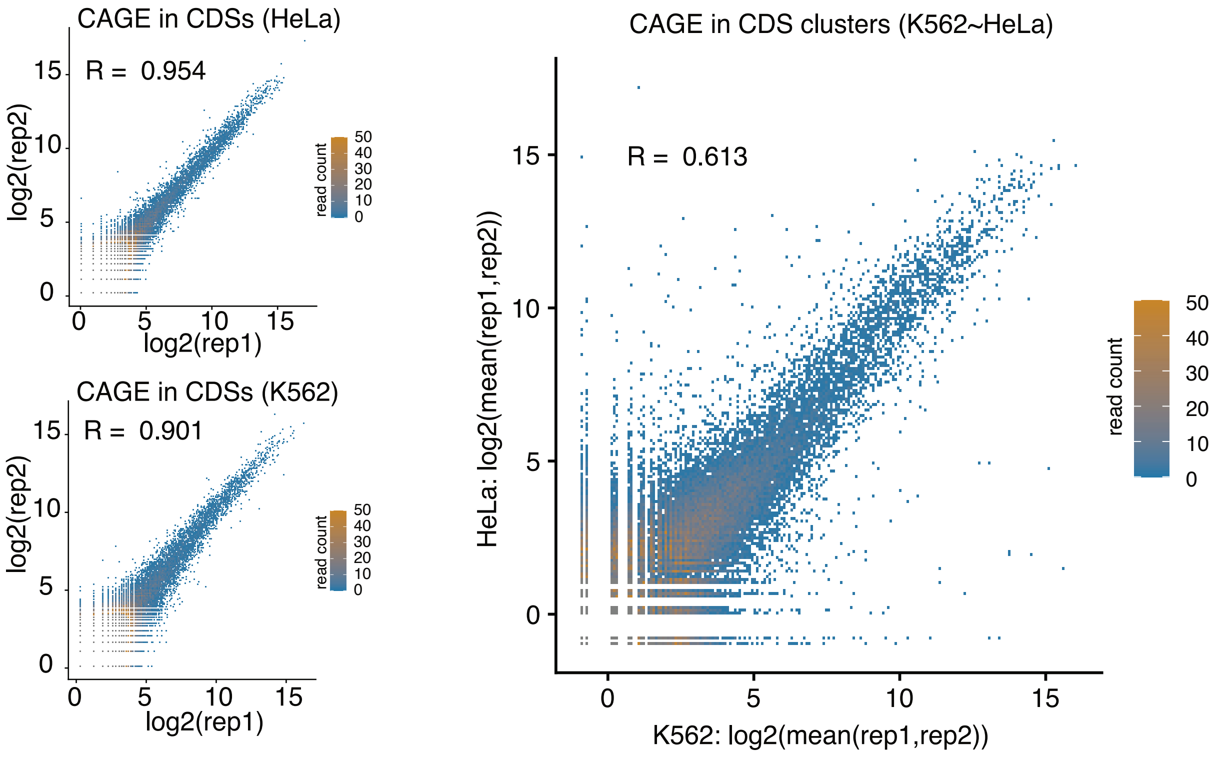

I

intron

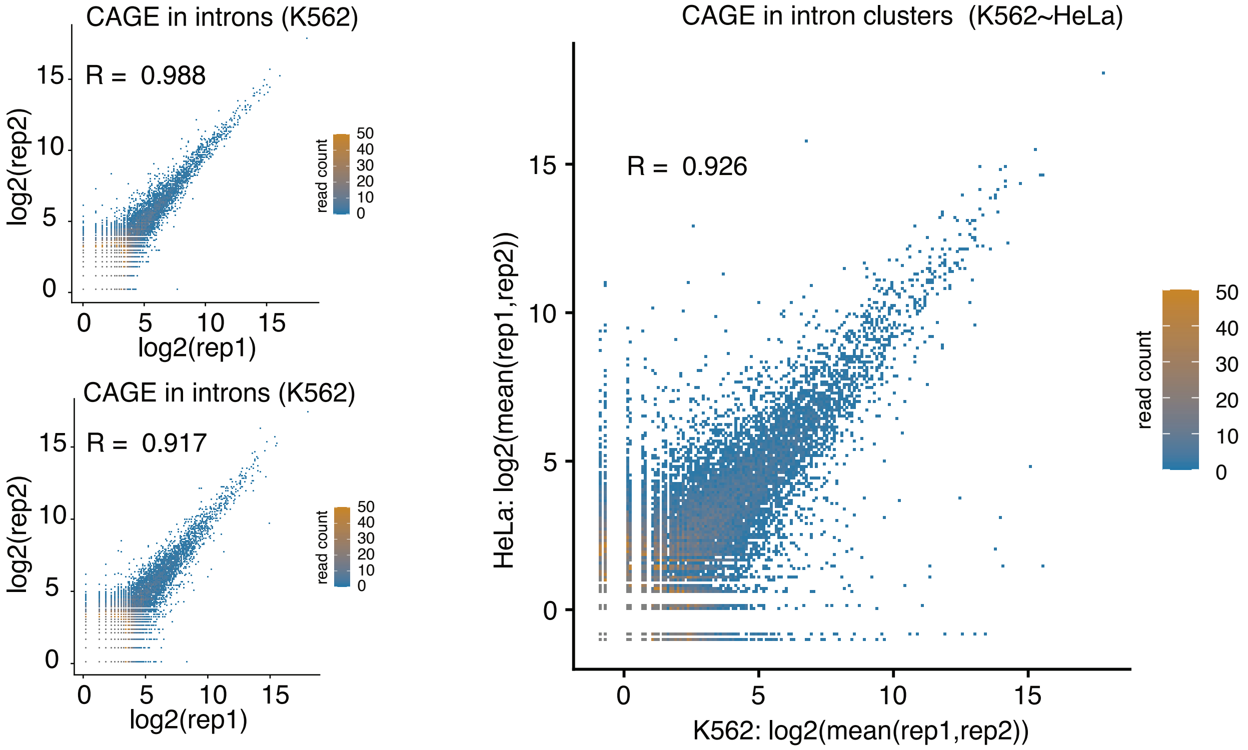

J

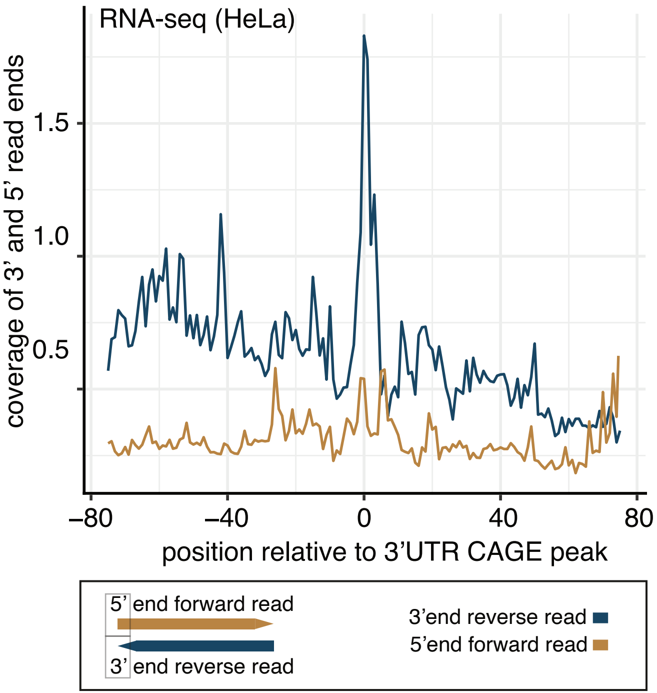
RNA-seq (HeLa)
coverage of 3’ and 5’ read ends
1.5
1.0
0.5
–80
–40
0
40
80
position relative to 3’UTR CAGE peak
5’ end forward read
3’ end reverse read
3’end reverse read
5’end forward read

K

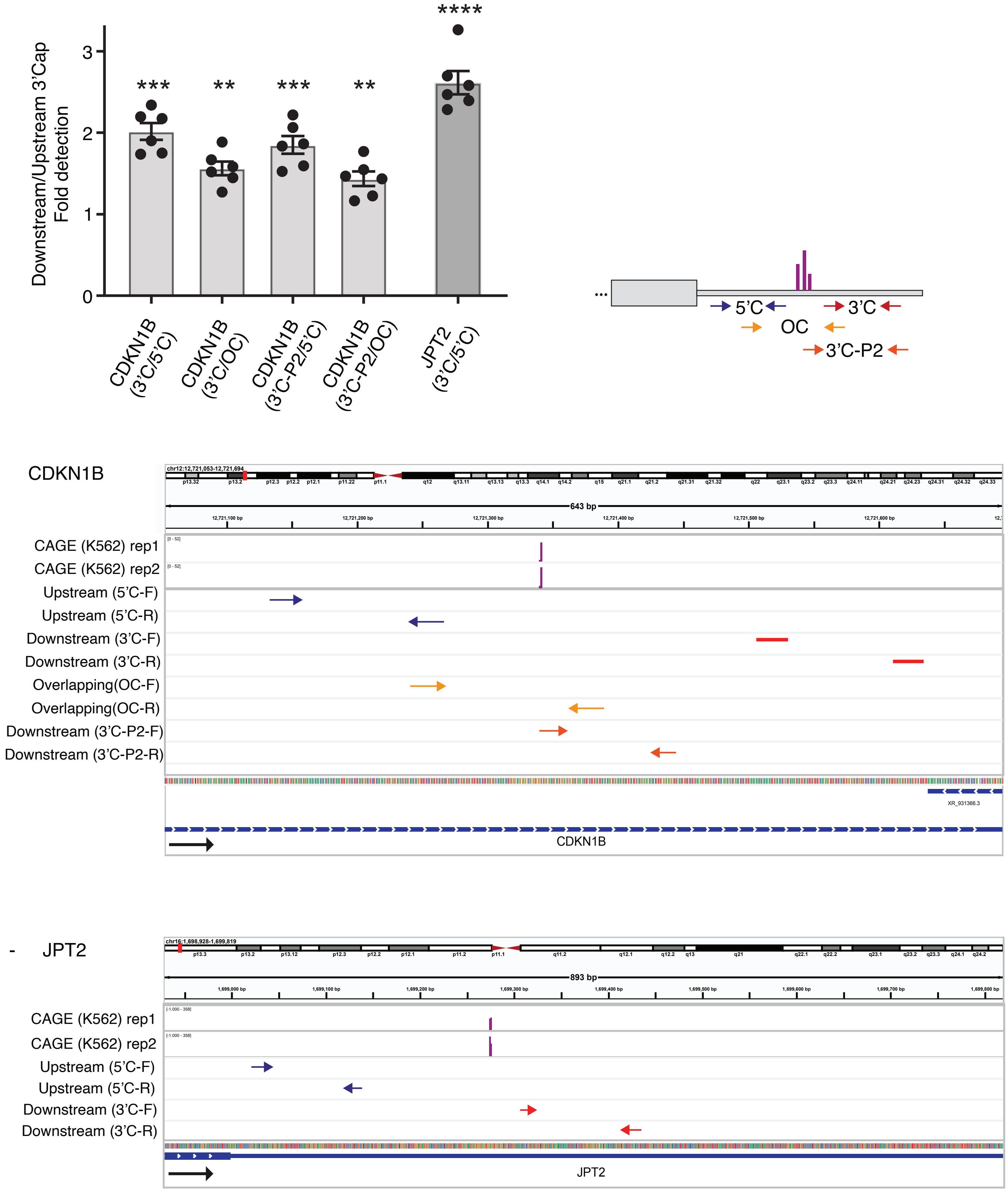

Downstream/Upstream 3'Cap
Fold detection
0
1
2
3
***
**
***
**
****
CDKN1B (3'C/5'C)
CDKN1B (3'C/OC)
CDKN1B (3'C-P2/5'C)
CDKN1B (3'C-P2/OC)
JPT2 (3'C/5'C)
...
5'C
3'C
OC
3'C-P2
- CDKN1B
chr12:12,721,053-12,721,694
643 bp
CAGE (K562) rep1
CAGE (K562) rep2
Upstream (5'C-F)
Upstream (5'C-R)
Downstream (3'C-F)
Downstream (3'C-R)
Overlapping(OC-F)
Overlapping(OC-R)
Downstream (3'C-P2-F)
Downstream (3'C-P2-R)
XR_931366.3
CDKN1B
- JPT2
chr16:1,698,928-1,699,819
893 bp
CAGE (K562) rep1
CAGE (K562) rep2
Upstream (5'C-F)
Upstream (5'C-R)
Downstream (3'C-F)
Downstream (3'C-R)
JPT2

L

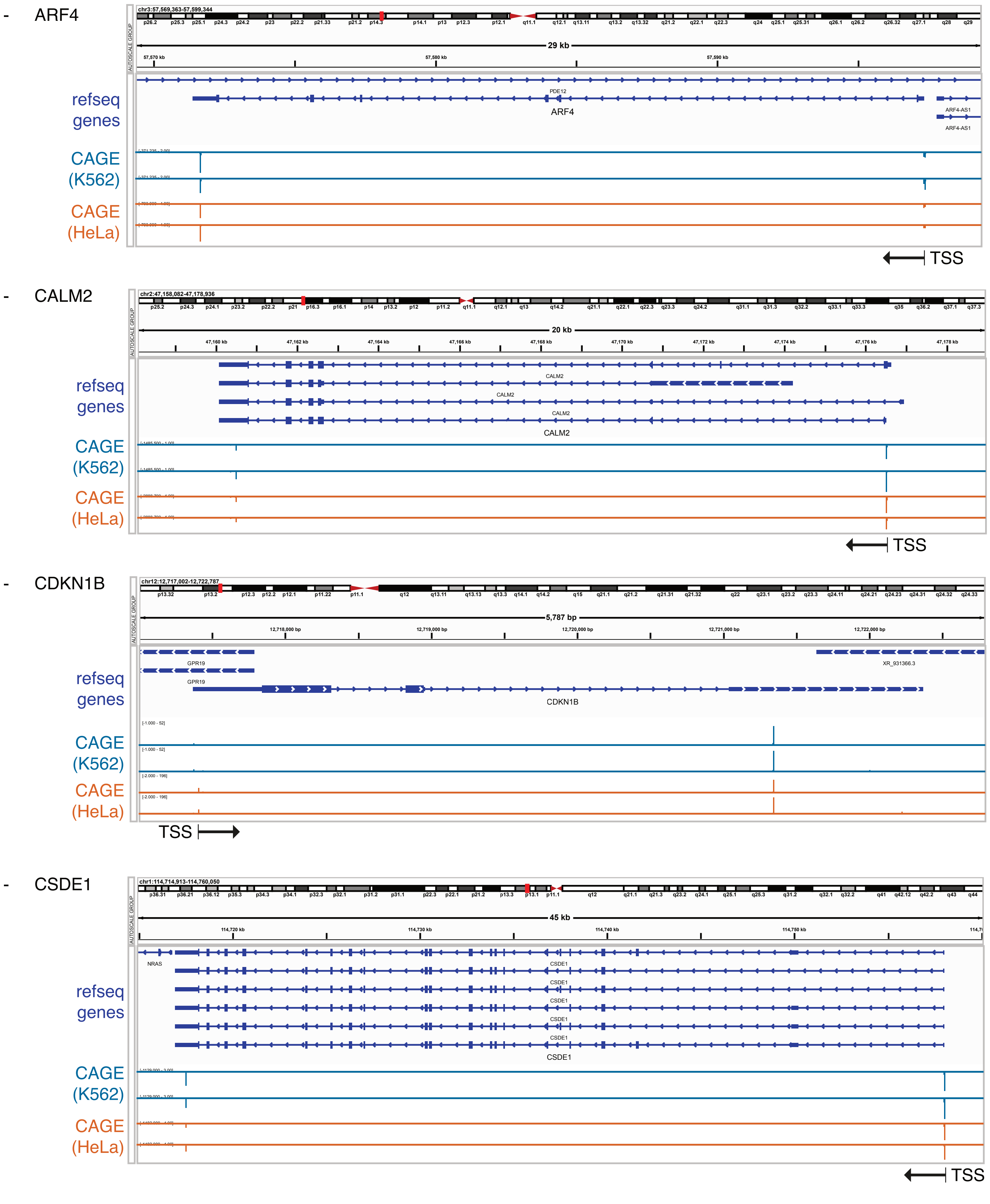

- ARF4
chr3:57,569,363-57,599,344
29 kb
57,570 kb
57,580 kb
57,590 kb
PDE12
ARF4
ARF4-AS1
ARF4-AS1
refseq genes
CAGE (K562)
CAGE (HeLa)
TSS
- CALM2
chr2:47,158,082-47,178,936
20 kb
CALM2
CALM2
CALM2
CALM2
refseq genes
CAGE (K562)
CAGE (HeLa)
TSS
- CDKN1B
chr12:12,717,002-12,722,787
5,787 bp
12,718,000 bp
12,719,000 bp
12,720,000 bp
12,721,000 bp
12,722,000 bp
GPR19
GPR19
XR_931366.3
CDKN1B
refseq genes
CAGE (K562)
CAGE (HeLa)
TSS
- CSDE1
chr1:114,714,913-114,760,050
45 kb
114,720 kb
114,730 kb
114,740 kb
114,750 kb
NRAS
CSDE1
CSDE1
CSDE1
CSDE1
CSDE1
CSDE1
refseq genes
CAGE (K562)
CAGE (HeLa)
TSS

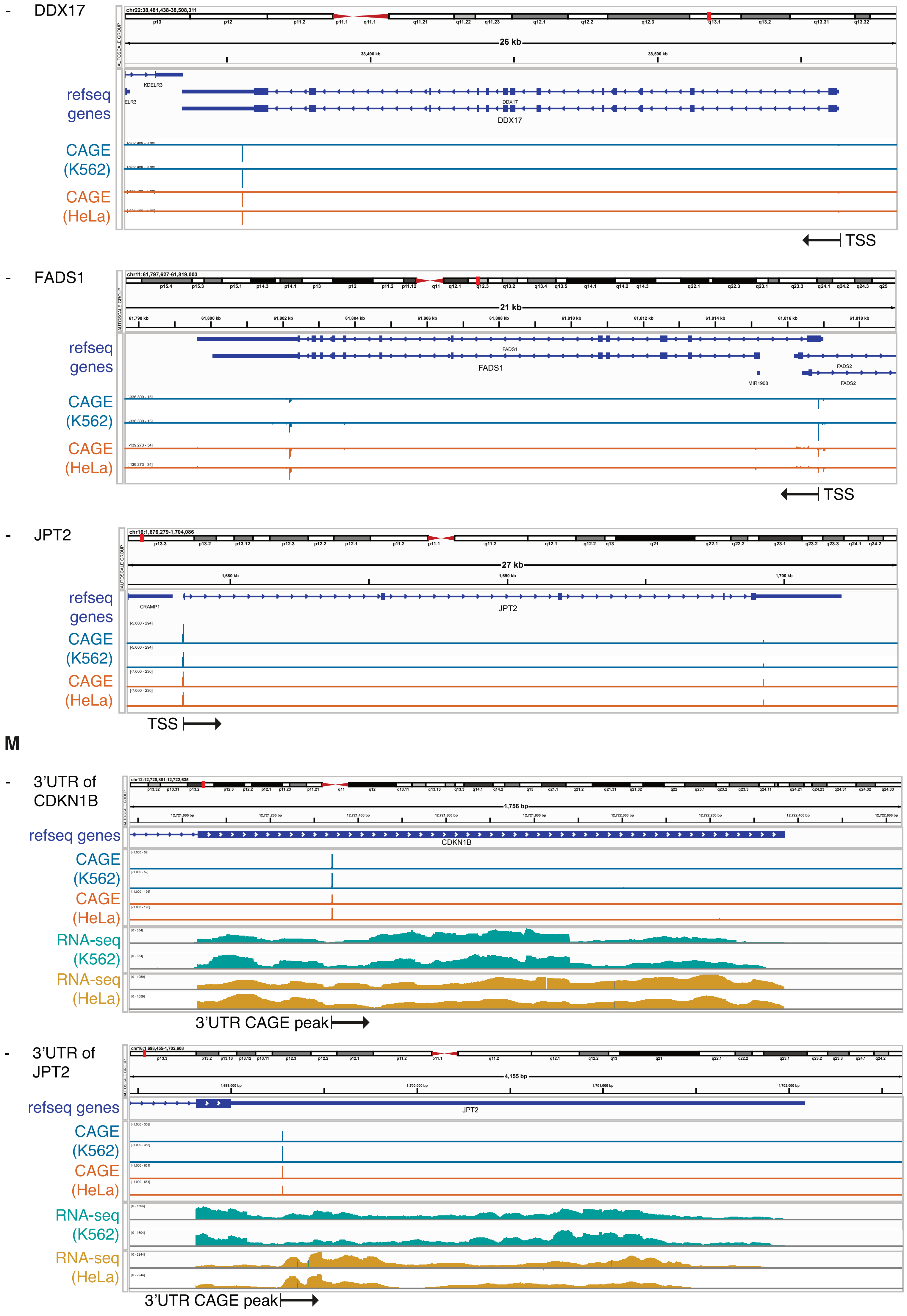

DDX17
chr22:38,481,438-38,508,311
26 kb
38,490 kb
38,500 kb
refseq genes
KDELR3
DDX17
CAGE (K562)
CAGE (HeLa)
TSS
FADS1
chr11:61,797,627-61,819,003
21 kb
refseq genes
FADS1
FADS2
MIR1908
CAGE (K562)
CAGE (HeLa)
TSS
JPT2
chr16:1,676,279-1,704,086
27 kb
1,680 kb
1,690 kb
1,700 kb
refseq genes
CRAMP1
JPT2
CAGE (K562)
CAGE (HeLa)
TSS
M
3'UTR of CDKN1B
chr12:12,720,881-12,722,635
1,756 bp
refseq genes
CDKN1B
CAGE (K562)
CAGE (HeLa)
RNA-seq (K562)
RNA-seq (HeLa)
3'UTR CAGE peak
3'UTR of JPT2
chr16:1,698,455-1,702,608
4,155 bp
1,699,000 bp
1,700,000 bp
1,701,000 bp
1,702,000 bp
refseq genes
JPT2
CAGE (K562)
CAGE (HeLa)
RNA-seq (K562)
RNA-seq (HeLa)
3'UTR CAGE peak

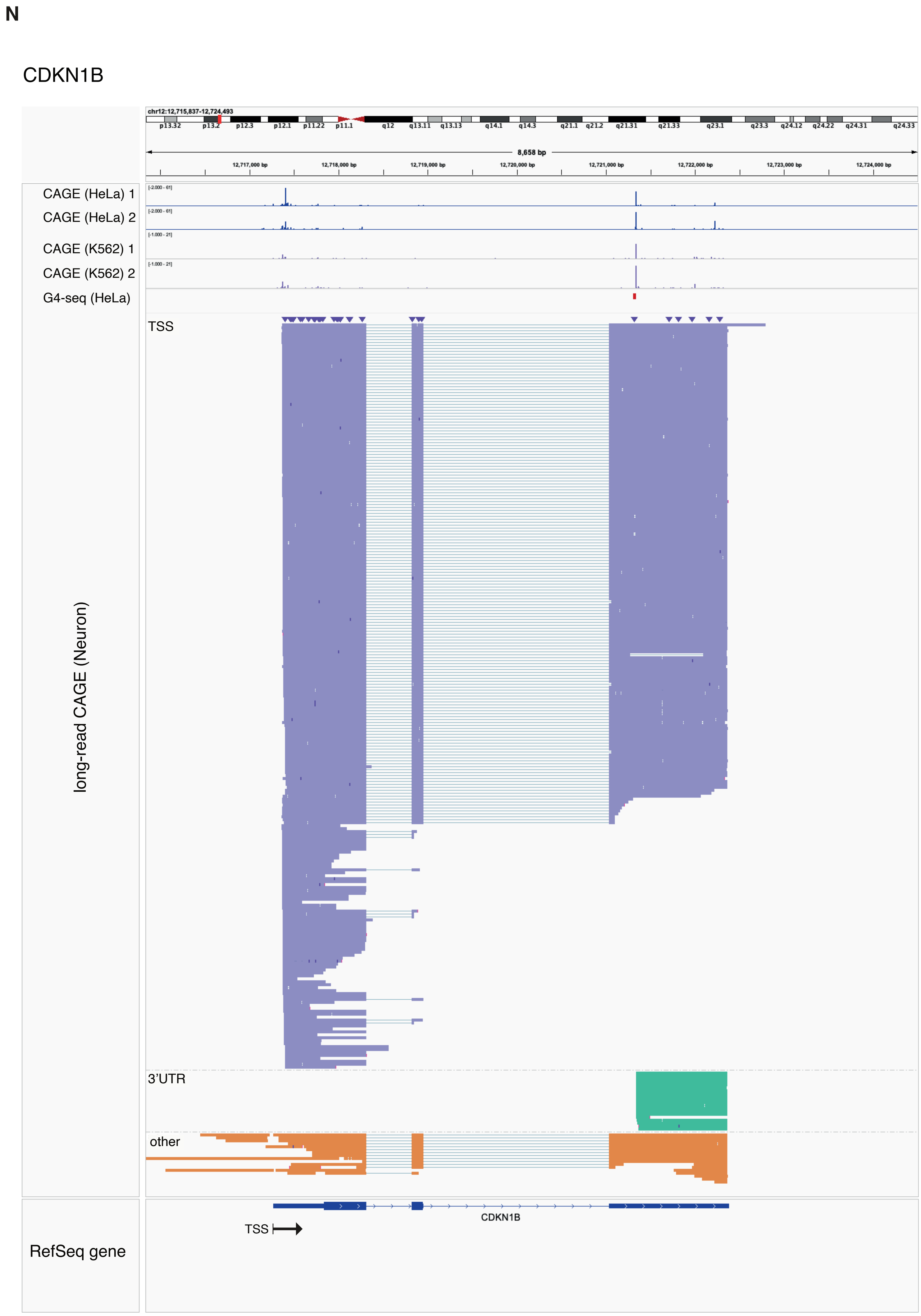

N
CDKN1B
chr12:12,715,837-12,724,493
8,658 bp
12,717,000 bp
12,718,000 bp
12,719,000 bp
12,720,000 bp
12,721,000 bp
12,722,000 bp
12,723,000 bp
12,724,000 bp
CAGE (HeLa) 1
CAGE (HeLa) 2
CAGE (K562) 1
CAGE (K562) 2
G4-seq (HeLa)
long-read CAGE (Neuron)
TSS
3'UTR
other
RefSeq gene
CDKN1B
TSS

JPT2

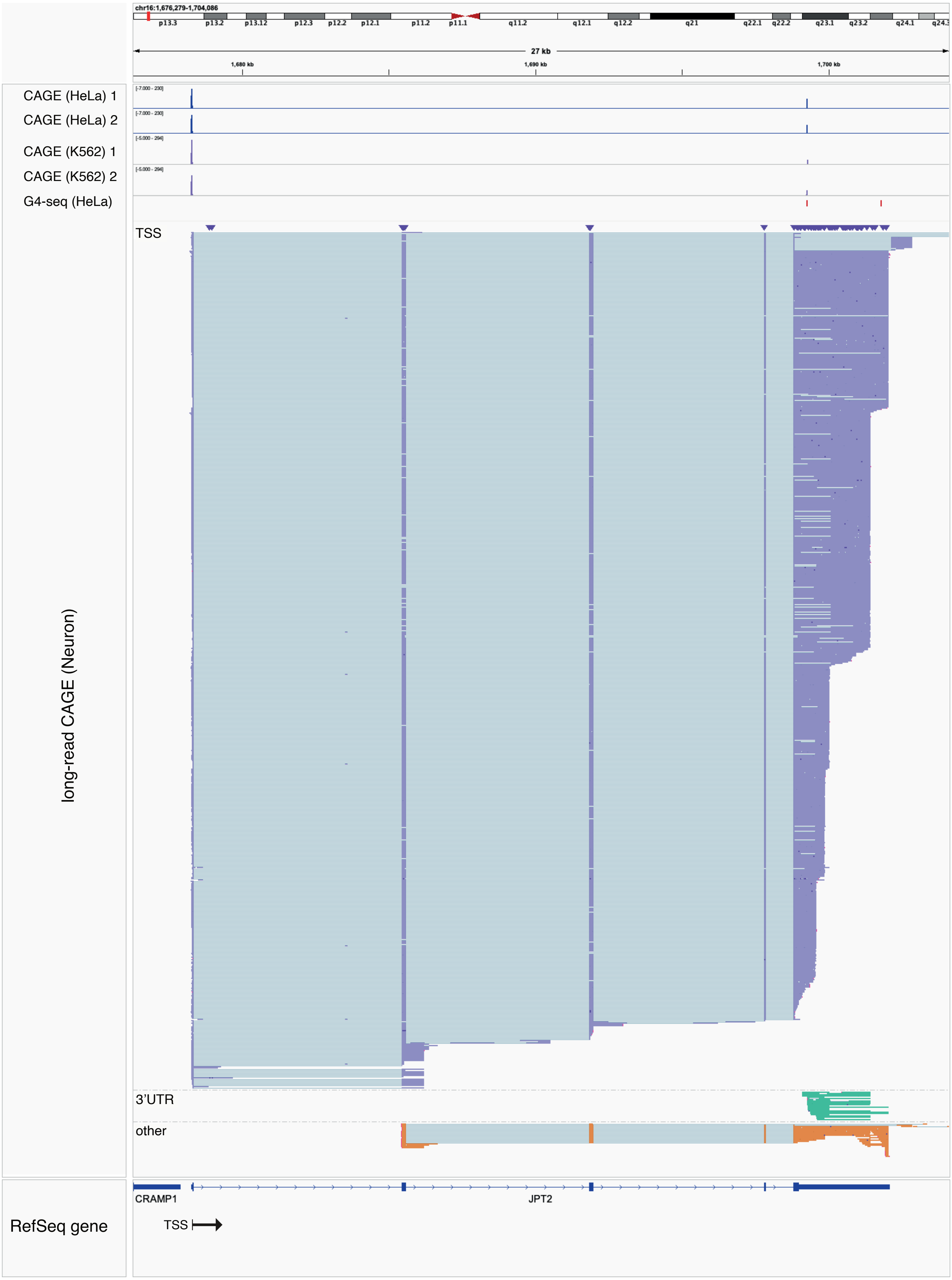

chr16:1,676,279-1,704,086
p13.3
p13.2
p13.12
p12.3
p12.2
p12.1
p11.2
p11.1
q11.2
q12.1
q12.2
q21
q22.1
q22.2
q23.1
q23.2
q24.1
27 kb
1,680 kb
1,690 kb
1,700 kb
CAGE (HeLa) 1
CAGE (HeLa) 2
CAGE (K562) 1
CAGE (K562) 2
G4-seq (HeLa)
[-7.000 - 230]
[-5.000 - 294]
TSS
long-read CAGE (Neuron)
3'UTR
other
RefSeq gene
CRAMP1
JPT2
TSS

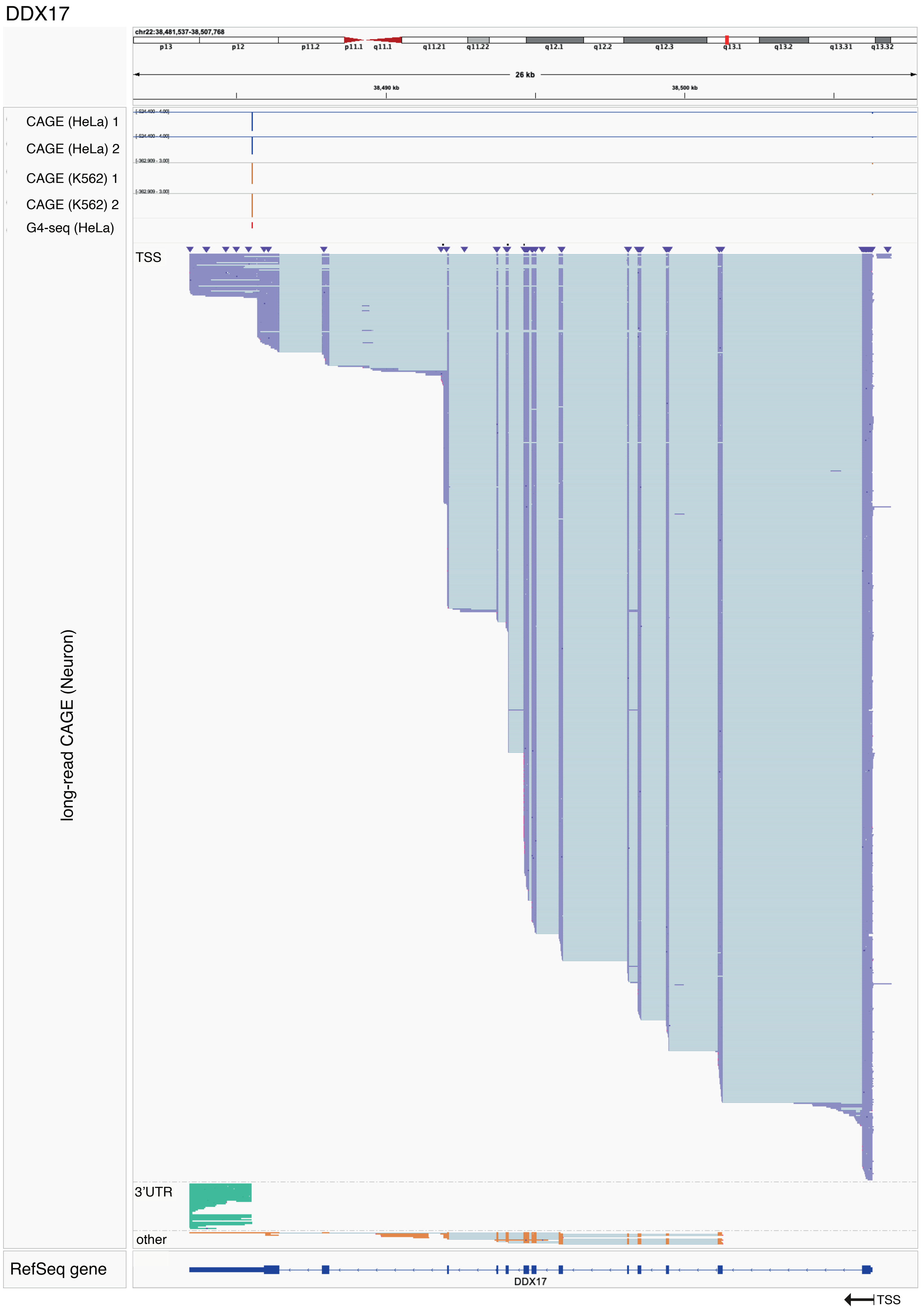

DDX17
chr22:38,481,537-38,507,768
p13
p12
p11.2
p11.1
q11.1
q11.21
q11.22
q12.1
q12.2
q12.3
q13.1
q13.2
q13.31
q13.32
26 kb
38,490 kb
38,500 kb
CAGE (HeLa) 1
CAGE (HeLa) 2
CAGE (K562) 1
CAGE (K562) 2
G4-seq (HeLa)
TSS
long-read CAGE (Neuron)
3'UTR
other
RefSeq gene
DDX17
TSS

ARPC5L

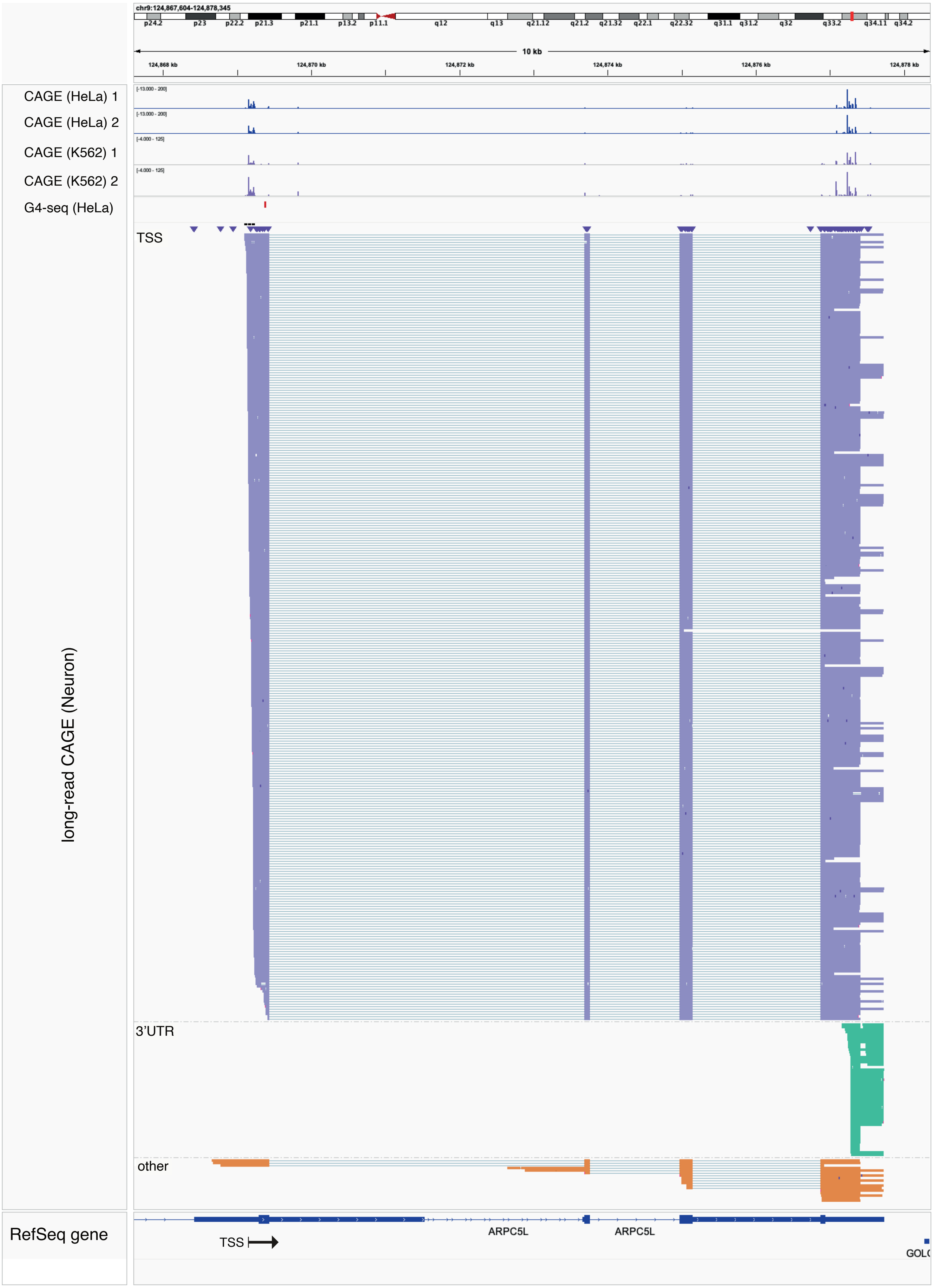

chr9:124,867,604-124,878,345
p24.2
p23
p22.2
p21.3
p21.1
p13.2
p11.1
q12
q13
q21.12
q21.2
q21.32
q22.1
q22.32
q31.1
q31.2
q32
q33.2
q34.11
q34.2
10 kb
124,868 kb
124,870 kb
124,872 kb
124,874 kb
124,876 kb
124,878 kb
CAGE (HeLa) 1
[-13.000 - 200]
CAGE (HeLa) 2
[-13.000 - 200]
CAGE (K562) 1
[-4.000 - 125]
CAGE (K562) 2
[-4.000 - 125]
G4-seq (HeLa)
long-read CAGE (Neuron)
TSS
3'UTR
other
RefSeq gene
ARPC5L
ARPC5L
TSS
GOL(

CSDE1

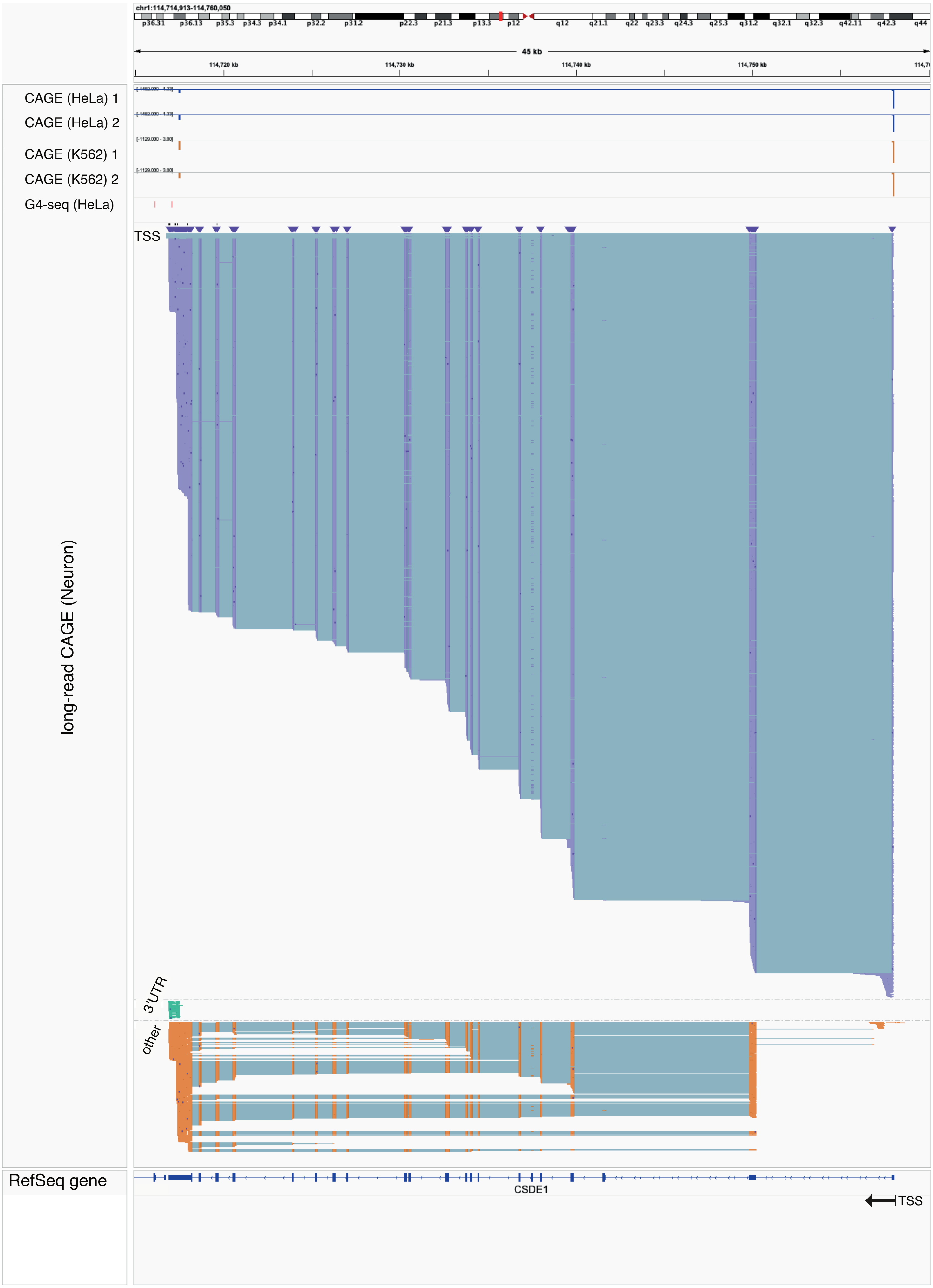

chr1:114,714,913-114,760,050
45 kb
114,720 kb
114,730 kb
114,740 kb
114,750 kb
CAGE (HeLa) 1
CAGE (HeLa) 2
CAGE (K562) 1
CAGE (K562) 2
G4-seq (HeLa)
TSS
long-read CAGE (Neuron)
3'UTR
other
RefSeq gene
CSDE1
TSS

GHITM

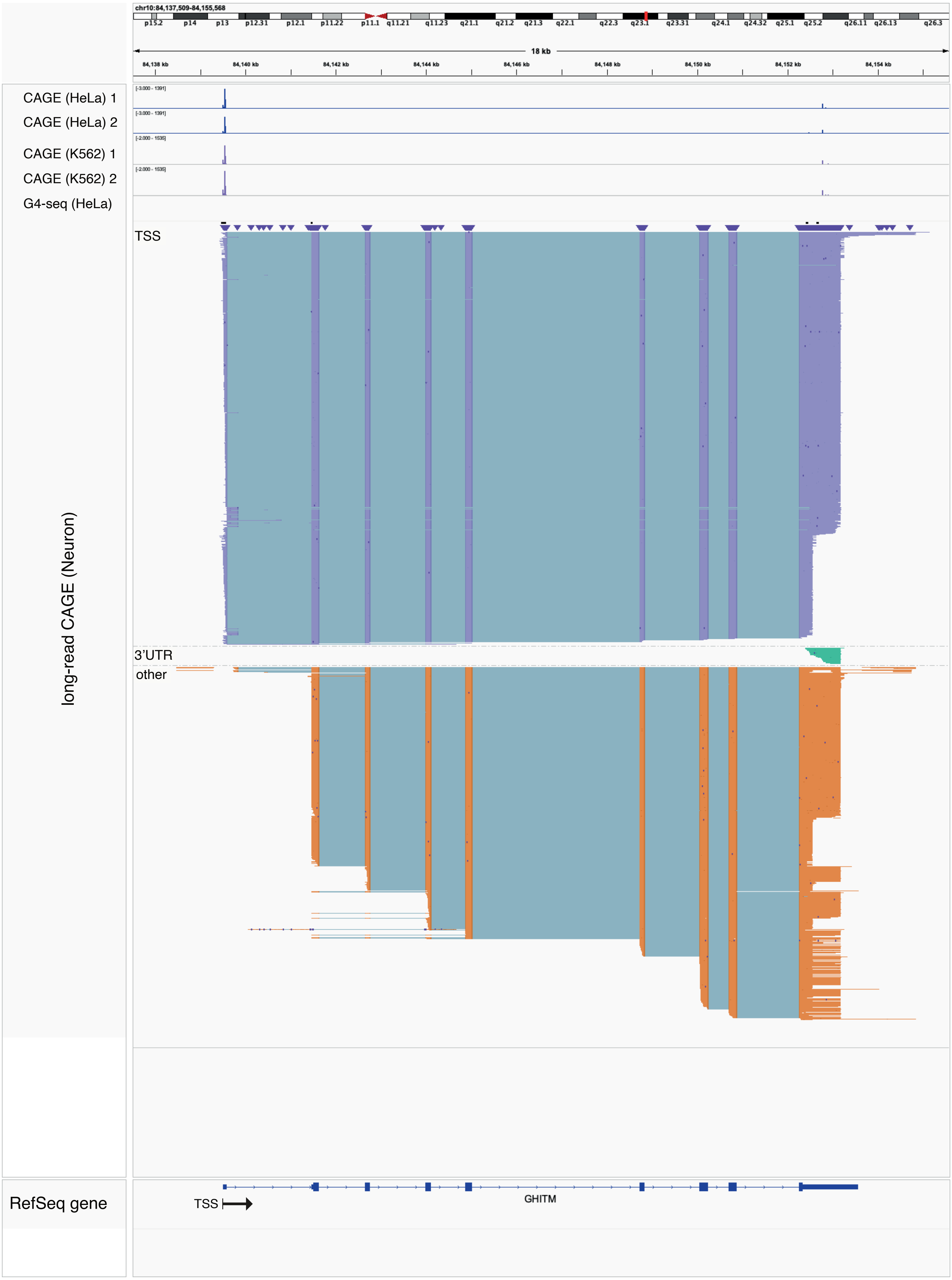

chr10:84,137,509-84,155,568
p15.2
p14
p13
p12.31
p12.1
p11.22
p11.1
q11.21
q11.23
q21.1
q21.2
q21.3
q22.1
q22.3
q23.1
q23.31
q24.1
q24.32
q25.1
q25.2
q26.11
q26.13
q26.3
18 kb
84,138 kb
84,140 kb
84,142 kb
84,144 kb
84,146 kb
84,148 kb
84,150 kb
84,152 kb
84,154 kb
CAGE (HeLa) 1
CAGE (HeLa) 2
CAGE (K562) 1
CAGE (K562) 2
G4-seq (HeLa)
[-3.000 - 1391]
[-3.000 - 1391]
[-2.000 - 1535]
[-2.000 - 1535]
TSS
3'UTR
other
long-read CAGE (Neuron)
RefSeq gene
TSS
GHITM

TNPO3

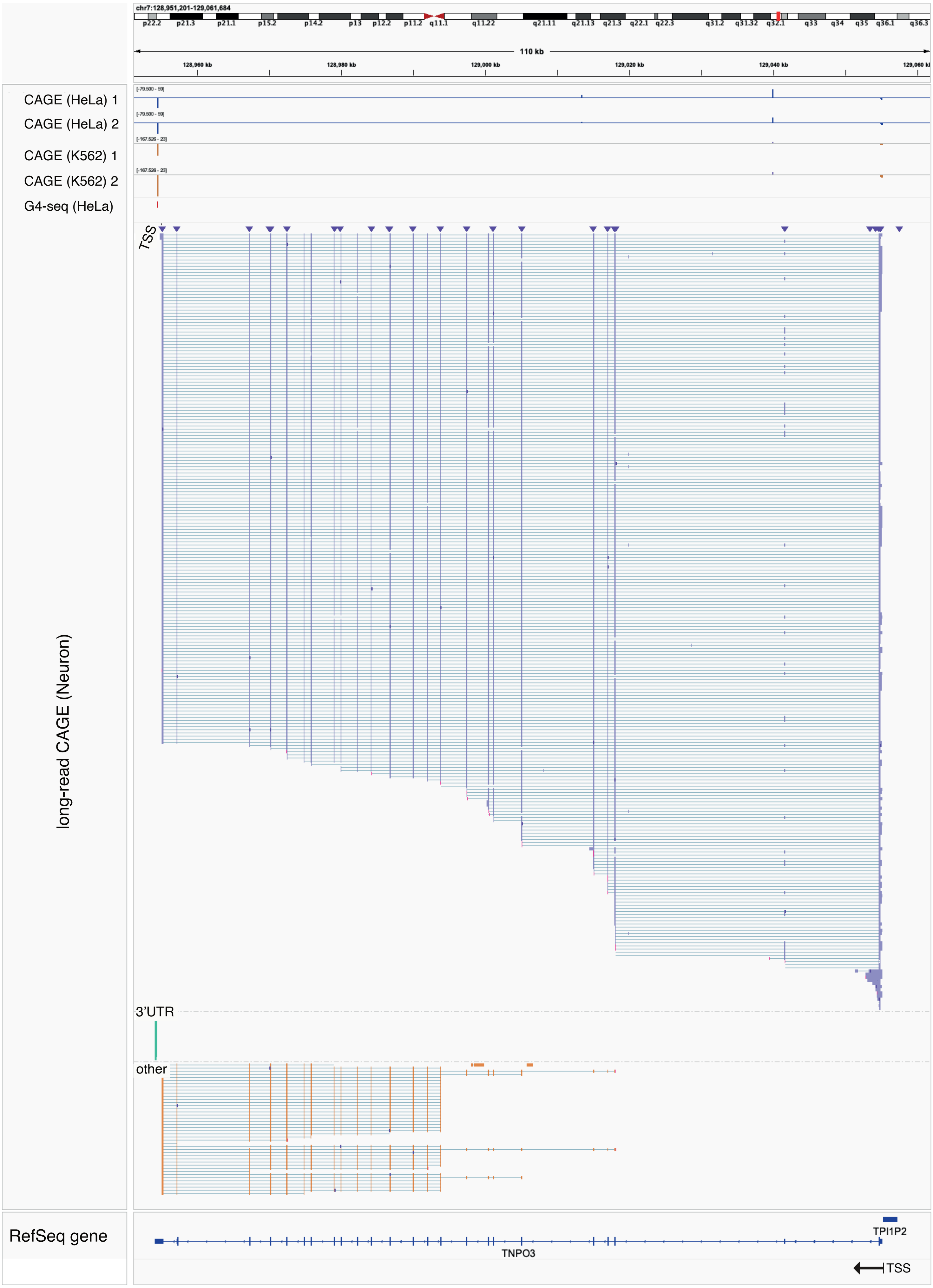
chr7:128,951,201-129,061,684
p22.2
p21.3
p21.1
p15.2
p14.2
p13
p12.2
p11.2
q11.1
q11.22
q21.11
q21.13
q21.3
q22.1
q22.3
q31.2
q31.32
q32.1
q33
q34
q35
q36.1
q36.3
110 kb
128,960 kb
128,980 kb
129,000 kb
129,020 kb
129,040 kb
CAGE (HeLa) 1
CAGE (HeLa) 2
CAGE (K562) 1
CAGE (K562) 2
G4-seq (HeLa)
TSS
long-read CAGE (Neuron)
3'UTR
other
RefSeq gene
TPI1P2
TNPO3
TSS

ZFAND3

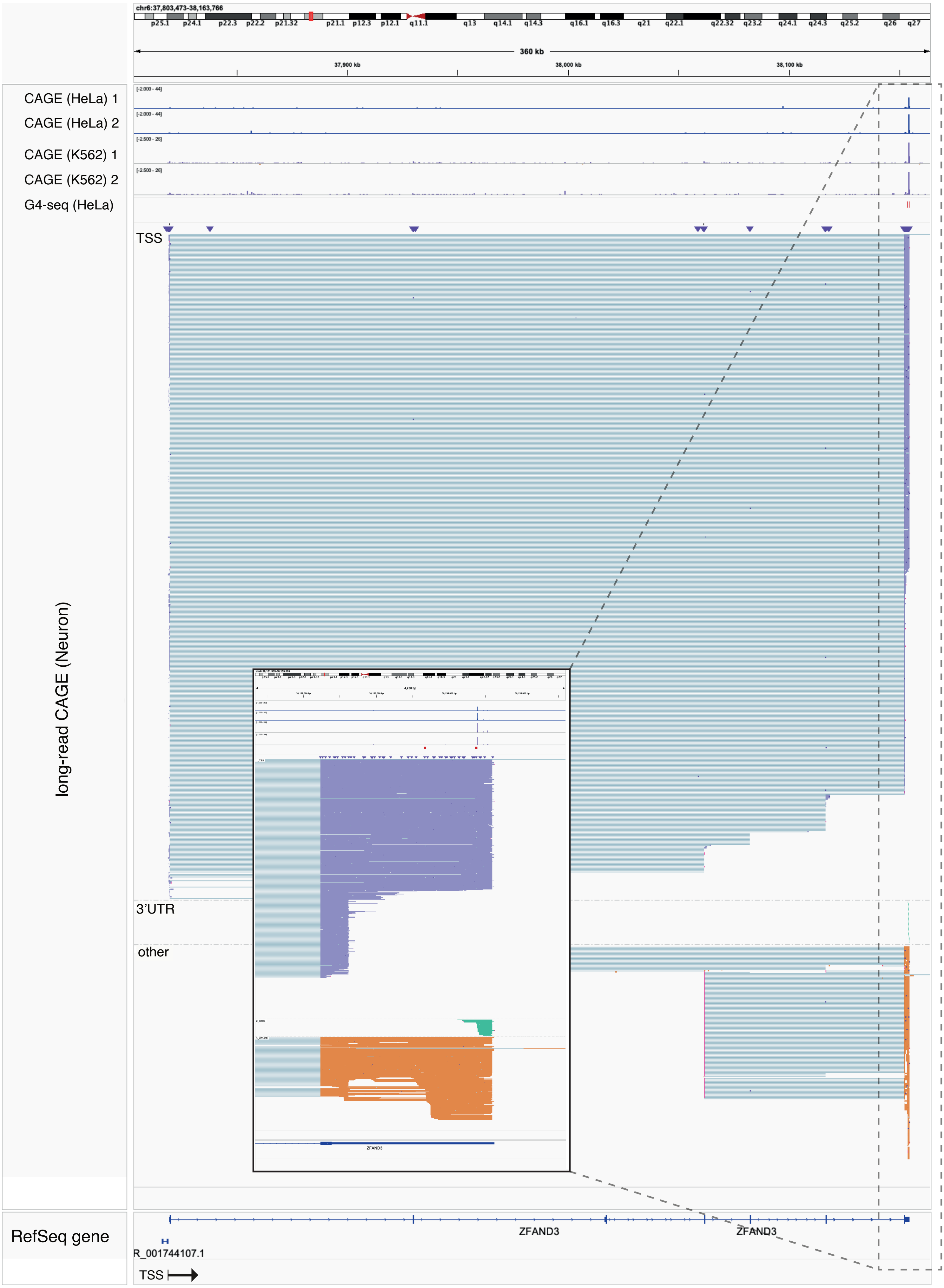

chr6:37,803,473-38,163,766
360 kb
37,900 kb
38,000 kb
38,100 kb
CAGE (HeLa) 1
CAGE (HeLa) 2
CAGE (K562) 1
CAGE (K562) 2
G4-seq (HeLa)
TSS
long-read CAGE (Neuron)
3'UTR
other
RefSeq gene
ZFAND3
ZFAND3
R_001744107.1
TSS

APP

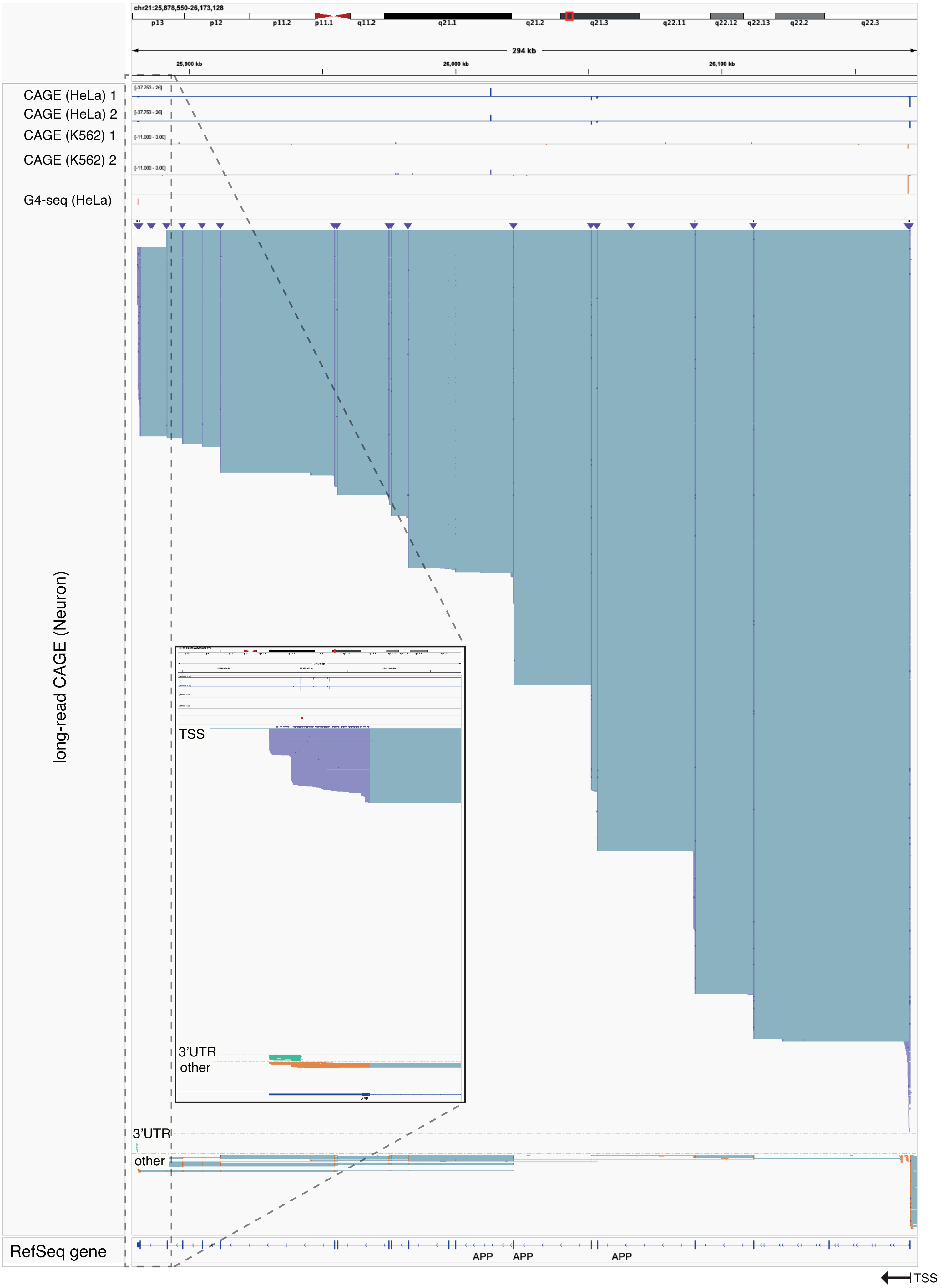

chr21:25,878,550-26,173,128
p13
p12
p11.2
p11.1
q11.2
q21.1
q21.2
q21.3
q22.11
q22.12
q22.13
q22.2
q22.3
294 kb
25,900 kb
26,000 kb
26,100 kb
CAGE (HeLa) 1
CAGE (HeLa) 2
CAGE (K562) 1
CAGE (K562) 2
G4-seq (HeLa)
long-read CAGE (Neuron)
TSS
3'UTR
other
3'UTR
other
RefSeq gene
APP
APP
APP
TSS

CCN1

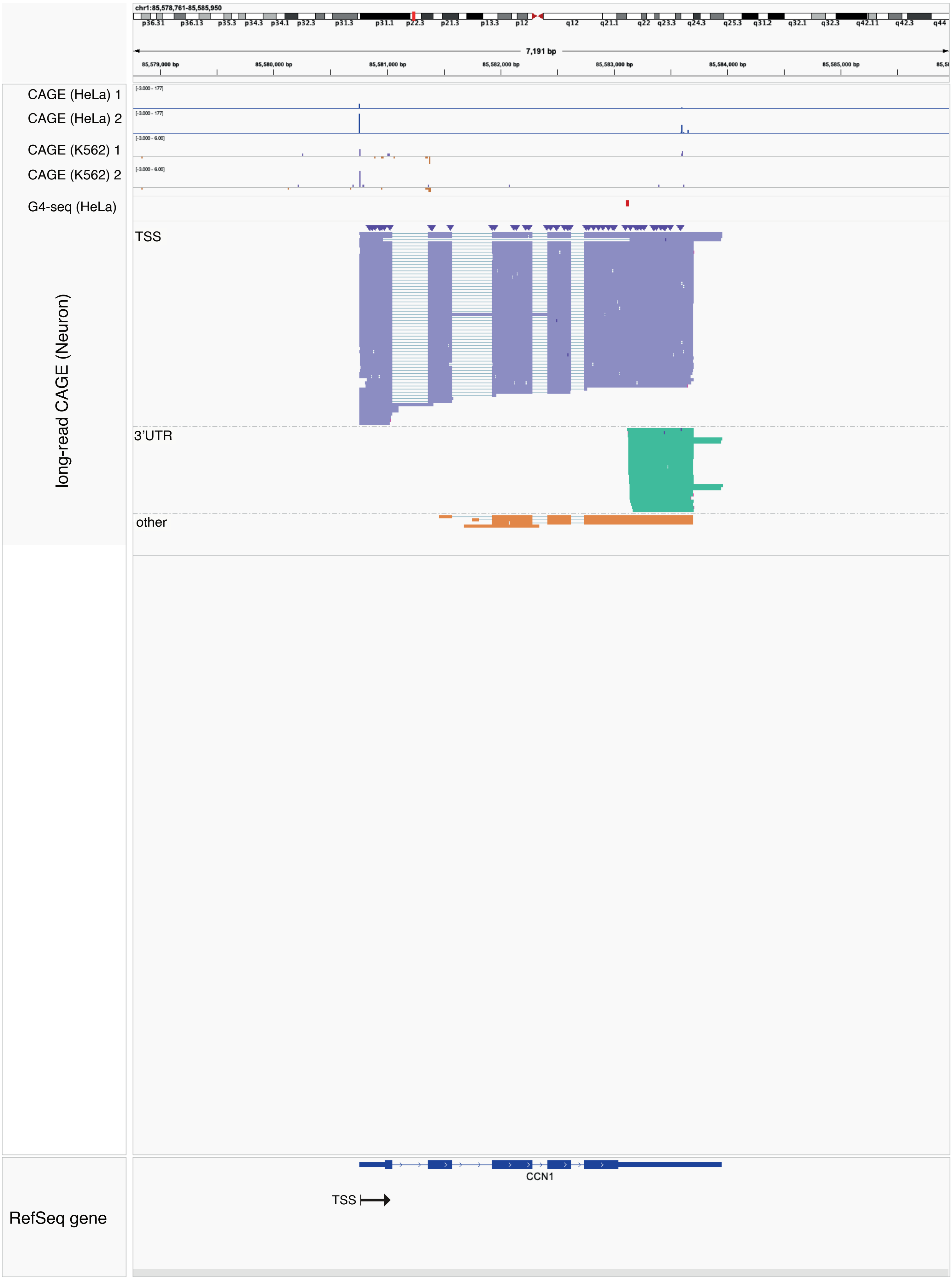

chr1:85,578,761-85,585,950
7,191 bp
85,579,000 bp
85,580,000 bp
85,581,000 bp
85,582,000 bp
85,583,000 bp
85,584,000 bp
85,585,000 bp
CAGE (HeLa) 1
CAGE (HeLa) 2
CAGE (K562) 1
CAGE (K562) 2
G4-seq (HeLa)
long-read CAGE (Neuron)
TSS
3’UTR
other
RefSeq gene
CCN1
TSS
