# Supplementary material for: Widespread 3′UTR capped RNAs derive from G-rich regions in proximity to AGO2 binding sites
Source: BMC Biol. 2024 Nov 7;22:254. doi: 10.1186/s12915-024-02032-7 (PMC11546257; doi:10.1186/s12915-024-02032-7)
Supplement: Supplementary file 1 — Additional file 1: Figures S1, S2, S3, S4. Each supplementary figure corresponds to the main Figs. in the manuscript, in the same order. Fig. S1 Related to Fig. 1. A Pearson’s correlation of raw CAGE read counts per TSS or consensus cluster across biological replicates and cell types. B Reverse cumulative distribution of CAGE reads after normalisation using CAGEr package [82]. C Total number of CAGE reads in each sample. D Density of total 5′ CAGE read positions normalised by the length of the correspondent transcript region identified in CAGE-seq libraries of K562 and HeLa samples with two biological replicates each, provided by ENCODE. E Percentage of CAGE tags per transcript region using random primers, Oligo(dT) primers, and combination of both primers (1:4 Oligo(dT):Random primers) in CAGE-seq libraries of THP-1 cells generated by RIKEN. F-I Pearson’s correlation between CAGE-seq replicates and different cell lines samples in 3′UTRs, 5′UTRs, CDS and introns. J Top: Plot of the normalised coverage of the 5′ ends of forward paired-end reads (yellow line) and 3′ ends of reverse paired-end reads (blue line) of RNA-seq relative to 3′UTR CAGE peaks in HeLa cells. Bottom: Schematic representation of paired-end read positioning. Forward and reversed paired-end reads are presented in yellow and blue, respectively. The black box represents the ends of reads that are plotted in the top graph. K RT-qPCR data of gene expression using primers designed to amplify sequences located downstream (3'C), upstream (5'C) and overlapping (AC) the 3′UTR CAGE sites of CDKN1B and JPT2. Data represents fold detection (six biological replicates) using downstream versus upstream/overlapping primers relative to the 3′UTR CAGE peaks. Primer target sequences relative to the 3′UTR CAGE peak are schematically represented on the top right-hand side and visualised for each gene using IGV genome browser on the bottom. Each dot represents the value of an independent biological replicate. L Top gene [file 12915_2024_2032_MOESM1_ESM.zip › Additional_File_1_Figure_S3_ESM.pdf]

A

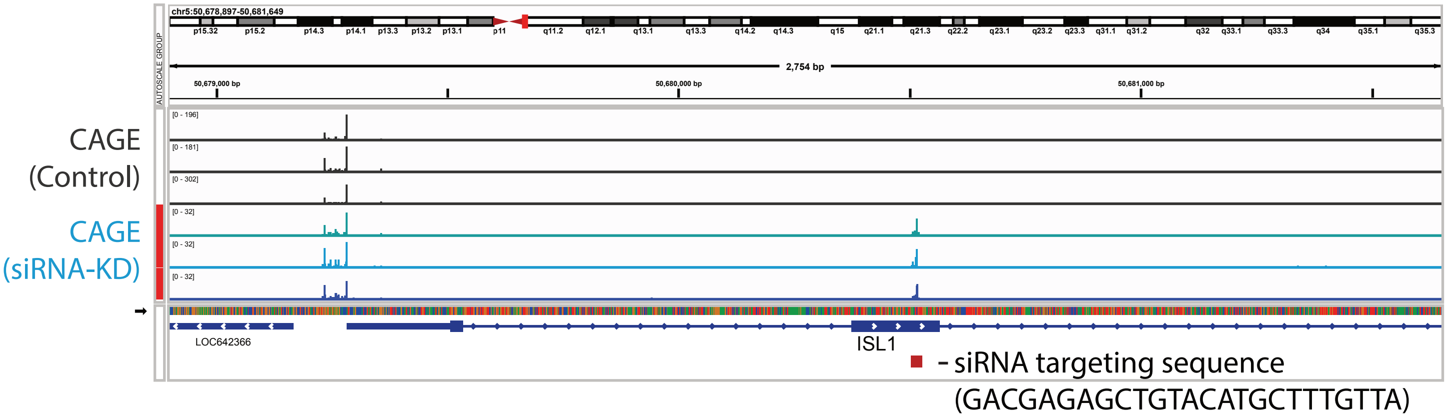

chr5:50,678,897-50,681,649
p15.32
p15.2
p14.3
p14.1
p13.3
p13.2
p13.1
p11
q11.2
q12.1
q13.1
q13.3
q14.2
q14.3
q15
q21.1
q21.3
q22.2
q23.1
q23.2
q23.3
q31.1
q31.2
q32
q33.1
q33.3
q34
q35.1
q35.3
AUTOSCALE GROUP
2,754 bp
50,679,000 bp
50,680,000 bp
50,681,000 bp
CAGE
(Control)
CAGE
(siRNA-KD)
[0 - 196]
[0 - 181]
[0 - 302]
[0 - 32]
[0 - 32]
[0 - 32]
LOC642366
ISL1
- siRNA targeting sequence
(GACGAGAGCTGTACATGCTTTGTTA)

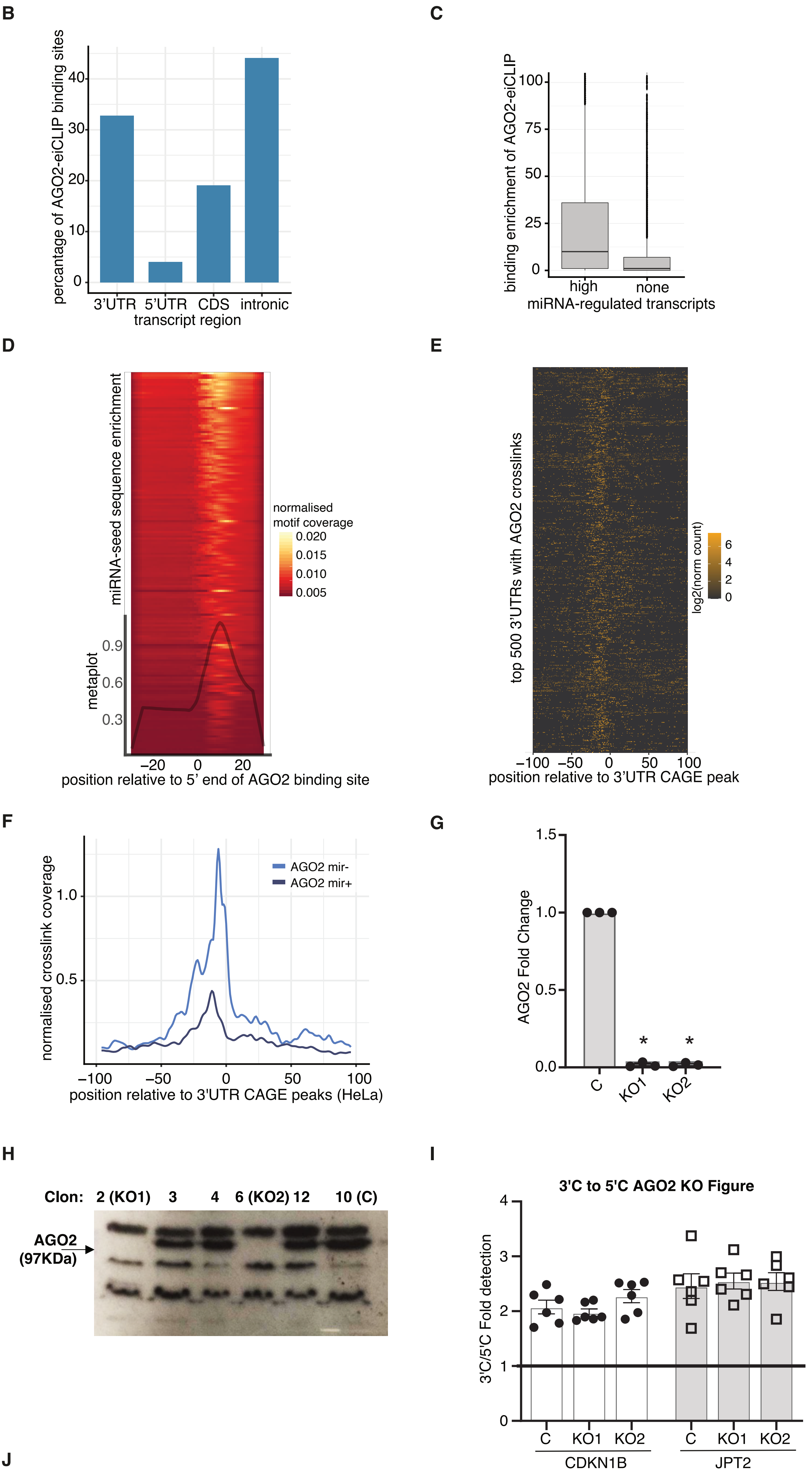

J

Homer de novo Motif Results
Total target sequences = 172773
Total background sequences = 177500
* - possible false positive

Rank	Motif	P-value	log P-pvalue	% of Targets	% of Background	STD(Bg STD)	Best Match/Details
1	GUCGAAAU	1e-5254	-1.210e+04	1.06%	0.00%	1.0bp (0.0bp)	hsa-miR-1305 MIMAT0005893 Homo sapiens miR-1305 Targets (miRBase)(0.637) More Information | Similar Motifs Found
2	GGUGAGGU	1e-2229	-5.133e+03	1.19%	0.04%	1.7bp (2.9bp)	hsa-miR-4777-3p MIMAT0019935 Homo sapiens miR-4777-3p Targets (miRBase)(0.743) More Information | Similar Motifs Found
3	CGUGGGGG	1e-817	-1.882e+03	18.44%	13.19%	2.5bp (3.1bp)	hsa-miR-361-3p MIMAT0004682 Homo sapiens miR-361-3p Targets (miRBase)(0.701) More Information | Similar Motifs Found
4	UUAAAGCA	1e-759	-1.749e+03	0.45%	0.02%	0.8bp (2.1bp)	hsa-miR-1273e MIMAT0018079 Homo sapiens miR-1273e Targets (miRBase)(0.699) More Information | Similar Motifs Found
5	CCAGAAGA	1e-689	-1.589e+03	12.21%	8.24%	2.4bp (2.8bp)	hsa-miR-4659b-3p MIMAT0019734 Homo sapiens miR-4659b-3p Targets (miRBase)(0.684) More Information | Similar Motifs Found
6	UAAUAUUG	1e-331	-7.632e+02	5.13%	3.32%	2.7bp (2.5bp)	hsa-miR-16-2* MIMAT0004518 Homo sapiens miR-16-2* Targets (miRBase)(0.724) More Information | Similar Motifs Found
7	CCACUAAU	1e-244	-5.623e+02	17.25%	14.35%	2.4bp (2.2bp)	hsa-miR-4727-3p MIMAT0019848 Homo sapiens miR-4727-3p Targets (miRBase)(0.676) More Information | Similar Motifs Found
8	UUGUUUGU	1e-135	-3.119e+02	0.97%	0.50%	2.7bp (2.7bp)	hsa-miR-495 MIMAT0002817 Homo sapiens miR-495 Targets (miRBase)(0.665) More Information | Similar Motifs Found
9	CCCCCCCC	1e-107	-2.479e+02	4.08%	3.12%	2.4bp (2.0bp)	hsa-miR-4488 MIMAT0019022 Homo sapiens miR-4488 Targets (miRBase)(0.743) More Information | Similar Motifs Found
10	AAACCCGU	1e-79	-1.824e+02	0.04%	0.00%	1.0bp (1.6bp)	hsa-miR-125b-1* MIMAT0004592 Homo sapiens miR-125b-1* Targets (miRBase)(0.699) More Information | Similar Motifs Found
11	CUAAACGC	1e-75	-1.743e+02	0.03%	0.00%	1.3bp (1.5bp)	hsa-miR-629 MIMAT0004810 Homo sapiens miR-629 Targets (miRBase)(0.642) More Information | Similar Motifs Found
12	GCAGGGAG	1e-70	-1.618e+02	0.25%	0.09%	2.3bp (2.9bp)	hsa-miR-3620 MIMAT0018001 Homo sapiens miR-3620 Targets (miRBase)(0.716) More Information | Similar Motifs Found

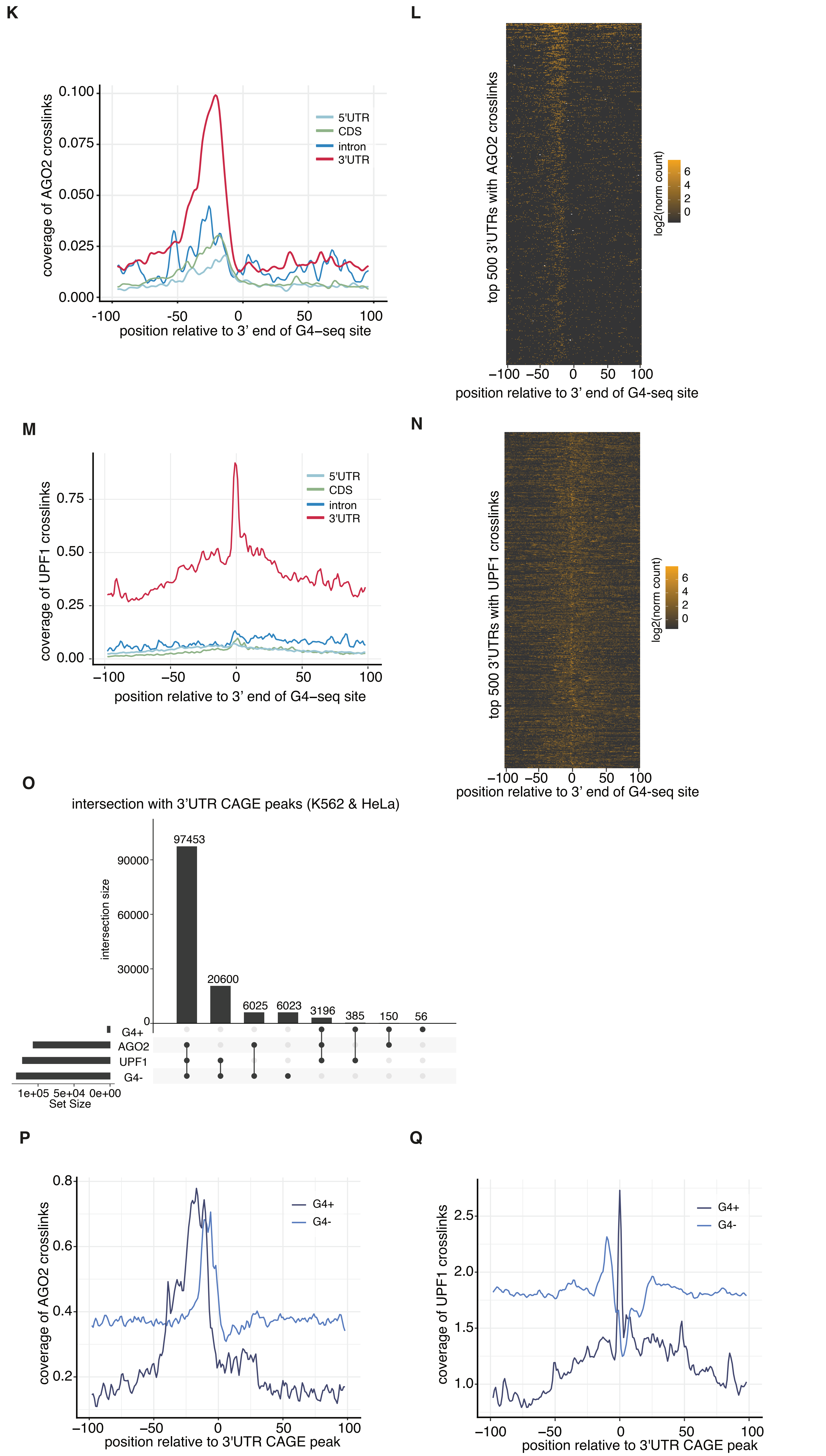

K
L
M
N
O
P
Q
coverage of AGO2 crosslinks
position relative to 3' end of G4–seq site
5'UTR
CDS
intron
3'UTR
top 500 3'UTRs with AGO2 crosslinks
log2(norm count)
coverage of UPF1 crosslinks
top 500 3'UTRs with UPF1 crosslinks
intersection with 3'UTR CAGE peaks (K562 & HeLa)
intersection size
97453
20600
6025
6023
3196
385
150
56
G4+
AGO2
UPF1
G4-
Set Size
position relative to 3'UTR CAGE peak
