# Supplementary figures and images for: Widespread 3′UTR capped RNAs derive from G-rich regions in proximity to AGO2 binding sites
Source: BMC Biol. 2024 Nov 7;22:254. doi: 10.1186/s12915-024-02032-7 (PMC11546257; doi:10.1186/s12915-024-02032-7)

**A**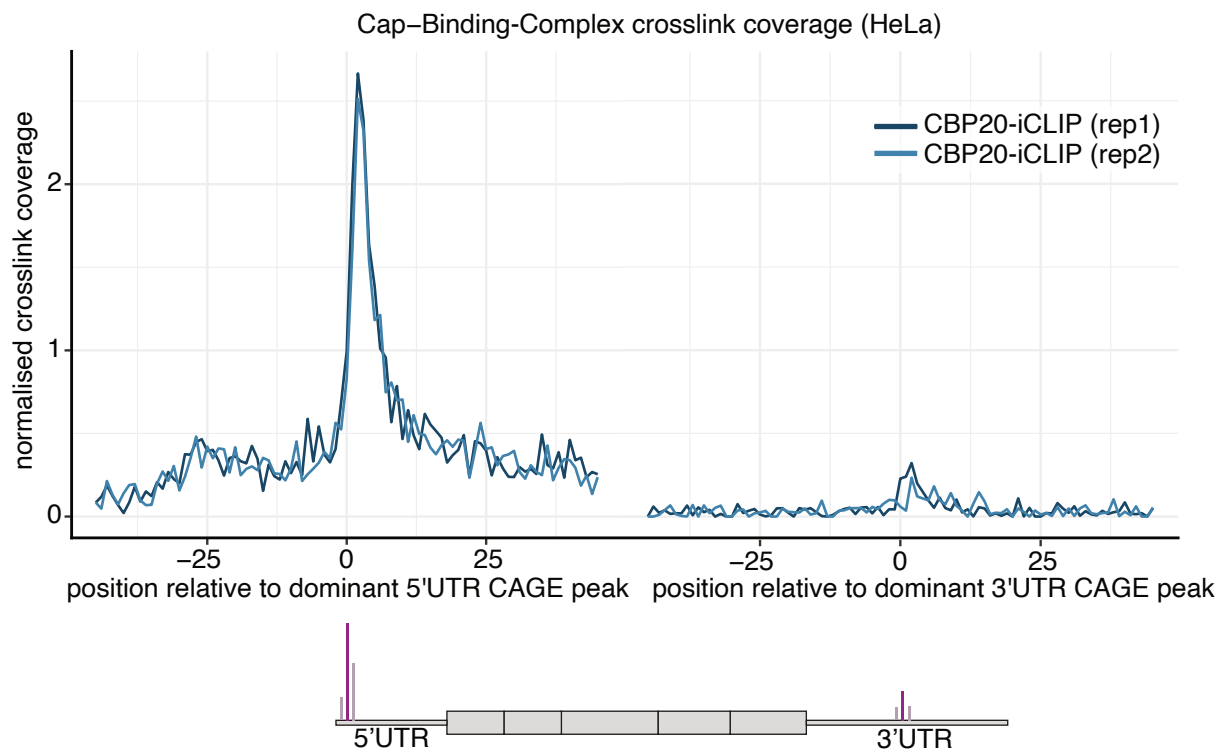**B**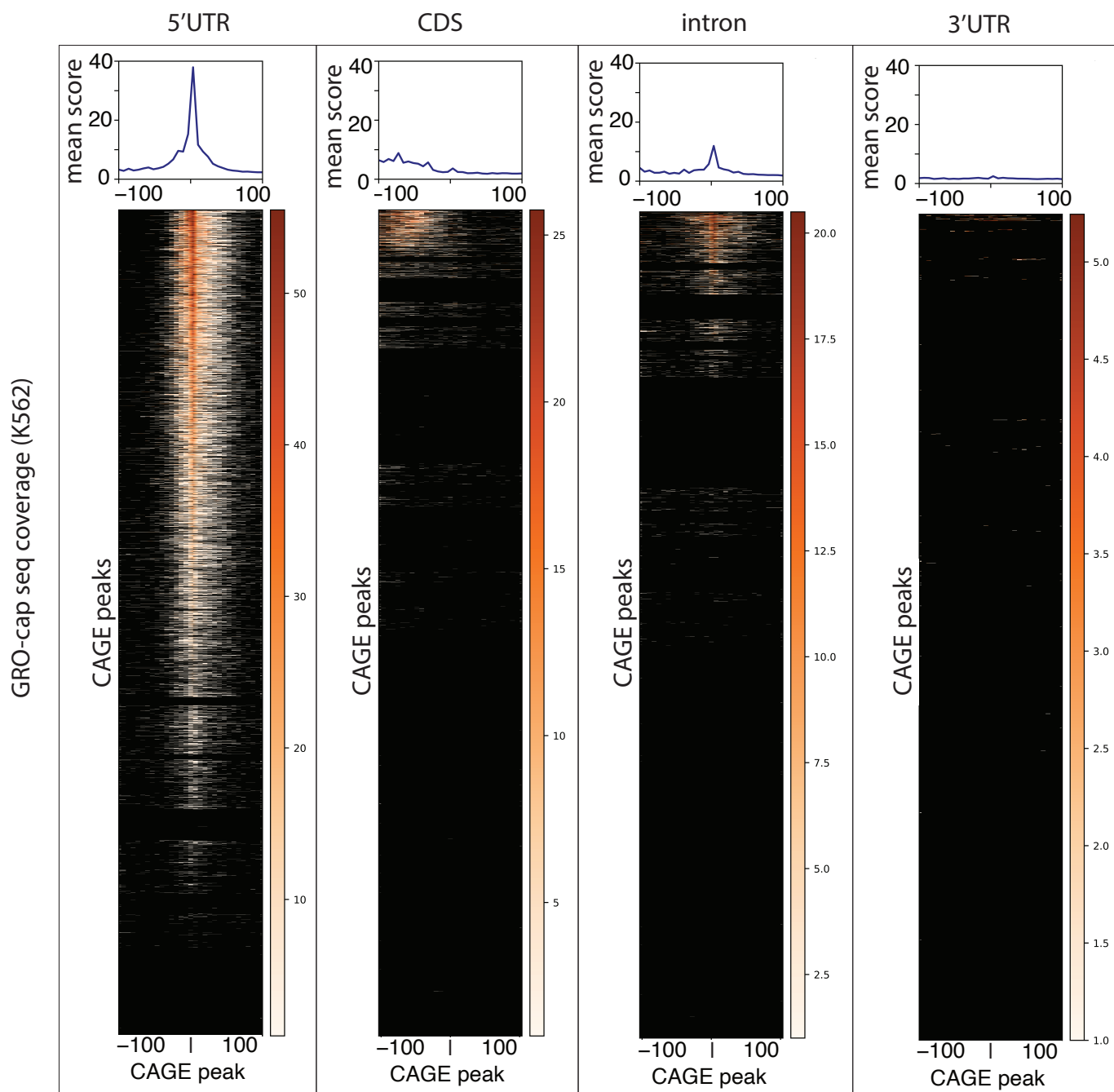

**C**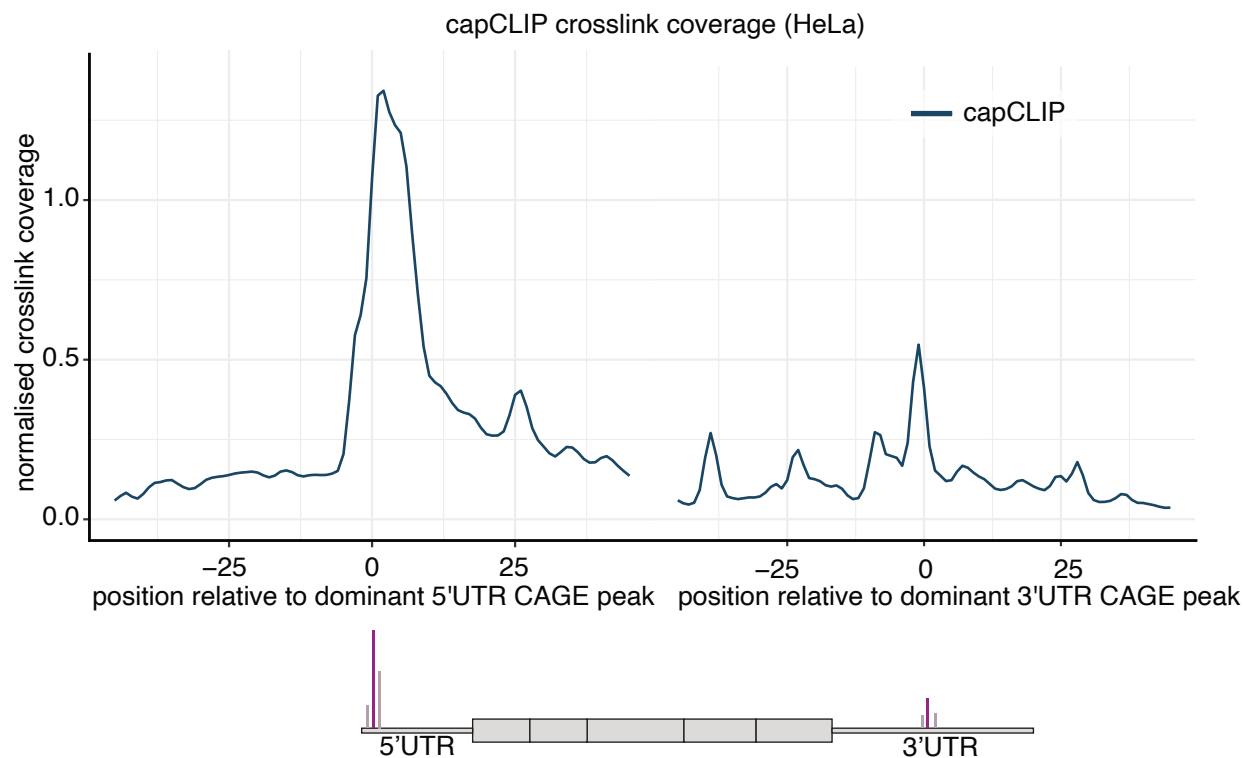**D**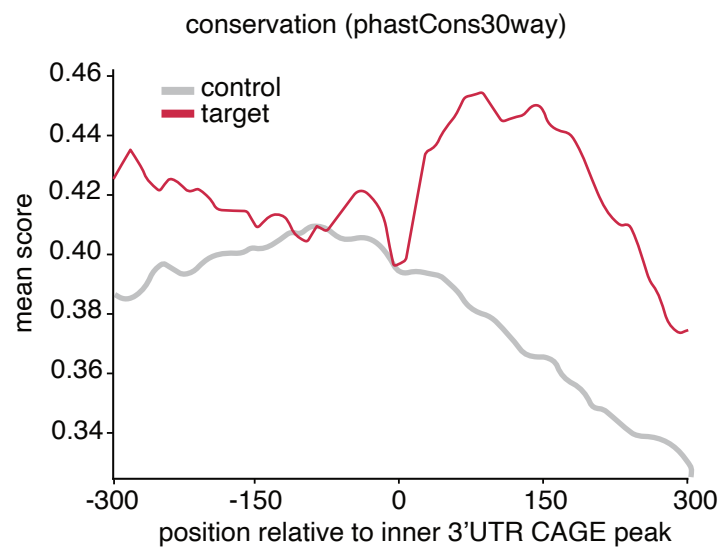

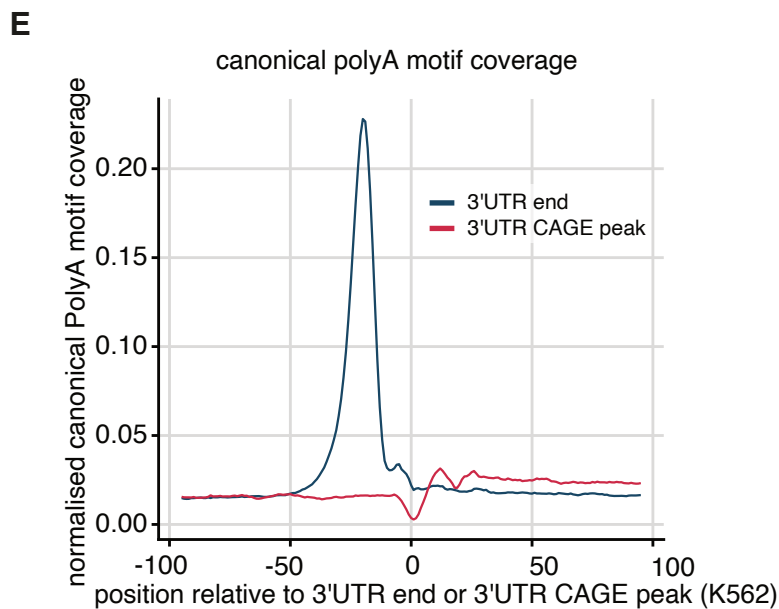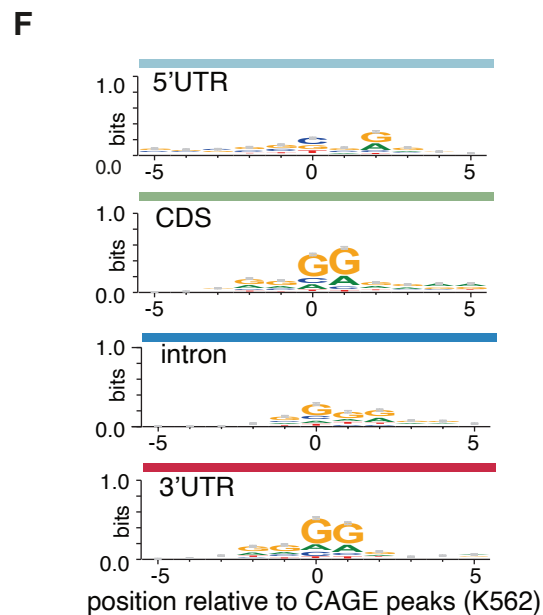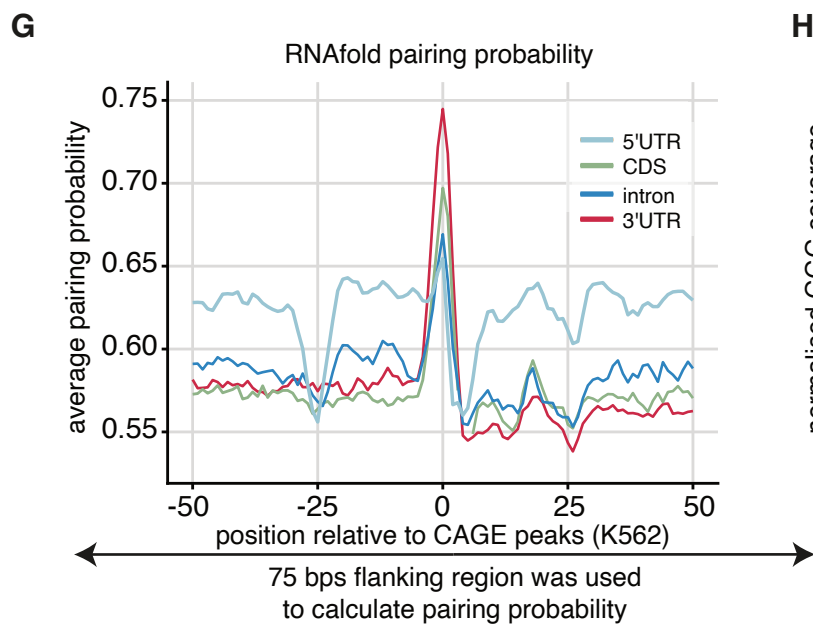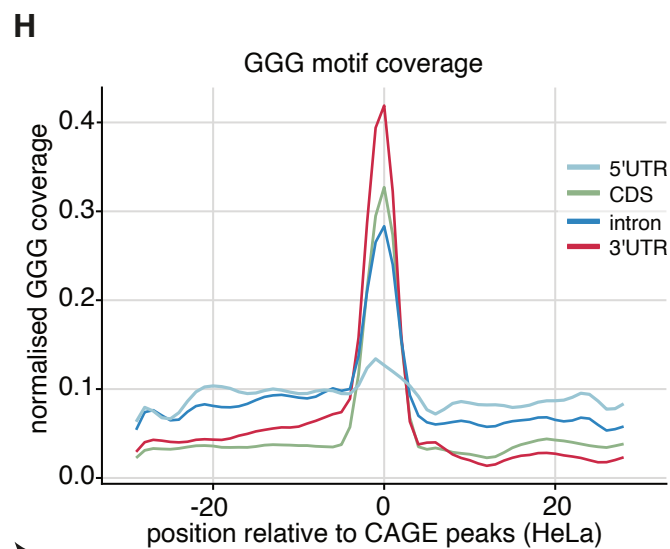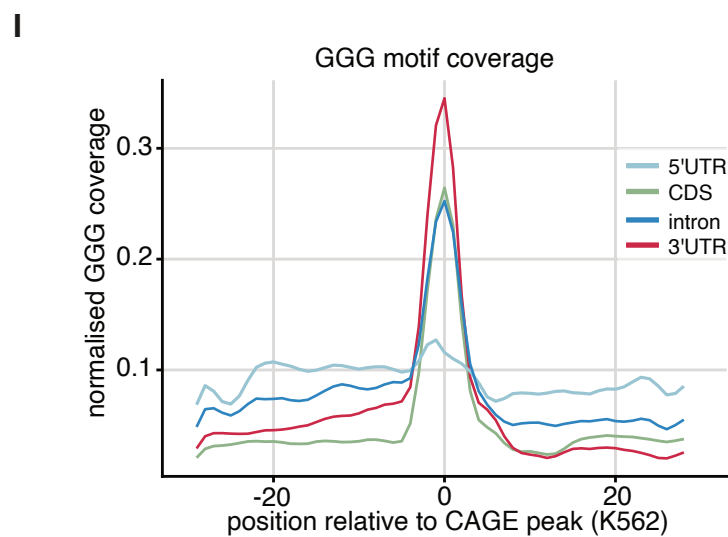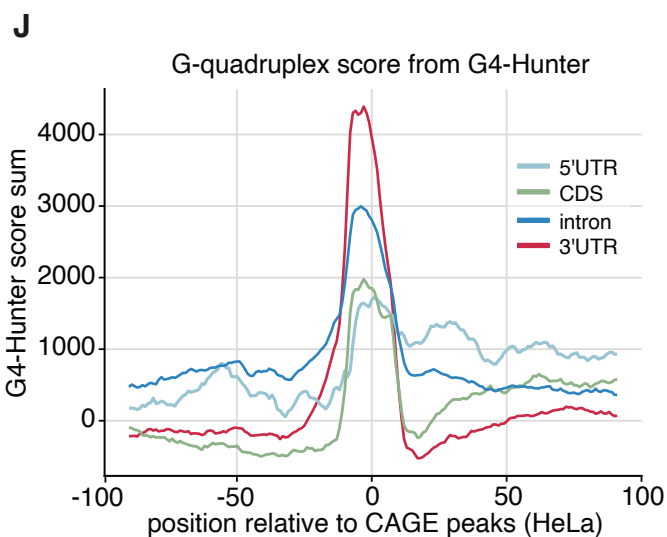

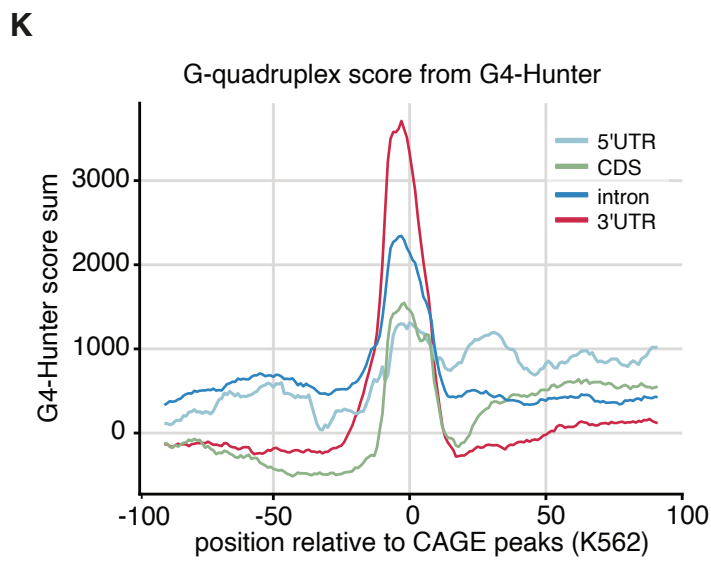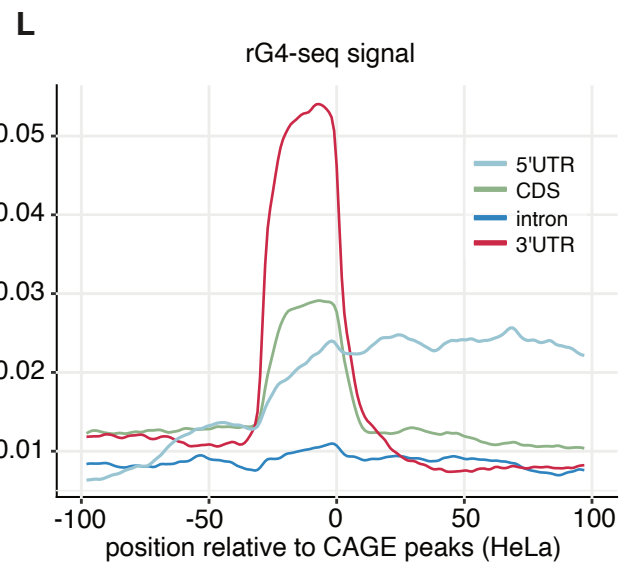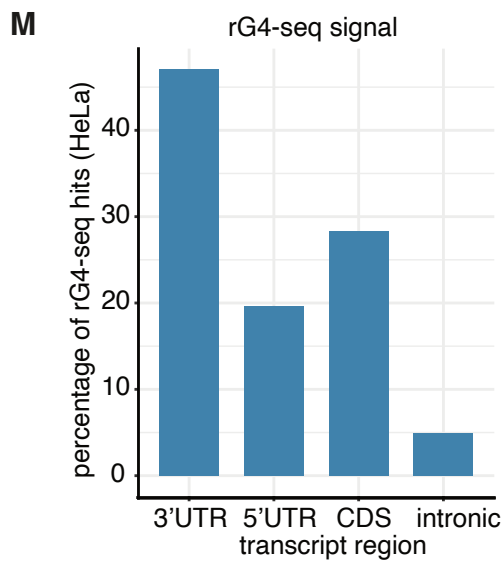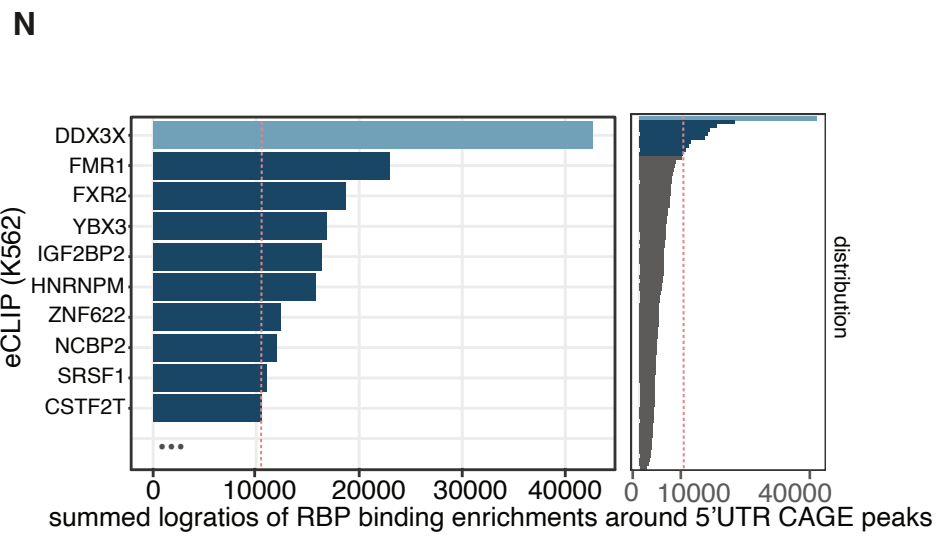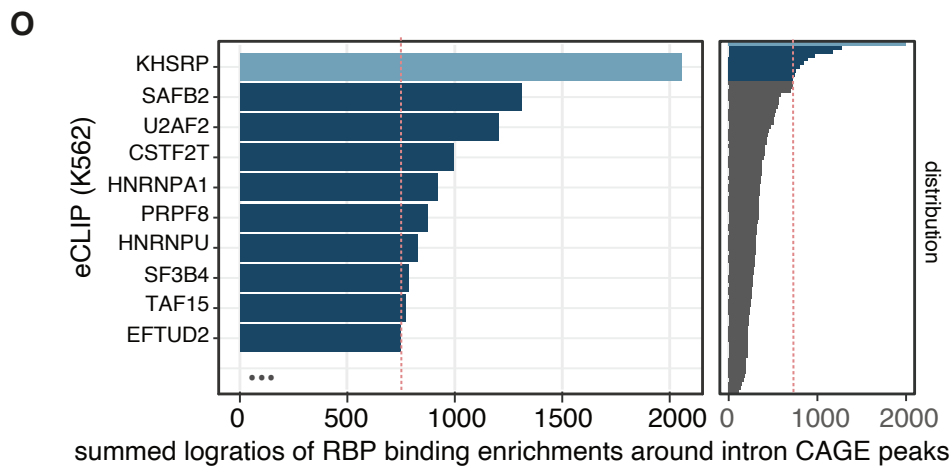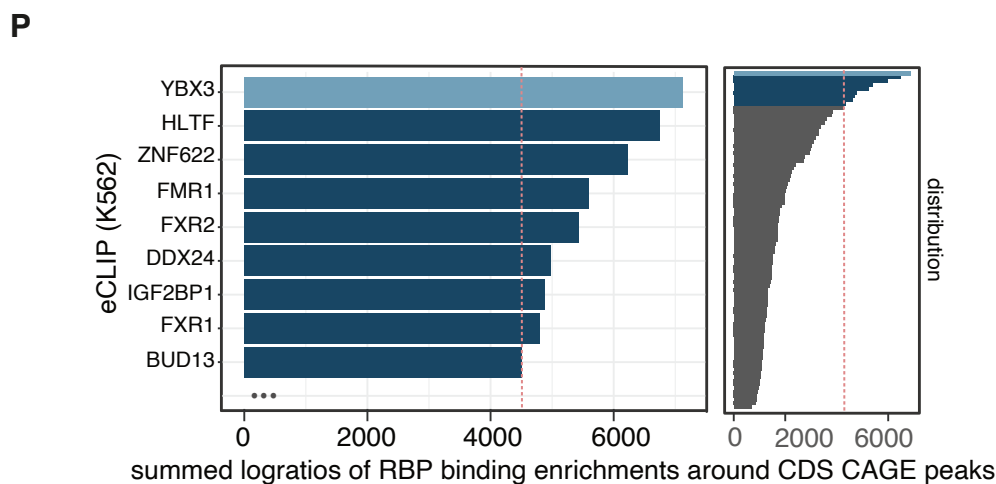

Q

K562:

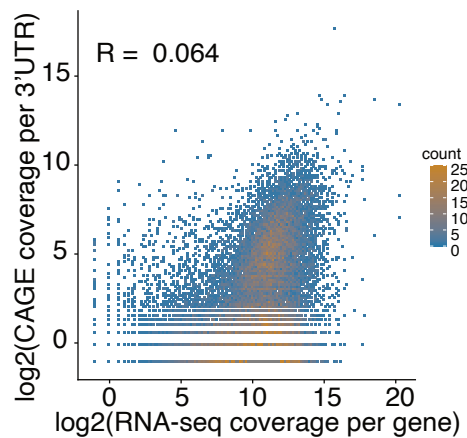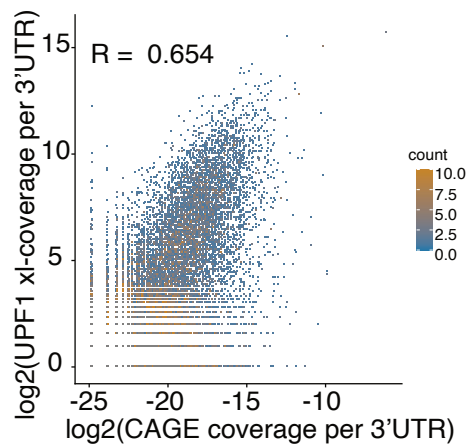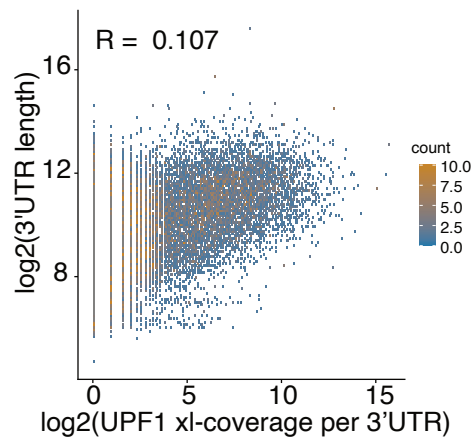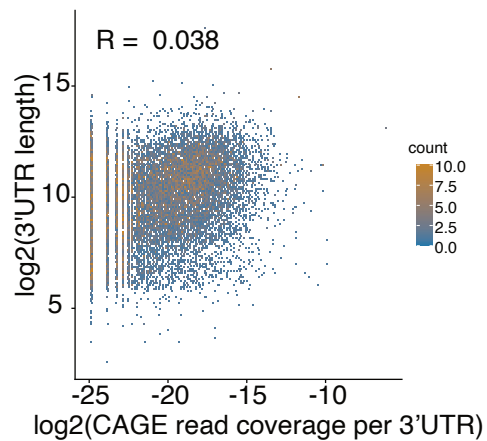

R

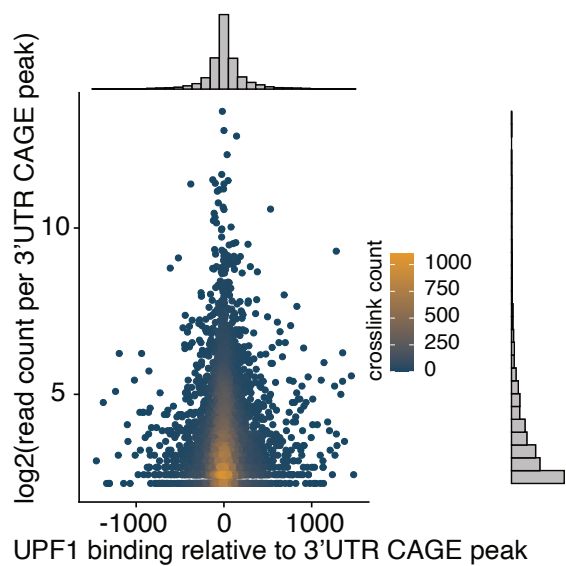

S

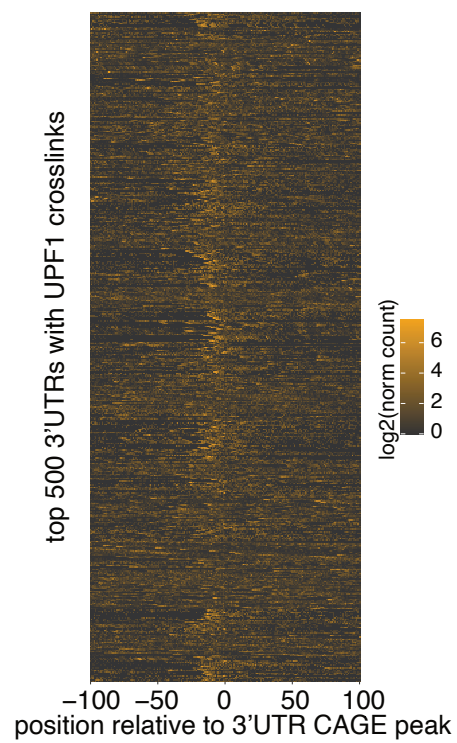

T

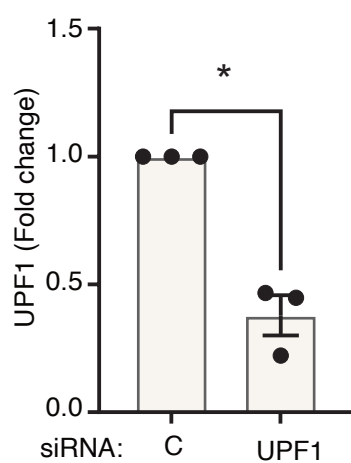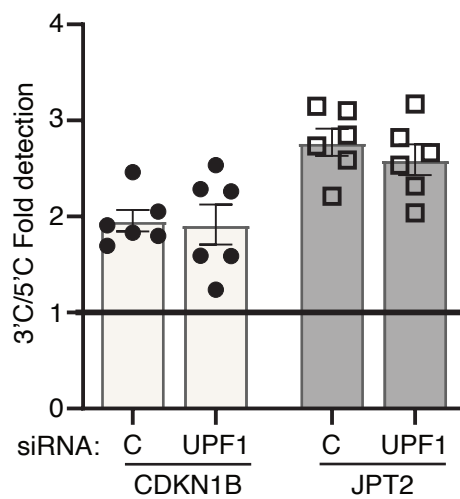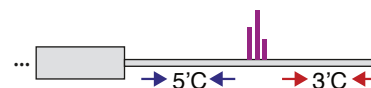

Supplement: Supplementary file 1 — Additional file 1: Figures S1, S2, S3, S4. Each supplementary figure corresponds to the main Figs. in the manuscript, in the same order. Fig. S1 Related to Fig. 1. A Pearson’s correlation of raw CAGE read counts per TSS or consensus cluster across biological replicates and cell types. B Reverse cumulative distribution of CAGE reads after normalisation using CAGEr package [82]. C Total number of CAGE reads in each sample. D Density of total 5′ CAGE read positions normalised by the length of the correspondent transcript region identified in CAGE-seq libraries of K562 and HeLa samples with two biological replicates each, provided by ENCODE. E Percentage of CAGE tags per transcript region using random primers, Oligo(dT) primers, and combination of both primers (1:4 Oligo(dT):Random primers) in CAGE-seq libraries of THP-1 cells generated by RIKEN. F-I Pearson’s correlation between CAGE-seq replicates and different cell lines samples in 3′UTRs, 5′UTRs, CDS and introns. J Top: Plot of the normalised coverage of the 5′ ends of forward paired-end reads (yellow line) and 3′ ends of reverse paired-end reads (blue line) of RNA-seq relative to 3′UTR CAGE peaks in HeLa cells. Bottom: Schematic representation of paired-end read positioning. Forward and reversed paired-end reads are presented in yellow and blue, respectively. The black box represents the ends of reads that are plotted in the top graph. K RT-qPCR data of gene expression using primers designed to amplify sequences located downstream (3'C), upstream (5'C) and overlapping (AC) the 3′UTR CAGE sites of CDKN1B and JPT2. Data represents fold detection (six biological replicates) using downstream versus upstream/overlapping primers relative to the 3′UTR CAGE peaks. Primer target sequences relative to the 3′UTR CAGE peak are schematically represented on the top right-hand side and visualised for each gene using IGV genome browser on the bottom. Each dot represents the value of an independent biological replicate. L Top gene [file 12915_2024_2032_MOESM1_ESM.zip › Additional_File_1_Figure_S2.pdf]

**A**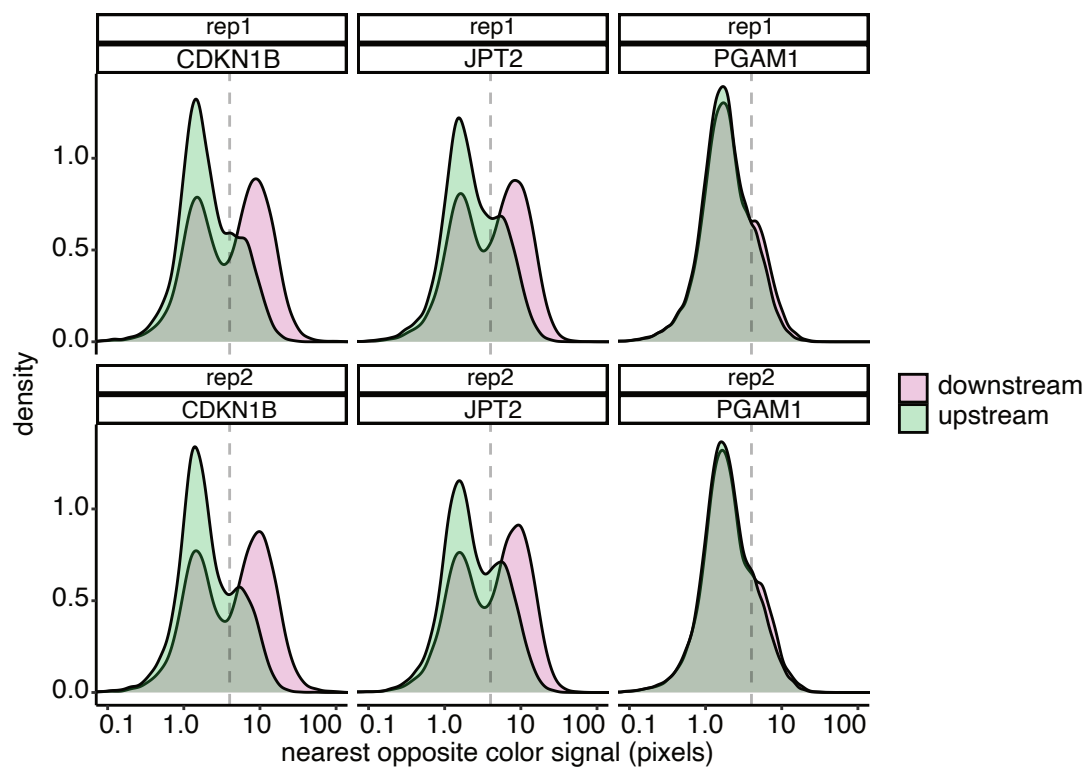

Supplement: Supplementary file 1 — Additional file 1: Figures S1, S2, S3, S4. Each supplementary figure corresponds to the main Figs. in the manuscript, in the same order. Fig. S1 Related to Fig. 1. A Pearson’s correlation of raw CAGE read counts per TSS or consensus cluster across biological replicates and cell types. B Reverse cumulative distribution of CAGE reads after normalisation using CAGEr package [82]. C Total number of CAGE reads in each sample. D Density of total 5′ CAGE read positions normalised by the length of the correspondent transcript region identified in CAGE-seq libraries of K562 and HeLa samples with two biological replicates each, provided by ENCODE. E Percentage of CAGE tags per transcript region using random primers, Oligo(dT) primers, and combination of both primers (1:4 Oligo(dT):Random primers) in CAGE-seq libraries of THP-1 cells generated by RIKEN. F-I Pearson’s correlation between CAGE-seq replicates and different cell lines samples in 3′UTRs, 5′UTRs, CDS and introns. J Top: Plot of the normalised coverage of the 5′ ends of forward paired-end reads (yellow line) and 3′ ends of reverse paired-end reads (blue line) of RNA-seq relative to 3′UTR CAGE peaks in HeLa cells. Bottom: Schematic representation of paired-end read positioning. Forward and reversed paired-end reads are presented in yellow and blue, respectively. The black box represents the ends of reads that are plotted in the top graph. K RT-qPCR data of gene expression using primers designed to amplify sequences located downstream (3'C), upstream (5'C) and overlapping (AC) the 3′UTR CAGE sites of CDKN1B and JPT2. Data represents fold detection (six biological replicates) using downstream versus upstream/overlapping primers relative to the 3′UTR CAGE peaks. Primer target sequences relative to the 3′UTR CAGE peak are schematically represented on the top right-hand side and visualised for each gene using IGV genome browser on the bottom. Each dot represents the value of an independent biological replicate. L Top gene [file 12915_2024_2032_MOESM1_ESM.zip › Additional_File_1_Figure_S4.pdf]
